# Ion Mobility-Mass Spectrometry Strategies to Elucidate the Anhydrous Structure of Noncovalent Guest/Host Complexes

**DOI:** 10.1021/acs.analchem.4c02056

**Published:** 2024-07-16

**Authors:** Jody C. May, Emanuel Zlibut, Benjamin K. Blakley, Constance S. Wood, Yansheng Wei, Brandon Showalter, Eric Dybeck, Emma R. Remish, Valeria Guidolin, Bryan A. Bernat, John A. McLean

**Affiliations:** † Department of Chemistry, Center for Innovative Technology, Vanderbilt University, Nashville, Tennessee 37235, United States; ‡ Pfizer, Inc., Worldwide Research, Development, and Medical, Lake Forest, Illinois 60045, United States; § Pfizer, Inc., Cambridge, Massachusetts 02139, United States; ∥ Pfizer, Inc., Pharmaceutical Sciences Small Molecule (PSSM), Groton, Connecticut 06340, United States

## Abstract

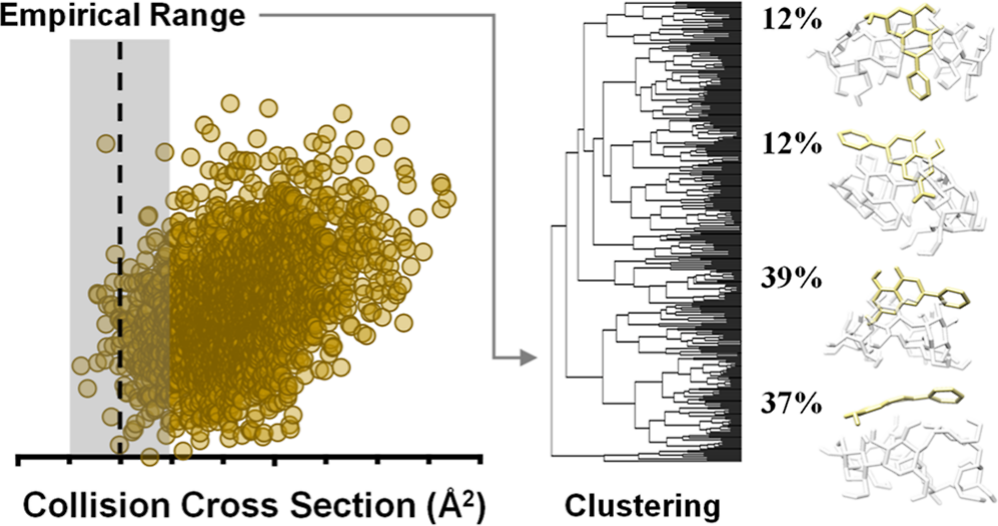

Structural mass spectrometry (MS) techniques are fast and sensitive analytical methods to identify noncovalent guest/host complexation phenomena for desirable solution-phase properties. Current MS-based studies on guest/host complexes of drug and drug-like molecules are sparse, and there is limited guidance on how to interpret MS information in the context of host nanoencapsulation and inclusion. Here, we use structural MS strategies, combining energy-resolved MS (ERMS), ion mobility-MS (IM-MS), and computational modeling, to characterize 14 chemically distinct drug and drug-like compounds for their propensity to form guest/host complexes with the widely used excipient, beta-cyclodextrin (βCD). The majority (11/14) yielded a 1:1 guest/host complex, and ion mobility collision cross section (CCS) analysis provided subtle evidence of gas-phase compaction of complexes in both polarities. The three distinct dissociation channels observed in ERMS (i.e., charged βCD, charged guest, and partial guest loss) were used to direct charge-site assignments for computational modeling, and structural candidates were prioritized using helium-derived CCS measurements combined with root-mean-square distance analysis. The combined analytical information from ERMS, IM-MS, and computational modeling suggested that the majority of anhydrous complexes are inclusion complexes with βCD. Taken together, this work demonstrates a roadmap for how multiple MS-based analytical measurements can be combined to interpret the structures that guest/host complexes adopt in the absence of water.

## Introduction

β-Cyclodextrin (βCD) and its functionalized derivatives are the most widely used ingredients to promote the binding and stability of a broad range of compounds.^1^ βCDs are particularly important for improving the bioavailability of active pharmaceutical ingredients (APIs), the majority of which exhibit poor aqueous solubility.^2^ Common analytical techniques used to characterize guest/host binding in solution include solubility assays, thermal analyses (thermogravimetric measurements, differential thermal analysis, and differential scanning calorimetry), and spectroscopic techniques (UV–vis, IR, circular dichroism, and NMR). These techniques probe bulk phase changes in the physiochemical properties of the sample, which are then used to infer the mechanism of interaction at the molecular level.^3,4^

Mass spectrometry (MS) is a sensitive, gas-phase analysis that provides mass and relative abundance readouts on individual guest/host complexes and their unbound constituents.^5,6^ Because MS analysis occurs after these complexes are transferred from solution to vacuum, only noncovalent complexes which survive phase transfer and ionization are observed in the mass spectrum.^7^ Thus, observation of guest/host complexes in MS data provides direct confirmation that these noncovalent complexes were formed in solution, but MS alone does not provide structural details informing the structure(s) of these complexes. Tandem, multistage MS strategies (MS/MS or MS^n^) can provide specific information regarding the components of noncovalent complexes, and operating tandem MS with incremental changes in the fragmentation energy (energy-resolved MS, ERMS) can yield additional information regarding the relative stability of multiple complexes.^8^ More recently, ion mobility coupled with MS analysis (IM-MS) provides an additional dimension of separation on the basis of anhydrous molecular size and shape, and IM-MS is capable of resolving complexity and provide important structural insights into the gas-phase nature of noncovalent complexes incorporating small molecules.^6^ However, the majority of studies have thus far focused on natural secondary metabolites and amino acid guests.^9,10^ Guest/host IM-MS studies of small molecule-CD complexes are sparse,^11,12^ and guidance on how to interpret IM-MS measurements in the context of guest/host complexation is currently lacking.

In previous work, we evaluated several MS-based techniques for investigating noncovalent guest/host complexes formed between the antimalarial drug, artemisinin, and natural α-, β- and γ-CD hosts.^12^ Here, we explore the application of these established approaches to additional noncovalent systems, which have previously been confirmed to form guest/host complexes with βCD in solution.^4,13−15^ Several anhydrous complexes have also been observed by ESI-MS.^16−18^ The small molecules selected for this study are chemically diverse and exhibit minimal solubility in water (log *P* ≳ 1, Table S1). We validate a previously developed structural MS workflow that includes IM-MS, ERMS, and computational modeling to provide a framework for interpreting this analytical information toward understanding the anhydrous structure of noncovalent guest/host complexes.

## Methods

### Chemicals

Fourteen small molecules, β-cyclodextrin (βCD) and acetate salts of group I alkali metals (LiOAc, NaOAc, KOAc, RbOAc, and CsOAc), were obtained from various commercial sources (Table S1). High purity methanol, water, and formic acid (Optima LC/MS grade) were obtained from Fisher Scientific.

### Sample Preparation

All small molecule-βCD sample solutions were prepared via guest resuspension in aqueous βCD as previously described.^12^ Sample preparation details are provided in the Supporting Information.

### Ion Mobility-Mass Spectrometry

Samples were analyzed via direct infusion (10 μL/min) electrospray ionization (ESI, Jet Stream, Agilent) using a commercial drift tube IM-MS instrument (6560, Agilent Technologies).^19,20^ Instrument settings are provided in Table S2. The drift tube was operated with nitrogen (25 °C, 3.95 Torr) at a fixed dispersion field of 13.44 V/cm (10.5 Td). Measured IM arrival times were converted to collision cross sections (CCS) using a single-field calibration procedure (SF) with the components of a commercial MS tuning mixture serving as calibrants (ESI-L Tuning Mix, Agilent). Reference CCS values were obtained from Stow et al.^21^

### Energy-Resolved Mass Spectrometry

Select guest/host complexes were subject to IM- and MS-resolved ion activation experiments (IM-MS/MS). Target complexes were quadrupole mass selected (4 Da window) and fragmented via collision-induced dissociation (CID) within the collision cell (UHP nitrogen, 22 psi). The collision energy (CE) was varied between 0 and 39 V (laboratory frame) with an energy resolution of 3 V. Precursor abundances were converted to precursor depletion ratios using methods described in the Supporting Information. ERMS data were fitted to a sigmoidal fit.^22^ The relative abundance of precursor and product ions were calculated at 50% depletion (CE_50_) using the sigmoidal fits.

### Computational Modeling

The computational workflow used in this work was adapted from a previous protocol implemented for cation-adducted data and differs only in the evaluation of different protonation sites.^12^ Here, the proton is added in AMBER with guidance from the MS/MS fragment ion data, and multiple likely protonation sites are evaluated. Computational modeling details are provided in the Supporting Information.

## Results and Discussion

**Figure 1:**
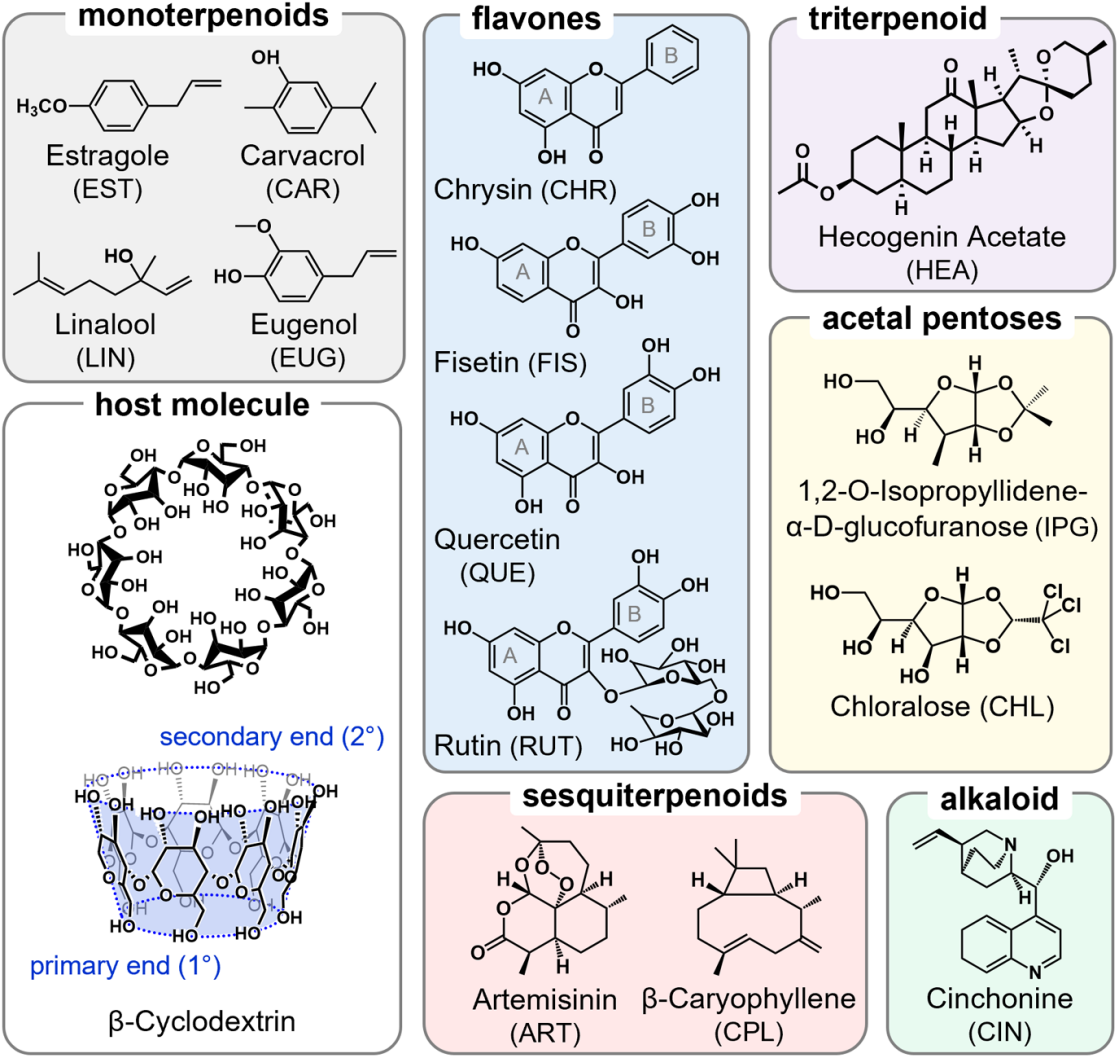
Structures for the guest molecules and the βCD host evaluated in this study.

**Figure 2:**
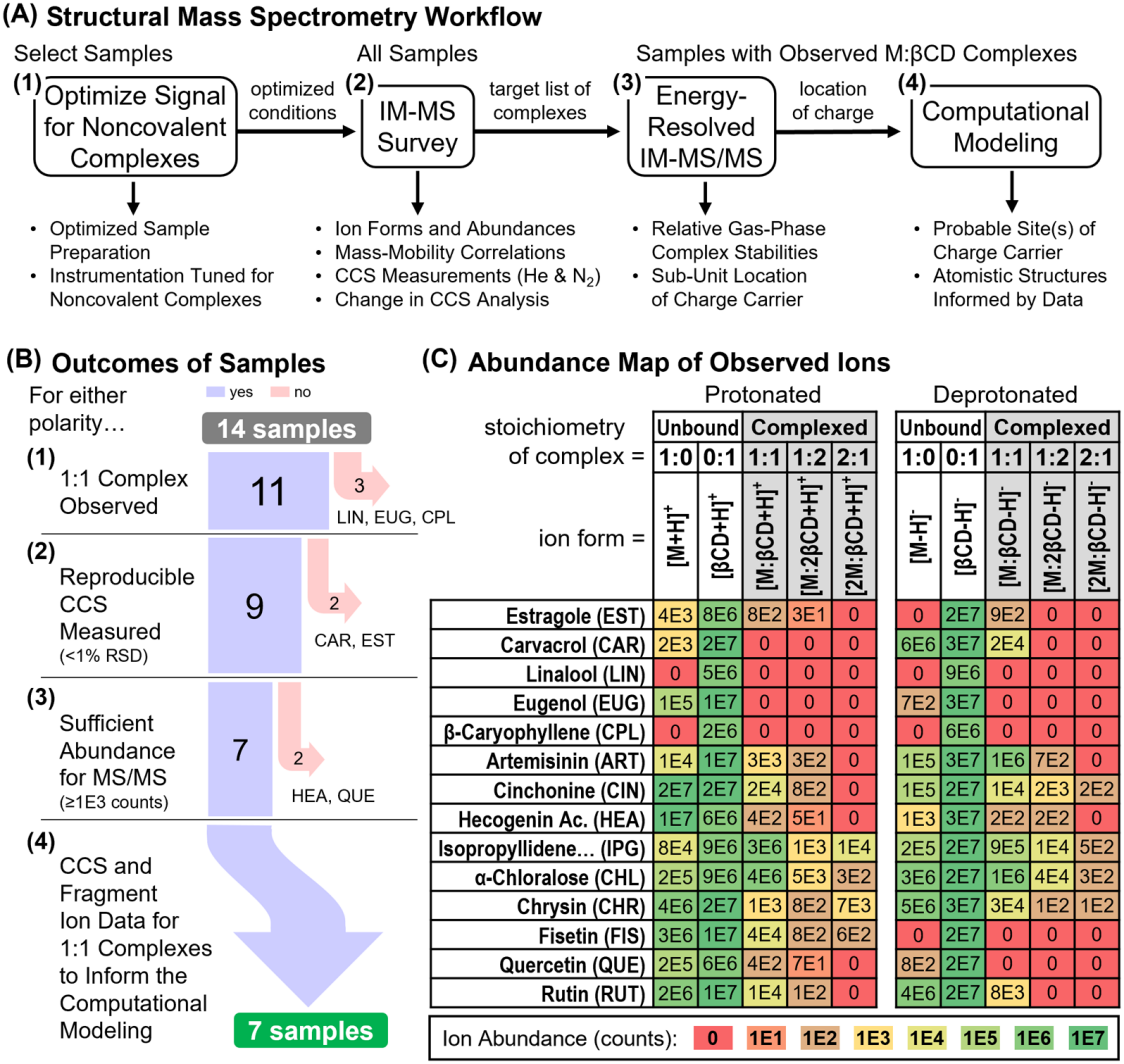
(A) Structural mass spectrometry workflow. (B) Sankey diagram summarizing the outcomes for the 14 small molecule-βCD samples evaluated in this study. (C) Ion abundance summaries for unbound guests and host and their bound guest/host complexes.

Chemical structures for the 14 small molecules and the βCD host are shown in Figure 1, with chemical formulas, masses, and log *P* values provided in Table S1. These molecules were chosen specifically for their chemical diversity and propensity to form solution-phase guest/host complexes with βCD.^4,14,23^ Several have also been examined by gas-phase MS techniques.^16,18,24,25^ The analytical approach for this study (Figure 2A) involves four steps of analysis. Specific outcomes for all 14 small molecule-βCD samples are summarized in Figure 2B for each step of the workflow. Signal optimization experiments (Step 1) are summarized in the Supporting Information.

### Initial Survey of Complexes

Following optimization, all small molecule βCD samples were analyzed by IM-MS in positive and negative ion modes (Figure 2A, step 2). IM-MS spectral results for all sample mixtures and individual components are provided in Figure S3. Most ion forms observed are protonated and deprotonated. Exceptions include IPG, CHL, and RUT systems, where sodium-adducted ion forms are predominant (Figures S3G,M,O). No hydrated ions (+H_2_O) were observed in any spectra. Ion abundance results for ART/βCD are notably similar to previous work on this system; however, the signal enhancement when incorporating lithium appears to be an exception in light of the observations from the other complexes. Lithium was previously shown to enhance the formation of guest–host complexes by widening the opening of the secondary end of βCD to better incorporate the guest,^26^ and the larger size of ART (and HEA) compared to the other analytes in this study may benefit from this cation-induced inclusion structure. A summary of ion abundances for the observed guest/host complexes (1:1, 1:2, and 2:1 stoichiometries) and their unbound constitutes (M and βCD) for both polarities are shown in Figure 2C. These abundances were reproduced across 3–6 interday repeat measurements. The 1:1 complex is the primary stoichiometry found in many solution-phase studies^4,15^ and was observed here for all but three small molecules (LIN, EUG, and CPL), though the majority of M/βCD signals were of relatively low (<1E5) abundances. CPL is a nonpolar hydrocarbon and thus is not expected to yield a strong ion signal by conventional ESI, though CPL/βCD has been reported by GC–MS via electron ionization.^27^ LIN/βCD was previously observed in ESI MS studies,^25^ though both LIN and EUG are notably absent in the ESI-MS analysis of the samples without βCD (Figure S3D,E), which indicate challenges with ionizing and/or transmitting these compounds. The strongest 1:1 complex ion signals were observed for the two acetal pentoses, IPG and CHL with similar abundances in both ion modes. A prior ESI-MS study failed to observe the CHL/βCD ion in the gas phase, even though this complex was predicted to be stable.^28^ ART formed a deprotonated ion complex, [ART/βCD-H]^−^, that was approximately 3 orders of magnitude more intense than the protonated form of the complex ([ART/βCD+H]^+^)—negative ions were not explored in previous work on this system.^12^ The complexes of EST, FIS, and QUE with βCD are only observed in positive ion mode, whereas CAR/βCD is only observed in negative ion mode, suggesting that both ion modes provide complementary analytical information regarding guest/host complexes. In sum, 11 of the 14 small molecules investigated exhibited a 1:1 guest/host complex for conventional protonated/deprotonated ion forms; however, their abundances varied significantly from one system to the other.

### Alkali Cation Investigation

In previous studies using βCD, it was found that lithium promoted the formation of 1:1 guest/host complexes in positive ion mode. The structural basis for this observation as inferred from computational modeling indicated that the cation resides in the smaller primary opening of βCD, which expands the secondary opening to facilitate guest inclusion.^12,26^ To determine if alkali cations provide an enhancement in the ion signal for this work, a charge competition experiment was conducted, whereby an equimolar mixture of five alkali cations were added to an aliquot of each M/βCD sample and analyzed by positive mode IM-MS. Ion abundances are summarized in Figure S4 and indicate that, while in some cases, cation additives enhance formation of the M/βCD ion (e.g., +H for CIN, CHL, and FIS; +Li for ART, HEA, IPG, and CHL; +K for CHR and RUT), overall there does not appear to be a single cation that consistently promotes ionization for all complexes. Interestingly, the majority of complexes show little to no change in their CCS values (Figure S4, lower panel) upon adduction with H, Li, and Na, suggesting that these smaller cations incorporate within the M/βCD complex, as would be expected for cation inclusion within the βCD cavity.^12,26^ Unlike protonated/deprotonated ions, cation-adducted ions exhibit a facile loss of the charge carrier during MS/MS fragmentation; thus, information regarding charge-carrying constituents is lost during cation ejection. This directs the focus of energy-resolved MS/MS studies toward the protonated/deprotonated ions.

### Sample Dilution Study

Prior MS studies on guest/host systems have noted that nonspecific aggregation is sensitive to the concentration of the sample introduced to the ESI source.^7,29^ To test whether the complexes observed in this work might originate from nonspecific clustering, various sample concentrations (5 μM to 5 mM) were evaluated for select 1:1 guest/host systems. The change in ion abundance for unbound guest (guest/host 1:0) and coordinated guest/host complexes (1:1, 2:1, and 1:2) in response to changes in concentration are summarized in Figure S5 and show an expected increase in the signal for the unbound guest, but a relatively unchanged, or sometimes decreased abundance for the bound M/βCD complexes, which suggests that specific guest/host binding is predominant in the corresponding ion signals. This observation is consistent with other ESI-MS studies on guest/host complexes.^16,17,30^

### IM-MS Analysis

IM profiles for the unbound small molecules and their guest/host complexes are summarized in Figure S6. All systems except rutin exhibit single IM peaks, suggesting that these analytes do not adopt multiple gas-phase conformations. The exception, rutin, has a disaccharide functional group (rutinose), which likely contributes to the formation of at least two distinct conformers in its unbound state (observed in both ion modes), though once rutin coordinates with βCD, there is no longer strong evidence for multiple conformers. An unbound βCD host exhibits two structural populations for the protonated ion form (Figure S8) as previous noted,^19^ which have recently been suggested as being “closed” and “open” conformations of cyclodextrin.^31^

**Figure 3:**
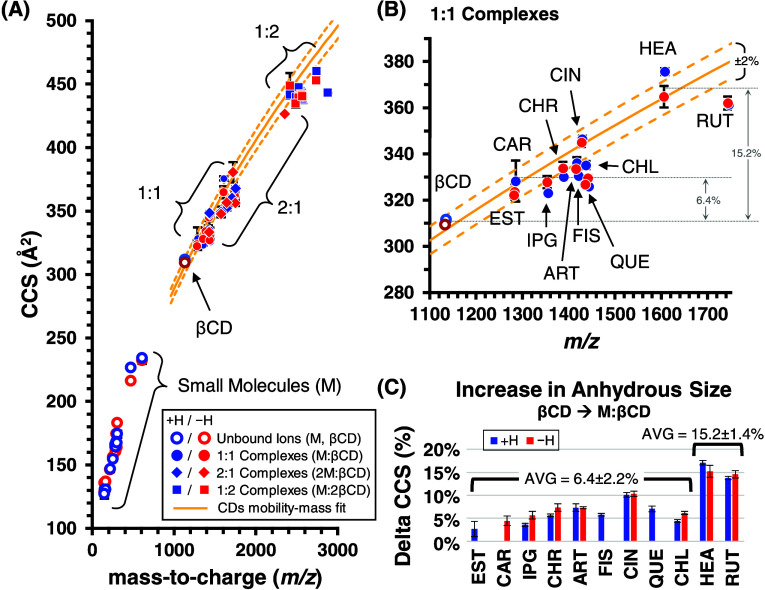
(A) CCS vs *m*/*z* projections of protonated/deprotonated ions, which include unbound small molecules, ([M+H]^+^ and [M–H]^−^), and molecules bound to βCD with various stoichiometries ([nM:mβCD+H]^+^ and [nM:mβCD–H]^−^, *n* = 1, 2; m = 1, 2). (B) Expanded region containing the 1:1 M/βCD ions and (C) the observed change in CCS for βCD binding to each guest. In all plots, a power fit of unbound cyclodextrins (*R*^2^ = 0.9902) is projected with a ±2% band to illustrate the relative gas-phase conformational occupancy of M/βCD complexes.

CCS results for the unbound M, βCD, and coordinated M/βCD complexes with various stoichiometries are summarized in Figure 3 and Table S3. As IM provides structurally averaged snapshots of the gas-phase structure, CCS measurements alone can only be broadly interpreted to discern correlations and trends. For the majority of M/βCD systems, CCS values for both deprotonated and protonated ion forms of the same complex fall within 1% of one another (Table S3), suggesting that similar anhydrous structures are being adopted in both polarities. The smaller unbound analytes (EST, CAR, and EUG) tend to adopt larger CCS values as deprotonated ions, whereas deprotonated ions are smaller for larger analytes (HEA and RUT), though the cause of this observation is unclear. Overall, the CCS measurements adopt a characteristic power law scaling of size and mass in the IM-MS analysis. An overlay of a representative cyclodextrin mobility-mass correlation fit indicates that the coordinated guest/host complexes (1:1, 1:2, and 2:1) adopt slightly more compact structures than cyclodextrins and their aggregates, though the structural compaction of 1:1 complexes is modest, with only about half of these complexes exhibiting CCS values falling outside the empirically derived 2% correlation band (Figure 3B).

The increase in CCS of βCD upon binding each small molecule is summarized in Figure 3C. Here, guests are ordered by mass and the smaller guests exhibit a consistent increase of between 4 and 8% upon complexation, while the largest guests (HEA and RUT) contribute to a ca. 15% increase in the gas-phase size of βCD upon binding. These differential CCS observations are consistent in both ion modes. In previous work from the authors, a similar ∼6% change in CCS was observed for artemisinin binding to βCD, and computational results suggested that ART/βCD was a partial inclusion complex. A similarly modest (5–6%) increase in CCS was also reported by Chen et al. for amino acids (Gly, Leu, and Phe) complexed with βCD, whereas a 9–12% increase was observed for larger amino acids (Leu and Phe) bound to the smaller αCD.^32^ Similar CCS changes of ∼6% were also found in a study of coumaric acid-CD complexes.^9^ In the context of this current work, the small (<8%) change in CCS observed for most guests suggests that many of these anhydrous guest/host complexes are likely inclusion complexes, whereas HEA and RUT are bound in a configuration with βCD that allows these larger guests to significantly contribute to the overall gas-phase structure of their complexes, such as what might be expected from partial or no inclusion into the cyclodextrin cavity. Interestingly, CIN demonstrated an intermediate CCS change (11%) that places these findings more in line with a partially or fully excluded guest/host complex, as suggested by the computational results (Figure 5A).

Some additional structural information can be gained from analyzing the change in relative ordering of each guest molecule upon binding to βCD. Figure S7A demonstrates a highly correlated and expected relationship between small molecule mass and gas-phase size, where the ordering in mass is almost completely conserved in the CCS. In contrast (Figure S7B), once guest molecules bind with βCD, the anhydrous structures change in the relative ordering of their gas-phase sizes. Of note is CHL/βCD, which appears significantly more compact than the other complexes, conforming to a similar CCS as the guest/host complex of its structural analog, IPG/βCD. Another example is HEA and RUT, where RUT/βCD is more compact than HEA/βCD, despite RUT being larger in both mass and CCS than HEA in their unbound, [M+H]^+^ forms. These changes in the relative ordering point to a complicated binding relationship that is specific to each guest/host complex, though in general structurally similar compounds (e.g., CHL and IPG; CHR, FIS, and QUE) exhibit similar changes in CCS upon complexation with βCD, suggesting some conserved structural properties that are class-specific.

### Higher Order Complexes

The importance of higher-order complexes characterized by multiple CDs binding to a single guest (i.e., matrix incorporation vs nanoencapsulation) has been implicated in physiochemical measurements of solution-phase CD complexes.^4,14,33^ Several higher-order complexes were observed in this study (Figure 1B) and are discussed in Appendix 5 and Figure S8 of the Supporting Information. Salient observations of the higher-order complexes are (i) notable gas-phase compaction with respect to the aggregate cyclodextrin mobility-mass fit (Figure 3A) and (ii) minimal change in CCS in response to binding the guest to multiple hosts (e.g., protonated 2βCD → M:2βCD, ΔCCS ∼ 0%, Figure S8A). These observations strongly suggest that these anhydrous higher-order complexes represent fully encapsulated guests.

### Energy-Resolved IM-MS

**Figure 4:**
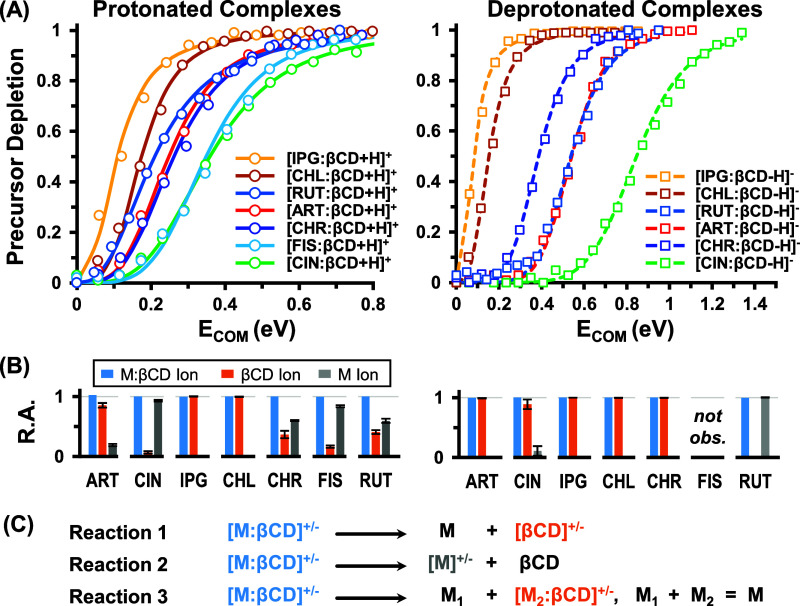
Energy-resolved precursor depletion curves for (A) protonated and deprotonated M/βCD guest/host complexes. (B) Relative abundance (R.A.) of fragment ions observed at 50% depletion. (C) Three dissociation pathways observed for guest/host complexes.

To gain additional insight into the relative structural stabilities of the observed gas-phase guest/host complexes, IM-resolved tandem MS experiments (IM-MS/MS) were conducted on 1:1 complexes exhibiting ion abundances sufficient (≥1E3) to monitor their stepwise dissociation across a range of collision energies (Figure 2A, step 3). Energy-resolved precursor depletion curves for protonated and deprotonated complexes are shown in Figure 4A using an IM filtering strategy to remove interferents co-isolated by the quadrupole (Figure S9). We note no measurable change in precursor CCS across all ERMS energies surveyed, suggesting no additional gas-phase rearrangement occurs. Whereas the magnitude of collision energy needed to dissociate protonated M/βCD complexes is lower than for deprotonated complexes, the relative ordering of stabilities is similar for positive and negative ions, which suggests that similar conformations are adopted in both ion polarities. ERMS results indicate the following anhydrous complex stabilities
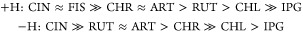


Beyond gleaming relative stability information, a chemical interpretation of the ERMS results is difficult to arrive at, as previous studies have indicated the energy involved in dissociating noncovalent complexes reflects both specific (e.g., inclusion into the host) and nonspecific (e.g., electrostatic binding to the periphery of the host) guest/host interactions and do not necessarily correlate to solution-phase binding.^5,29^

In addition to relative stabilities, ERMS data provide useful information regarding probable location(s) of the charge carrier that can inform the assembly of theoretical structures. The dissociative ion channels observed for these complexes are monitored in ion abundance plots (Figure S10) and relative abundances (R.A.) at ca. 50% depletion (Figure 4B). The dissociation of the M/βCD noncovalent complexes results in three possible reaction channels (Figures 4C and S11): (1) dissociation that yields a charged host, [βCD+H]^+^ or [βCD–H]^−^, (2) dissociation that yields a charged guest, [M+H]^+^ or [M–H]^−^, and/or (3) dissociation where partial fragmentation of the guest is observed, leaving part of the guest/host complex intact. These three dissociation pathways were also noted by Rosu et al. for noncovalent drug–DNA complexes.^34^

In all systems investigated by ERMS, the charged βCD host is always observed in both ion modes (reaction 1), indicating a high preference for the charge to reside on βCD. Whether this charge localization on βCD is inherent to the intact complexes or is mobilized during ion activation is unclear, though in general, protonation and deprotonation are not preferred ion forms for βCD.^12^ In most cases (ART, CIN, CHR, FIS, and RUT), the protonated guest ([M+H]^+^) is observed as a product ion (reaction 2), which indicates that the proton can also localize on the guest molecule in these systems. These five systems also exhibit the highest stability (Figure 4A), with the two most stable, FIS/βCD and CIN/βCD, also exhibiting the highest abundances of the protonated guest at 50% depletion (Figure 4B). This observation suggests that the proton-carrying capability of the guest is linked to the stability of the anhydrous complex. For deprotonated complexes, only CIN/βCD and RUT/βCD yielded a deprotonated guest as a fragment channel, and CIN/βCD was found to be the most stable deprotonated complex. RUT/βCD is a more complicated case, as discussed below.

Rutin is a functionalized form of quercetin, where the hydroxyl group at the C-3 position is substituted with the disaccharide, rutinose (Figure 1). Dissociation of the [RUT/βCD+H]^+^ complex results in two high abundance product ions, which represent partial cleavage of the rutin molecule, as it remains bound with βCD (reaction 3), namely, *m*/*z* 1443 (βCD+glucose) and *m*/*z* 1281 (βCD+rutinose), which correspond to neutral losses of quercetin + rhamnose and the quercetin flavonol scaffold, respectively (Figure S11A). The observation of these two product ions indicates a strong association between the glycone and βCD, which does not correspond to inclusion of the quercetin moiety of rutin into the βCD cavity. These results stand in contrast to the findings of Guo et al., where dissociation of [RUT/βCD+Na]^+^ yielded a prominent fragment ion representing quercetin inclusion in βCD.^30^ Fragmentation of alkali-adducted RUT/BCD complexes in this work failed to reproduce partial fragments that are diagnostic of inclusion phenomena (Figure S12). These fragment ion discrepancies point to the importance of the charge carrier (i.e., proton or alkali cation) as directing the specific conformation of the noncovalent complex formed in the gas phase. In negative ion mode, the intact [RUT–H]^−^ ion is the predominant fragment observed, with [βCD–H]^−^ appearing only in trace abundances at higher CEs (Figure S11B), indicating a strong preference for deprotonation at the RUT guest. The lack of a partial fragment of rutin in negative mode implies a weaker association of rutin with βCD than was observed in positive mode.

### Computational Findings

Seven systems which formed protonated gas-phase guest/host complexes with sufficient abundance for MS/MS studies (i.e., M/βCD, M = ART, CIN, IPG, CHL, CHR, FIS, and RUT) were further investigated using theoretical structural modeling. Full computational results for these seven systems are summarized in Figure S13. Conformational scatter plots of predicted CCS vs relative energy are generated for each probable site of protonation, as guided by the ERMS results. The computed relative energies for each protonation site evaluated are consistent with experimental observations. For example, the lowest energy structural family for CIN/βCD, CHR/βCD, FIS/βCD, and RUT/βCD is for protonation on the guest molecule, and the corresponding MS/MS results show prominent [M+H]^+^ guest ions as fragments.

**Figure 5:**
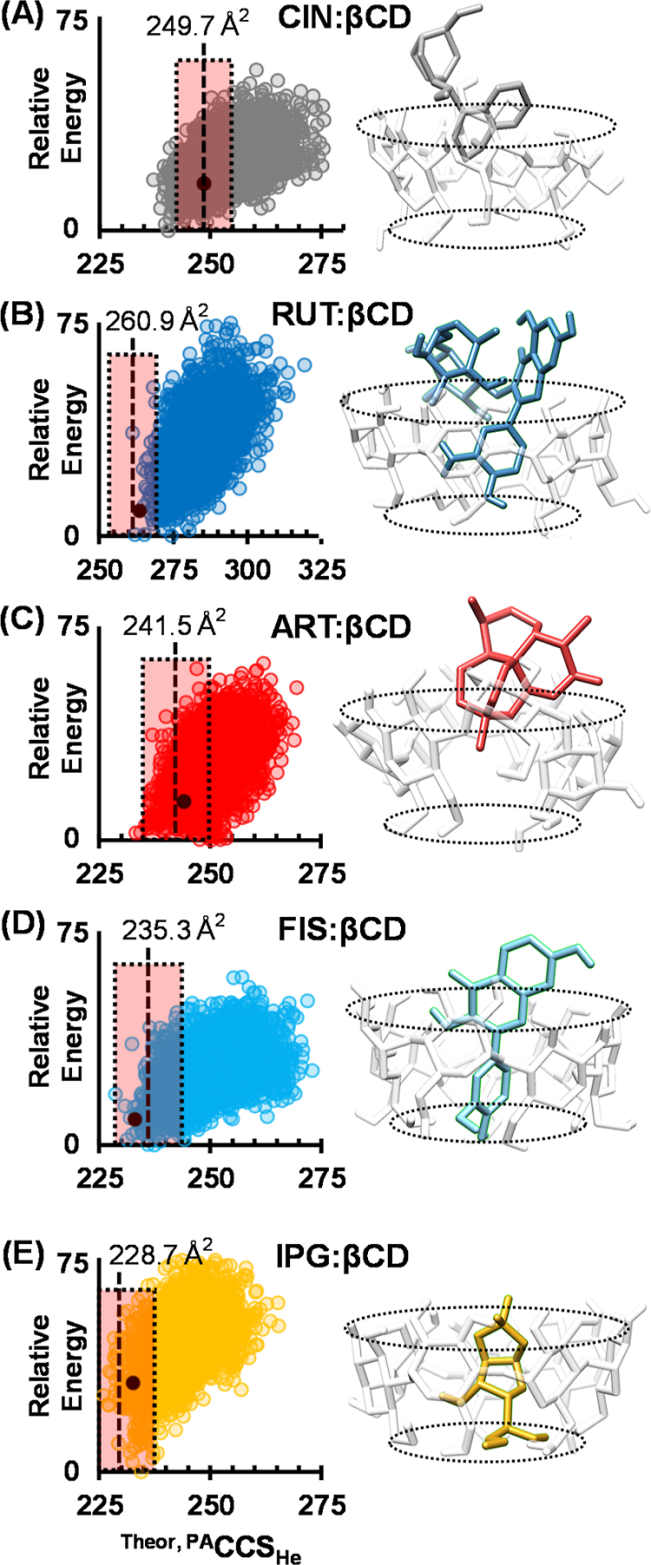
Theoretical conformational space plots and CCS-aligned candidate structures for protonated (A) CIN/βCD, (B) RUT/βCD, (C) ART/βCD, (D) FIS/βCD, and (E) IPG/βCD. Vertical dotted lines represent helium CCS measurements with shaded boxes representing ±3%. The dark points correspond to the structures shown for each panel. These five examples represent various degrees of encapsulation by the βCD host molecule.

Theoretically predicted CCS values span a wide range, and most are larger than what is experimentally observed, which is consistent with previous findings.^12,35^ Predicted structures are thus prioritized based on experimental CCS measurements conducted in helium. Helium CCS values are highly correlated to nitrogen CCS values in this work (ΔCCS 33.8 ± 0.8%, Table S4), which indicates that the same structures are being measured in both drift gases. Here, theoretical low-energy structures falling within ±3% of the experimental CCS were subjected to clustering analysis based on the root-mean-square distance of atoms from superimposed structures. Structural families were identified from the dendrograms and visually evaluated for guest inclusion into the βCD host (Figure S13, Table 1). For all but one of these guest/host systems, the ±3% alignment to experiment excludes the majority of candidate structures. CIN/βCD is the exception, where the CCS alignment step prioritizes over half (65%) of the candidates, though nearly all (96%) of the ca. 2000 prioritized CIN/βCD structures have some amount of guest inclusion into βCD. For all systems, most CCS-aligned structures can be characterized as inclusion complexes. Importantly, all inclusion complexes were predicted to be on average lower in energy than non-inclusion complexes (Figure S12, panel 4), which suggests that structures which incorporate some degree of guest inclusion are the preferred anhydrous structures observed in IM-MS analysis. Computational results for five exemplary guest/host systems are shown in Figure 5 and discussed below. Structures shown are obtained near the average energy within each structural family (Figure S13).

**Table 1:** Summary of CCS-Aligned Theoretical Structures Exhibiting Inclusion Phenomena

guest/host complex	number of structures which align to ±3% of experimental ^DT^CCS_He_	number of aligned structures exhibiting inclusion
ART/βCD	443/3000 (15%)	355/443 (80%)
CIN/βCD	1946/3000 (65%)	1865/1946 (96%)
IPG/βCD	230/3000 (8%)	222/230 (97%)
CHL/βCD	42/3000 (1%)	20/42 (48%)
CHR/βCD	123/3000 (4%)	78/123 (63%)
FIS/βCD	210/3000 (7%)	70/210 (33%)
RUT/βCD	17/3000 (<1%)	17/17 (100%)

The CCS-aligned candidate structure for CIN/βCD (Figure 5A) indicates partial guest inclusion via the naphthyl (quinoline) group, which is the lower-energy orientation discussed by Wen et al. in prior ESI-MS studies of this system.^16^ This orientation accounts for the majority (96%) of predicted structures for this system (Figure S13B).

The representative aligned structure for RUT/βCD (Figure 5B) predicts partial inclusion of the dihydroxybenzene on the flavone scaffold (the “B” ring, Figure 1), with the rutinose disaccharide coordinating externally to the secondary end of βCD, leaving the “A” ring portion (dihydroxychromone) outside of βCD. NMR studies on other flavones in solution (naringenin, naringin, hesperetin, and dihydromyricetin) correspond to the anhydrous structures found here, specifically with the “B” ring incorporated within βCD.^36,37^ However, while “B” ring inclusion is the lowest energy gas-phase conformation, a second RUT/βCD orientation with “A” ring inclusion is also predicted within the experimental CCS range (Figure S13G). These two structural families may correlate to the two peaks measured for unbound rutin (Figure S7A), though the bound [RUT/βCD+H]^+^ ion complex data only hints at the possibility of multiple structures by means of a weak shoulder feature on the IM profile (Figure S7B). As a caveat, only a small fraction of the theoretical conformational space calculated for RUT/βCD overlaps with the empirical CCS measurements (17 out of 3000, or 0.6%), which limits the pool of candidate structures evaluated. We interpret this low coverage as a result of the large conformational flexibility of rutin-βCD, which may represent a system where our theoretical approach is reaching the limits of accuracy. A similarly low structural coverage is also observed for CHL/βCD (Figure S13D), where chlorine is not specifically parametrized in our theoretical method.

For ART/βCD (Figure 5C), the methyl adjacent to the endoperoxide-containing oxepane group extends into the βCD cavity, which allows the trioxane to coordinate with the βCD secondary hydroxyls. This results in a partially included complex where ART extends outside of βCD. Previous work with ART/βCD found that lithium facilitated ART inclusion into βCD with the ketone group oriented inside the βCD cavity toward the primary hydroxyl groups, whereas here the ketone is interacting with the secondary hydroxyls and the peroxide is now inserting into βCD. As a result, the candidate structure found in the previous work has the guest included more into the βCD than the structure found here; however, both predicted ART/βCD structures are partial inclusion complexes.^12^

For FIS/βCD (Figure 5D), the flavone orientation is similar to the findings for rutin where the “B” ring is inserted into the βCD cavity, but fisetin lacks a glycoside functionality, which allows fisetin to incorporate more fully into βCD. The FIS/βCD candidate structure is similar to the solution-phase structure implied by NMR studies for other flavones^37^ and is remarkably similar to the solution-phase structure proposed for luteolin/βCD, where luteolin differs from FIS only in the location of a single hydroxyl on the B-ring of the flavone backbone.^38^

Finally, the computational findings for IPG/βCD (Figure 5E) suggest a fully included guest/host complex with the IPG hydroxyl groups coordinating with the primary βCD face, allowing the dioxolane to coordinate with the secondary βCD face. CHL, the structural analog of IPG, exhibits a similarly encapsulated inclusion complex, though the three chlorine functional groups in CHL limit the accuracy of the CCS alignment (∼1%, Figure S13D).

In general, all the predicted guest/host complexes indicate a strong preference for the guest to reside at the larger secondary end of βCD, which is consistent with solution-phase findings, where βCD encapsulation is facilitated via the larger 2° cyclodextrin opening.^39^ Within the same chemical classes, similar guest orientations are observed. For example, both acetal pentoses (IPG and CHL) “thread” the host βCD with the same hydroxyl and acetal group orientations. For the flavones, all proposed structures show B ring incorporation within βCD, including CHR which lacks B-ring hydroxyls. The two lowest-energy structural families for CHR/βCD (I and II, Figure S13E) predict inclusion of CHR in two orientations: B-ring insertion or A-ring insertion via the R^7^ hydroxyl group. Structure I is similar to the solution-phase structure proposed by Liu et al. for baicalin/βCD, which is a flavone that also lacks B-ring hydroxyls.^40^ For ART and CIN, the peroxide bridge and quinoline groups, respectively, behave in a similar manner as the hydroxyl groups on the other guests, forming ion–dipole interactions with the secondary βCD hydroxyls. Collectively, these observations can inform the structural interpretation of future guest/host experiments.

## Conclusions

Structural MS techniques including ERMS and IM-MS provide sensitive and high-throughput readouts of the existence of noncovalent guest/host complexes, which survive transfer and dehydration from solution to the gas phase. The examination of several different compounds complexed with βCD offers a broad analytical picture of how various chemical systems present themselves in the gas phase. Here, we find that most systems exhibit a protonated 1:1 guest/host complex via ESI. While the addition of alkali cations improved ion signal for specific complexes, there was no single cation that provided an overall enhancement for all complexes. Initial ERMS experiments gave complicated spectra due to isobaric interferences that are prevalent in these βCD samples; however, the addition of ion mobility separations prior to tandem MS2 (IM-MS/MS) partitioned signals of interest from the chemical noise and allowed “authentic” ERMS data to be obtained. Once IM-filtered, precursor breakdown curves reveal three possible dissociation channels for guest/host complexes based on whether the charged host, charged guest, or partial dissociation of the guest is observed as a fragment ion. In the specific case of RUT/βCD, partial fragmentation differed depending on whether a protonated or cation-adducted complex was selected. These findings paint a complicated picture of charge-directed anhydrous conformations being adopted by these complexes.

The conclusions drawn from the computational findings is that the majority of structures predict some level of guest inclusion into the βCD host, though other guest-excluded structures are also predicted for several systems, implying the possibility of multiple anhydrous structures with distinctly different conformations existing within the range of gas-phase measurements. Anhydrous inclusion complexes are likely stabilized from solution-phase inclusion complexes, though the possibility of nonspecific complexes rearranging into inclusion complexes has also been suggested.^29,41^ While our interpretation is that guest inclusion represents the predominant conformations among the anhydrous M/βCD complexes sampled, the possibility of distinct, charge-directed configurations is also suggested by our results. The high sensitivity and throughput achieved by contemporary multidimensional MS-based methods enables numerous guest/host systems to be studied toward gaining a more comprehensive understanding of how noncovalent complexes behave in the absence of solvent. As these technologies continue to improve in the context of sensitivity, resolution, and minimal ion heating, the analytical information garnered by energy-resolved IM-MS will provide deeper structural insights into the nature of noncovalent complexation.

## Supporting Information Available

Details on chemicals used in this work, methanol composition, guest/host ratio, cation competition, sample dilution study results, instrument parameters, IM-MS spectra, IM profiles, CCS measurement results, comments on higher order complexes, delta CCS analyses, MS/MS and ERMS data, computational results for select guest/host systems, and corresponding GAFF parameter files (PDF)

## Abbreviations

αCD: alpha-cyclodextrin
AMBER: Assisted Model Building with Energy Refinement
API: active pharmaceutical ingredient
ART: artemisinin
βCD: beta-cyclodextrin
CAR: carvacrol
CCS: collision cross section
CIN: cinchonine
CHL: α-chloralose
CHR: chrysin
CID: collision-induced dissociation
CPL: β-caryophyllene
ECOM: center-of-mass collision energy
ERMS: energy-resolved mass spectrometry
ESI: electrospray ionization
EST: estragole
EUG: eugenol
FIS: fisetin
γCD: gamma-cyclodextrin
GC-MS: gas chromatography–mass spectrometry
HEA: hecogenin acetate
IM-MS: ion mobility-mass spectrometry
IPG: 1,2-*O*-isopropyllidene-α-d-glucofuranose
IR: infrared spectroscopy
LIN: (±)-linalool
MS: mass spectrometry
MS/MS: two-stage tandem mass spectrometry
MSn: multistage tandem mass spectrometry
NMR: nuclear magnetic resonance spectroscopy
PA: projection approximation method
QUE: quercetin
RMSD: root-mean-square deviation
RUT: rutin
SF: single-field CCS calibration
UV–vis: ultraviolet–visible spectroscopy
