# Supplementary material for: Ion Mobility-Mass Spectrometry Strategies to Elucidate the Anhydrous Structure of Noncovalent Guest/Host Complexes
Source: Anal Chem. 2024 Jul 16;96(30):12453–62. doi: 10.1021/acs.analchem.4c02056 (PMC11295130; doi:10.1021/acs.analchem.4c02056)
Supplement: Supplementary file 1 — ac4c02056_si_001.pdf [file ac4c02056_si_001.pdf]

**Supporting Information**

# Ion Mobility-Mass Spectrometry Strategies to Elucidate the Anhydrous Structure of Noncovalent Guest:Host Complexes

Jody C. May,[†] Emanuel Zlibut,[†,Δ] Benjamin K. Blakley,[†] Constance S. Wood,[‡] Yansheng Wei,[‡] Brandon Showalter,[‡] Eric Dybeck,[§] Emma R. Remish,[‡, ΔΔ] Valeria Guidolin,[§] Bryan A. Bernat,[‡] and John A. McLean[*,†]

[†] Department of Chemistry, Center for Innovative Technology, Vanderbilt Institute of Chemical Biology, Vanderbilt Institute for Integrative Biosystems Research and Education, Vanderbilt University, Nashville, Tennessee 37235, United States.

[‡] Pfizer, Inc. Worldwide Research, Development & Medical, Lake Forest, Illinois 60045 United States.

[§] Pfizer, Inc. Pharmaceutical Sciences Small Molecule (PSSM), Groton, Connecticut 06340 United States.

[Δ] Current Address: MOBILion Systems, Inc. 4 Hillman Drive, Suite 130, Chadds Ford, Pennsylvania, 19317, United States.

[ΔΔ] Current Address: Vanderbilt University, Nashville, Tennessee 37235, United States.

*Corresponding Author Email: john.a.mclean@vanderbilt.edu

**Table S1.** Chemicals used in this work with their identifiers, vendor sources, and calculated log*P* values. A log*P* <5 is one of the "rule of five" for successful pharmaceutical development.(Lipinski *et al.*, 2012)

| | | | Molecular Formula | Exact Mass (Da) | PubChem CID | Vendor Source (Product #) | log*P* Value[a] |
|---|---|---|---|---|---|---|---|
| Small Molecules (Guests) | **Estragole** | **EST** | $C_{10}H_{12}O$ | 148.09 | 8815 | Millipore-Sigma (34098) | 3.4 |
| | **Carvacrol** | **CAR** | $C_{10}H_{14}O$ | 150.10 | 10364 | Millipore-Sigma (W224511) | 3.1 |
| | **Eugenol** | **EUG** | $C_{10}H_{12}O_2$ | 164.08 | 3314 | Millipore-Sigma (E51791) | 2 |
| | **Artemisinin** | **ART** | $C_{15}H_{22}O_5$ | 282.15 | 68827 | Millipore-Sigma (361593) | 2.8 |
| | **Cinchonine** | **CIN** | $C_{19}H_{22}N_2O$ | 294.17 | 90454 | Millipore-Sigma (27370) | 2.7 |
| | **Hecogenin Acetate** | **HEA** | $C_{29}H_{44}O_5$ | 472.32 | 101906 | TCI America (H0537) | 5.4 |
| | **1,2-O-isopropylidene-α-D-glucofuranose** | **IPG** | $C_9H_{16}O_6$ | 220.09 | 219905 | Millipore-Sigma (31460) | -0.3 |
| | **α-Chloralose** | **CHL** | $C_8H_{11}Cl_3O_6$ | 307.96 | 7057995 | Millipore-Sigma (C0128) | 1 |
| | **Chrysin** | **CHR** | $C_{15}H_{10}O_4$ | 254.06 | 5281607 | Millipore-Sigma (C80105) | 2.1 |
| | **Fisetin** | **FIS** | $C_{15}H_{10}O_6$ | 286.05 | 5281614 | Millipore-Sigma (PHL82542) | 2 |
| | **Quercetin** | **QUE** | $C_{15}H_{10}O_7$ | 302.04 | 5280343 | Millipore-Sigma (PHL89262) | 1.5 |
| | **Rutin** | **RUT** | $C_{27}H_{30}O_{16}$ | 610.15 | 5280805 | Millipore-Sigma (R5143) | -1.3 |
| | **(±)-Linalool** | **LIN** | $C_{10}H_{18}O$ | 154.14 | 6549 | Millipore-Sigma (L2602) | 2.7 |
| | **β-Caryophyllene** | **CPL** | $C_{15}H_{24}$ | 204.19 | 5281515 | Millipore-Sigma (W225207) | 4.4 |
| Host | **β-Cyclodextrin** | **βCD** | $C_{42}H_{70}O_{35}$ | 1134.37 | 444041 | Millipore-Sigma (1154569) | -15 |
| Acetate Salts | **Lithium Acetate** | **LiOAc** | $Li(CH_3CO_2)$ | 66.03 | 3474584 | Thermo Scientific (297110250) | -- |
| | **Sodium Acetate** | **NaOAc** | $Na(CH_3CO_2)$ | 82.00 | 517045 | Millipore-Sigma (241245) | -- |
| | **Potassium Acetate** | **KOAc** | $K(CH_3CO_2)$ | 97.98 | 517044 | Thermo Scientific (AA13449) | -- |
| | **Rubidium Acetate** | **RbOAc** | $Rb(CH_3CO_2)$ | 143.93 | 23673628 | Thermo Scientific (AA12890) | -- |
| | **Cesium Acetate** | **CsOAc** | $Cs(CH_3CO_2)$ | 191.92 | 5152919 | Thermo Scientific (AC39083) | -- |

[a.] Computed by XLogP3 3.0 (PubChem release 2021.10.14), www.pubchem.com

**Table S2.** IM-MS Instrument Settings (Agilent 6560 IM-QTOF) Used in This Work.

| | Instrument Parameter | Value |
|---|---|---|
| Ion Source | Sheath Gas Flow | 11 L/min |
| | Sheath Gas Temperature | 275 °C |
| | Nebulizer Pressure | 25 psi |
| | Drying Gas Flow | 5 L/min |
| | Drying Gas Temperature | 325 °C |
| | Ion Focusing Nozzle | 2000 V |
| | Ion Transfer Capillary (VCap) | 3800 V |
| | Fragmentor | 400 V |
| Dual Funnel Stage | High Pressure Funnel Delta | 150 V |
| | High Pressure Funnel RF | 200 $V_{pp}$ |
| | High Pressure Funnel Pressure | 4.4 Torr |
| | Trap Funnel Delta | 180 V |
| | Trap Funnel RF | 200 $V_{pp}$ |
| | Trap Entrance | 91 V |
| | Trap Exit | 90 V |
| | Trap Funnel Exit | 10 V |
| | Trap Funnel Pressure | 3.8 Torr |
| | Trap Fill Time | 50 ms |

| | Instrument Parameter | Value |
|---|---|---|
| Drift Tube | Trap Release Time | 200 µs |
| | Drift Tube Entrance | 1272 V |
| | Drift Tube Exit | 222 V |
| | Rear Funnel Entrance | 215.5 V |
| | Rear Funnel RF | 180 $V_{pp}$ |
| | Rear Funnel Exit | 43 V |
| | Drift Tube Pressure | 3.95 Torr |
| | Drift Field[a] | 13.44 V/cm |
| | Drift Gas | nitrogen |
| Acquisition | Ion Mobility Dispersion Range | 0-80 ms |
| | IM Spectral Acquisition Rate | 12 Hz |
| | TOF Spectra per IM Spectra | 490 |
| | TOF Acquisition Rate | 5.88 kHz |
| | TOF Mass Dispersion Range | *m/z* 50-3200 |

[a] Drift field calculated using a drift length of 78.12 cm

## Appendix 1 – Preparation of Small Molecule-βCD Sample Mixtures

Three stock solutions (0.001 M, 0.005, and 0.025 M) of each guest were prepared in methanol, vacuum dried, then reconstituted with a 0.01 M aqueous solution of βCD. The solution was stirred at room temperature for three days then filtered (0.45 μm) to yield three small molecule:βCD mixtures of 5:1, 1:1, and 1:5. For IM-MS analysis, 10 μL of each sample mixture was added to 1 mL of 3:2 methanol:water solvent with 0.1% formic acid. For cation competition experiments, an equimolar cation mixture of the five alkali acetate salts was prepared in 3:2 methanol:water to 1 mM. Next, 5 μL aliquots of each 1 mM small molecule:βCD (1:1) stock solution were transferred to individual microcentrifuge tubes to which 5 μL of the 1 mM cation mixture and 1 μL of formic acid was added to each. These cation-enriched sample solutions were then diluted 1:100 using 1.1 mL of 3:2 methanol:water to yield a final concentration of 10 μM (M:βCD) for IM-MS analysis.

**Appendix 2 - Optimization of MS Signal**

In the initial step of the workflow (**Figure 2A-1**, in main text), a small molecule-βCD system is selected for initial optimization of sample preparation and IM-MS instrument tuning parameters. A full range of methanol compositions between 0-100% were evaluated using four analyte-βCD mixtures incorporating ART, CHL, CIN, and RUT as guests in 1:1 molar ratios with βCD. A 60% methanol solution was found to form the highest abundance of protonated ion signal, considering both unbound ($[M+H]^+$, $[βCD+H]^+$) and noncovalently-bound guest:host 1:1 and 1:2 complexes ($[M:βCD+H]^+$, $[M:2βCD+2H]^{+2}$), with only minimal (<0.3%) influence on the measured CCS values for three repeat measurements (**Figure S1**). The role of methanol is presumed to be in enhancing solubility and ionization of analytes in general, rather than affecting the formation or survival of specific complexes since the unbound (M and βCD) ions are also enhanced by its presence. A 3:2 methanol:water composition was used for the final analytical dilution of the samples in all subsequent work described here.

Various guest:host solution-phase ratios (1:5, 1:1, and 5:1) were also evaluated for three select systems (CIN, CHL, and RUT with βCD) and as noted from other MS studies on guest:host complexes,(Guo *et al.*, 2004; Yu *et al.*, 2007; Zlibut *et al.*, 2023) the solution phase ratio of guest and host did not significantly affect the relative abundances of the various complexes observed in the mass spectra (**Figure S2**). Prior host-guest results have indicated that the gas-phase relative abundances observed in ESI reflect the solution-phase binding affinities of each complex,(Lee *et al.*, 2013) thus the approximately equal abundances yielded in this work suggests that these are kinetically-favored complexes which are relatively insensitive to small changes in the reactant stoichiometries. A slight (~25%) enhancement in signal is observed once the guest is presented in equal or greater amounts to the host (1:1 and 5:1 guest:host ratios), thus a 1:1 guest-host ratio was used for all subsequent work. The instrument parameters previously optimized for the artemisinin-βCD system were used with no additional optimization (**Table S2**).(Zlibut *et al.*, 2023)

## Appendix 3 – Processing ERMS Data

Precursor abundances at each CE were converted to a precursor depletion ratio between 0 and 1 using equation 1:

$$Precursor\ Depletion = \frac{I_0 - I_{CE}}{I_0} \qquad \textbf{equation 1}$$

$I_0$ is the intensity of the precursor ion at 0 V, and $I_{CE}$ is the precursor intensity at the given CID voltage. Laboratory frame voltages were converted to center-of-mass collision energies ($E_{COM}$) and precursor depletion ratios were plotted as a function of $E_{COM}$ to generate ion depletion curves.

## Appendix 4 – Details of the Computational Modeling

Unbound host and guest are DFT-optimized, and harmonic distance restraints of 8 Å from the center of the βCD ring were used to anchor the guest molecule to the host. This serves to maximize guest:host interaction throughout the production time and allowed for a thorough sampling of favorable guest inclusion orientations. The 8 Å restriction is the approximate dimensions of the βCD host molecule (height 7.9 Å, radius of periphery 7.8 Å),(Bekers *et al.*, 1991) and thus was chosen to allow both inclusion and non-inclusion interactions to be sampled. GAFF parameters were used in AMBER for both βCD and the small molecule guests (**Table S5**). Atom assignments were automated using the Antechamber software tool. Structures are clustered by similarity using root mean square distance (RMSD) analysis, and each conformational family is visually assessed based on whether the guest is included within the βCD cavity.

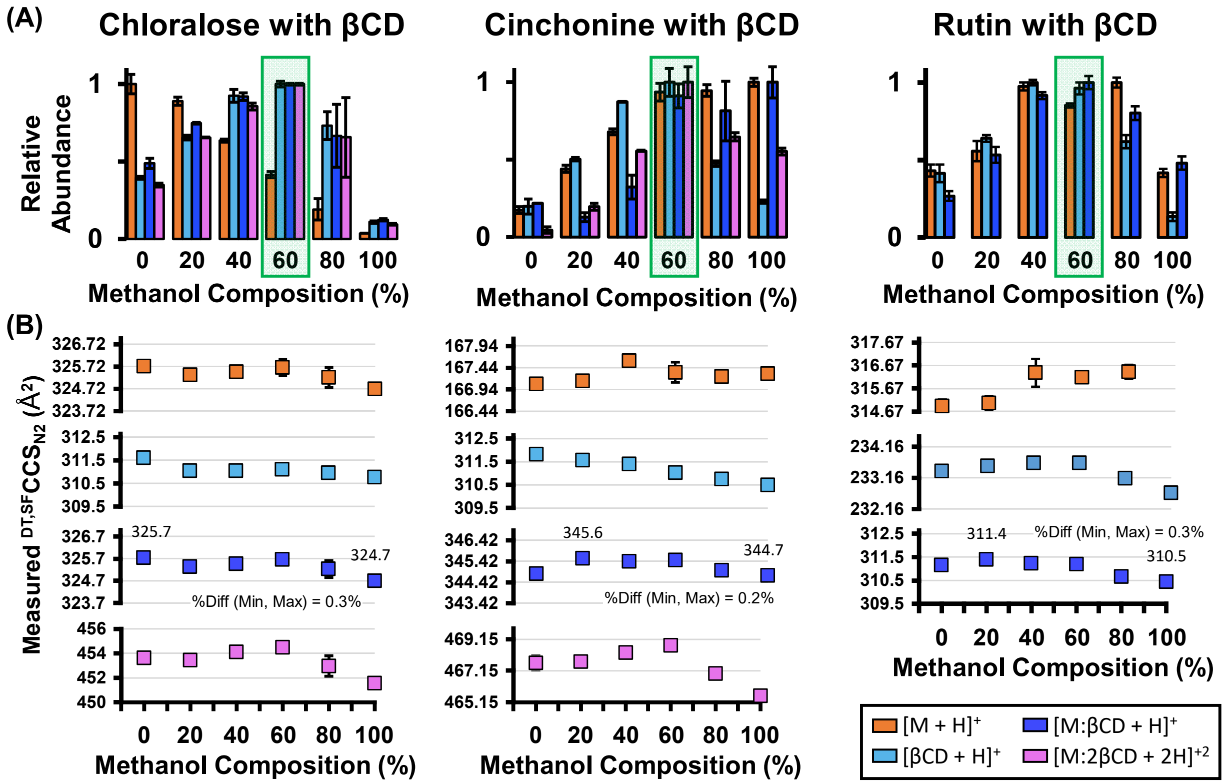


**Figure S1.** Results from varying the methanol composition of three small molecule:βCD samples (10 μM). **(A)** Relative abundances of both unbound and complexed ions were observed to be the highest for a 60% methanol sample solution in all three examples, and **(B)** the measured collision cross section (CCS), while statistically different in the different methanol compositions, was found to vary by ca. 0.3% or less. In most cases, the highest CCS was observed at the intermediate (40 & 60%) methanol compositions. ART:βCD (data not shown) was also evaluated with similar abundance and CCS results. For all data, results are averaged over triplicate measurements. For panel (B), the y-axes are scaled to ±0.5% of the average CCS value to facilitate direct comparisons across the scatter plots. The highest and lowest CCS values and the corresponding percent differences (%Diff) are annotated for the $[M:\beta CD+H]^+$ ion complex data (blue). "$^{DT,SF}CCS_{N2}$" corresponds to drift tube CCS measurements obtained in nitrogen drift gas using the single-field calibration method.(May *et al.*, 2017; Stow *et al.*, 2017)

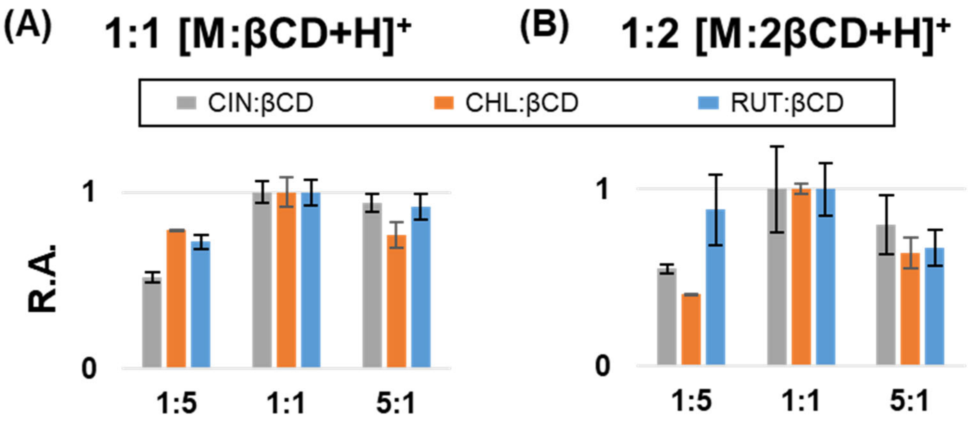


**Figure S2.** Evaluation of three solution-phase guest:host ratios for three guest:host systems (CIN=cinchonine; CHL=chloralose, RUT=rutin) and the effect of these ratios on the relative abundance (R.A.) of **(A)** the protonated 1:1 complex, $[M:\beta CD+H]^+$, and **(B)** the protonated 1:2 complex, $[M:\beta CD+H]^+$. Here the relative abundances are scaled to the maximum ion signal observed across the three ratios for each system.

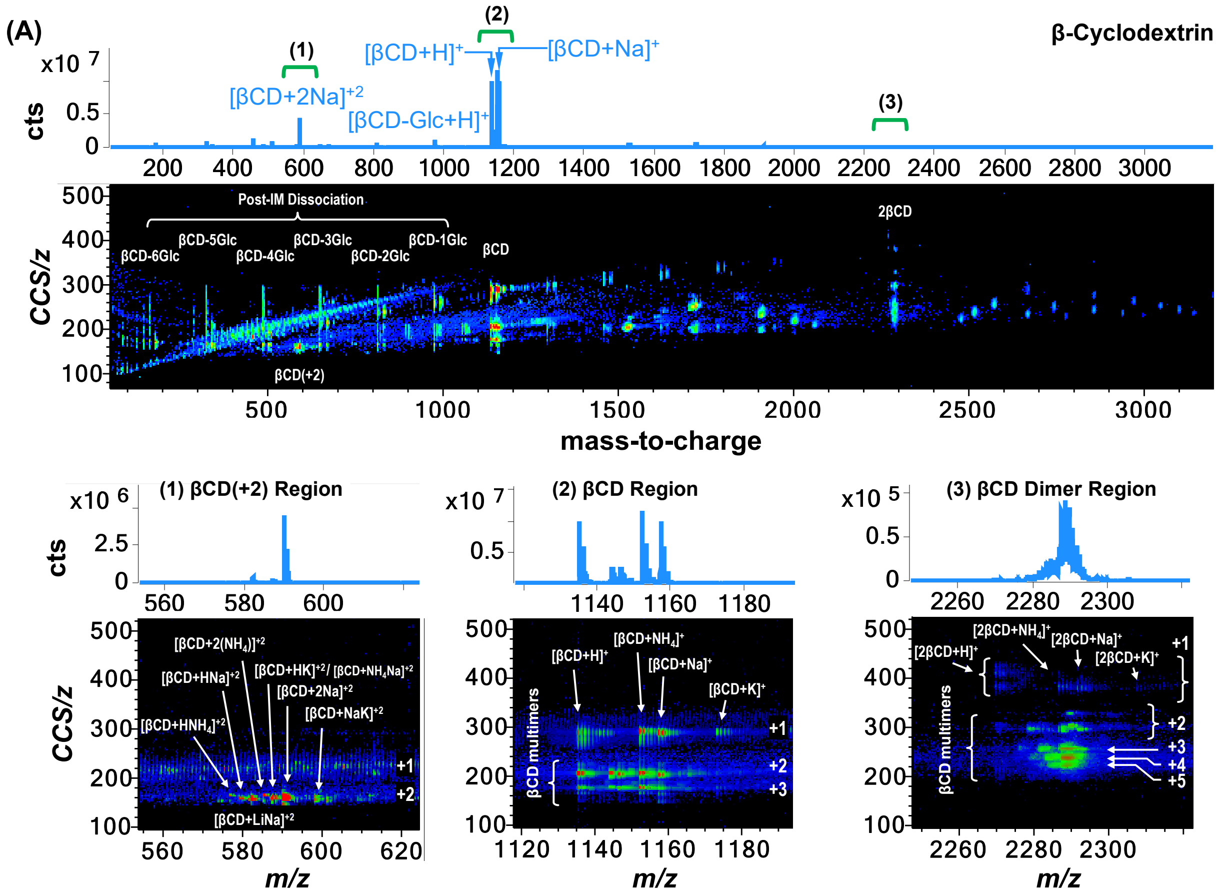


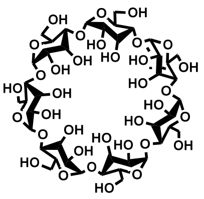

β-Cyclodextrin
$C_{42}H_{70}O_{35}$
Neutral Mass = 1134.3697 Da
$[M+H]^+$ = *m/z* 1135.376
$[M+Na]^+$ = *m/z* 1157.3595

**Figure S3. (A)** IM-MS spectrum of β-cyclodextrin (100 µM in 3:2 methanol:water) with specific regions of interest expanded to illustrate the complexity of the host matrix. **(1)** Region containing doubly-charged βCD and isobars, **(2)** region containing singly-charged βCD and isobaric multiply-charged multimers, and **(3)** region containing singly-charged βCD dimer and higher order multiply-charged multimers that appear at lower charge-specific regions of the IM-MS projections. The various signals that appear between these three regions correspond to multimer ions with fractional *m/z* values. Signals appearing at low *m/z* but span across a broad range of CCS (i.e., "streaking") are ion dissociation artifacts with characteristic losses of glucose (Glc) sub-units, arising from βCD dissociation during and after the IM analysis.(Zlibut *et al.*, 2023)

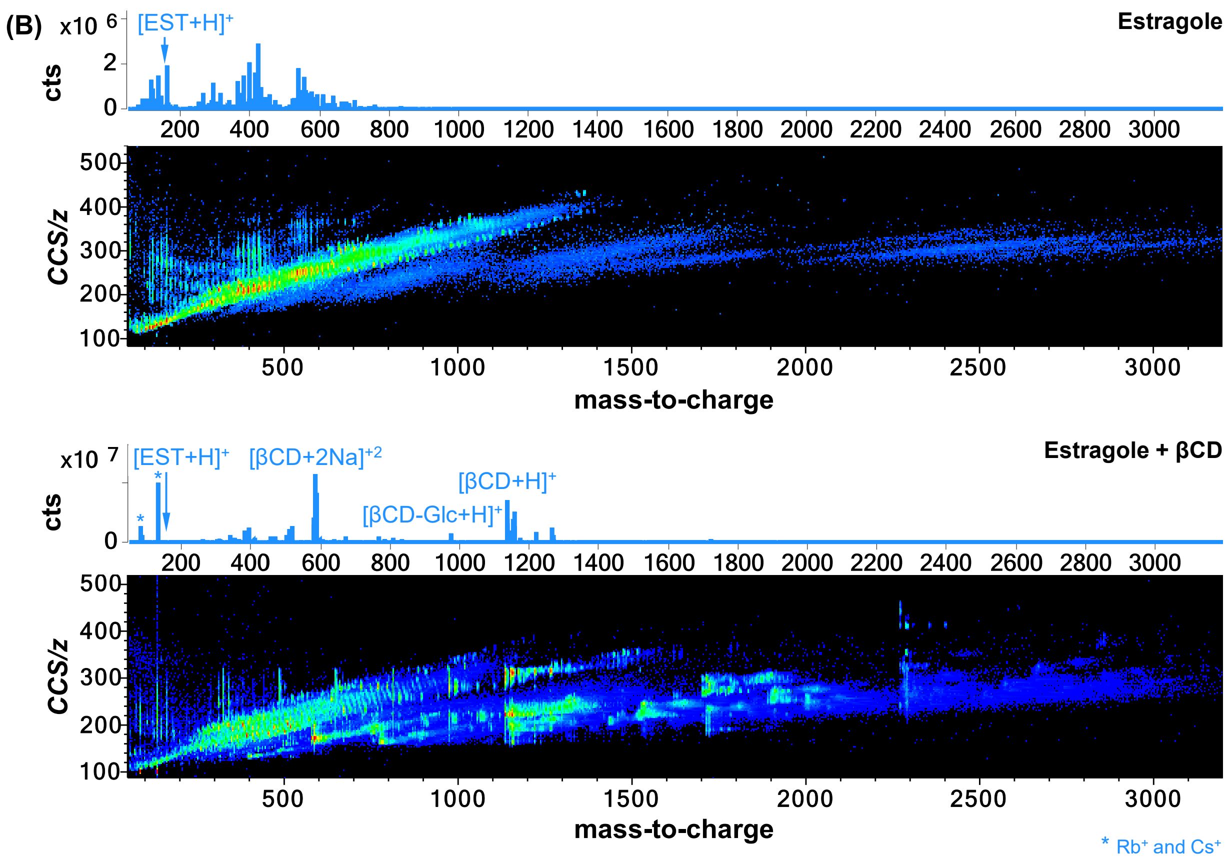


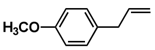


Estragole
$C_{10}H_{12}O$
Neutral Mass = 148.0888 Da
$[M+H]^+$ = *m/z* 149.0966

**Figure S3. (B)** IM-MS spectrum of **(top)** estragole (10 µM in 3:2 methanol:water) and **(bottom)** estragole with β-cyclodextrin (1:1 molar ratio).

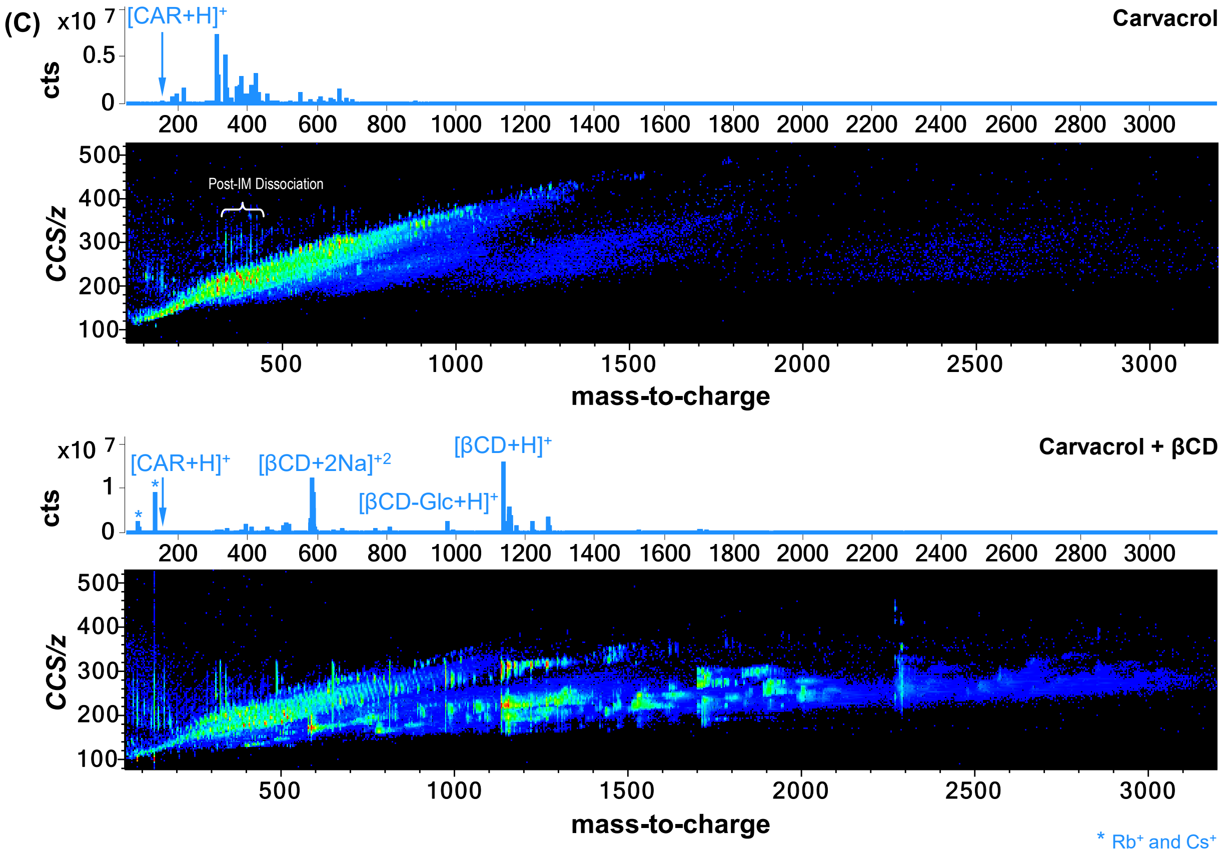


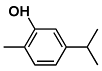


Carvacrol
$C_{10}H_{14}O$
Neutral Mass = 150.1045 Da
$[M+H]^+$ = *m/z* 151.1123

**Figure S3. (C)** IM-MS spectrum of **(top)** carvacrol (10 µM in 3:2 methanol:water) and **(bottom)** carvacrol with β-cyclodextrin (1:1 molar ratio).

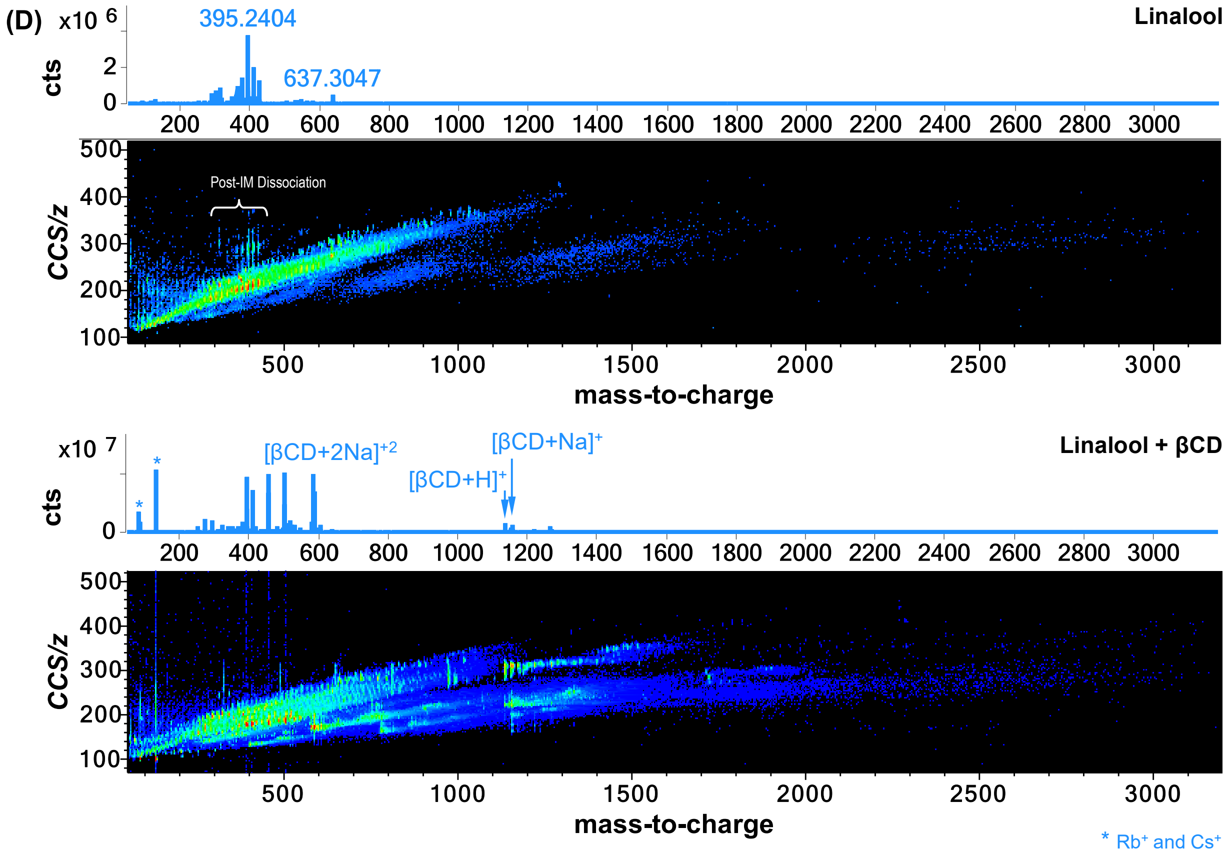


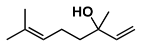


Linalool
$C_{10}H_{18}O$
Neutral Mass = 154.1358 Da
$[M+H]^+$ = *m/z* 155.1436

**Figure S3. (D)** IM-MS spectrum of **(top)** linalool (10 μM in 3:2 methanol:water) and **(bottom)** linalool with β-cyclodextrin (1:1 molar ratio).

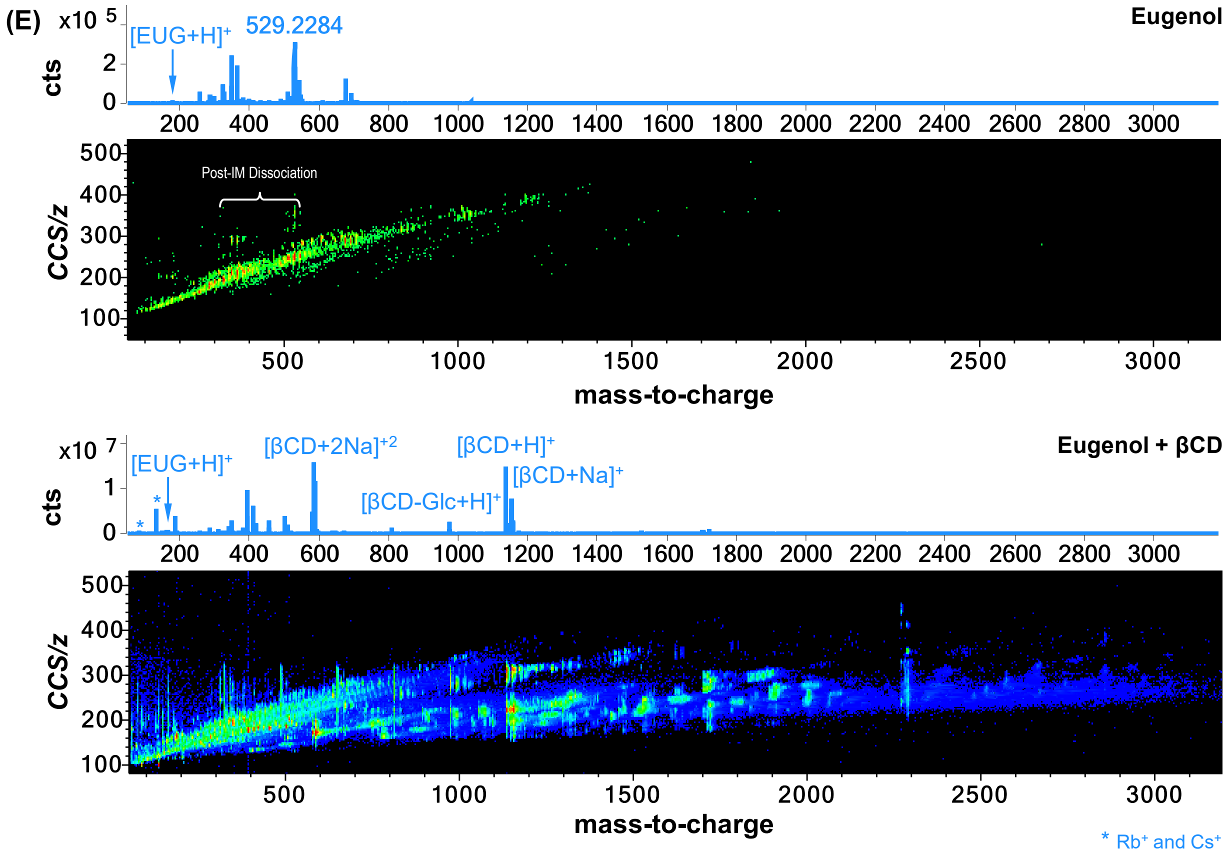


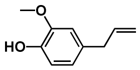


Eugenol
$C_{10}H_{12}O_2$
Neutral Mass = 164.0837 Da
$[M+H]^+$ = *m/z* 165.0915

**Figure S3. (E)** IM-MS spectrum of **(top)** eugenol (10 µM in 3:2 methanol:water) and **(bottom)** eugenol with β-cyclodextrin (1:1 molar ratio).

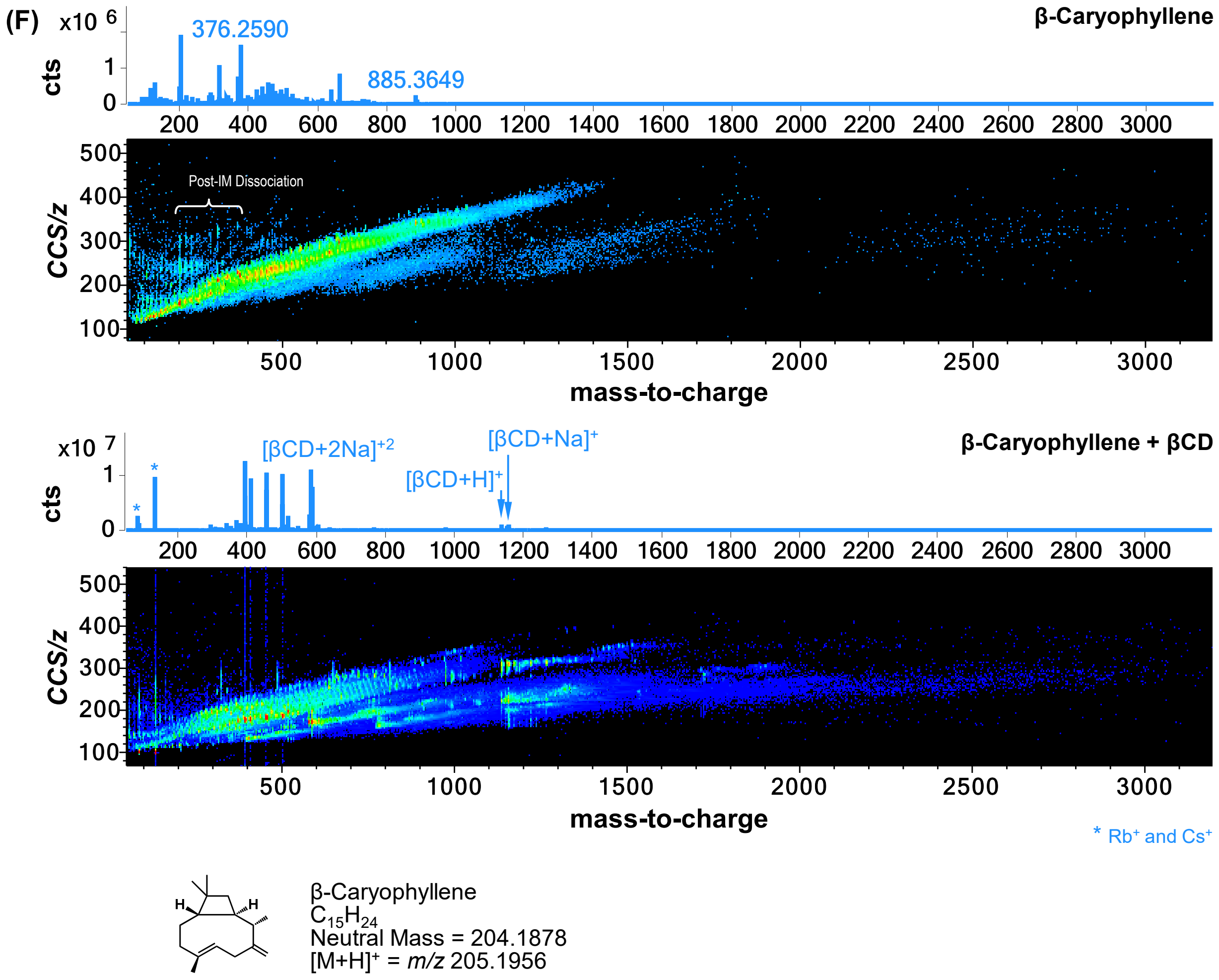


**Figure S3. (F)** IM-MS spectrum of **(top)** β-caryophyllene (10 μM in 3:2 methanol:water) and **(bottom)** β-caryophyllene with β-cyclodextrin (1:1 molar ratio).

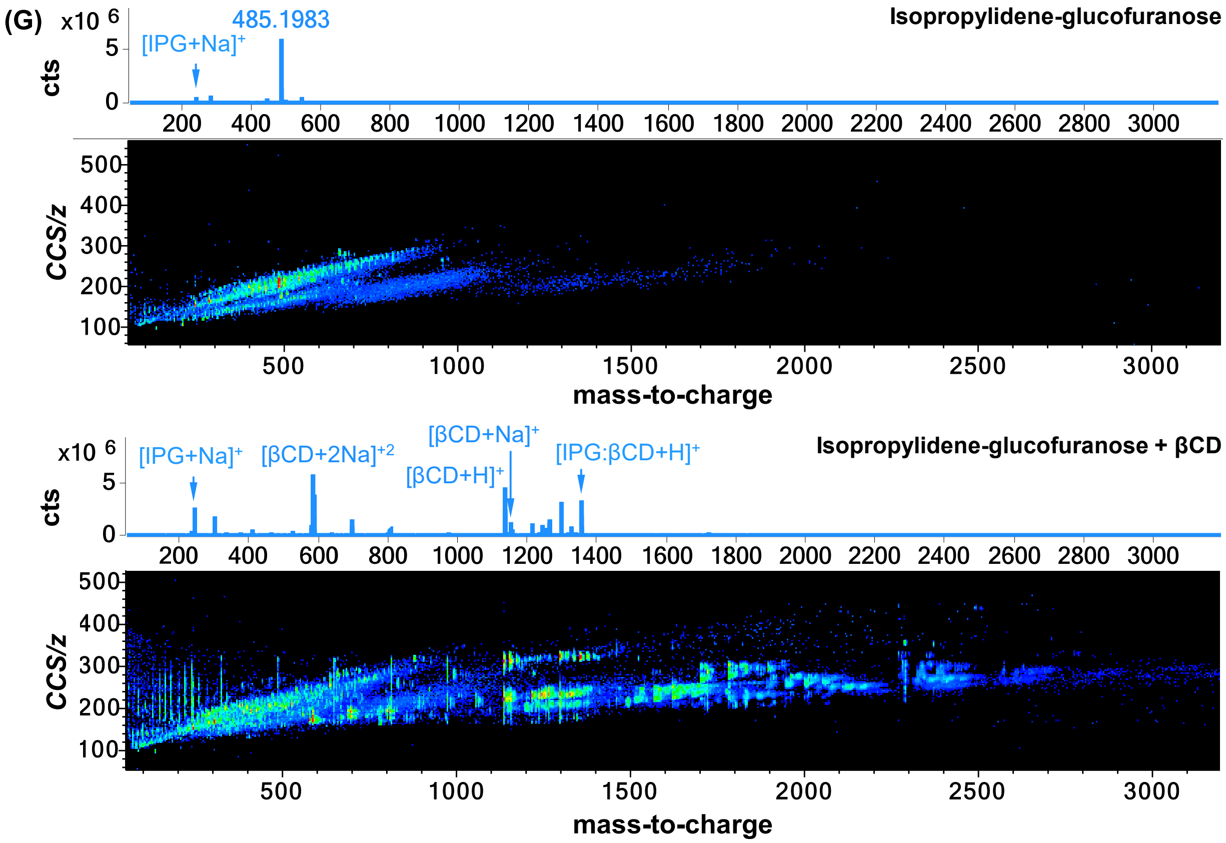


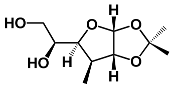

1,2-O-isopropylidene-α-D-glucofuranose
$C_9H_{16}O_6$
Neutral Mass = 220.0947 Da
$[M+H]^+$ = *m/z* 221.1025
$[M+Na]^+$ = *m/z* 243.0845

**Figure S3. (G)** IM-MS spectrum of **(top)** 1,2-O-isopropylidene-α-D-glucofuranose (10 μM in 3:2 methanol:water) and **(bottom)** 1,2-O-isopropylidene-α-D-glucofuranose with β-cyclodextrin (1:1 molar ratio).

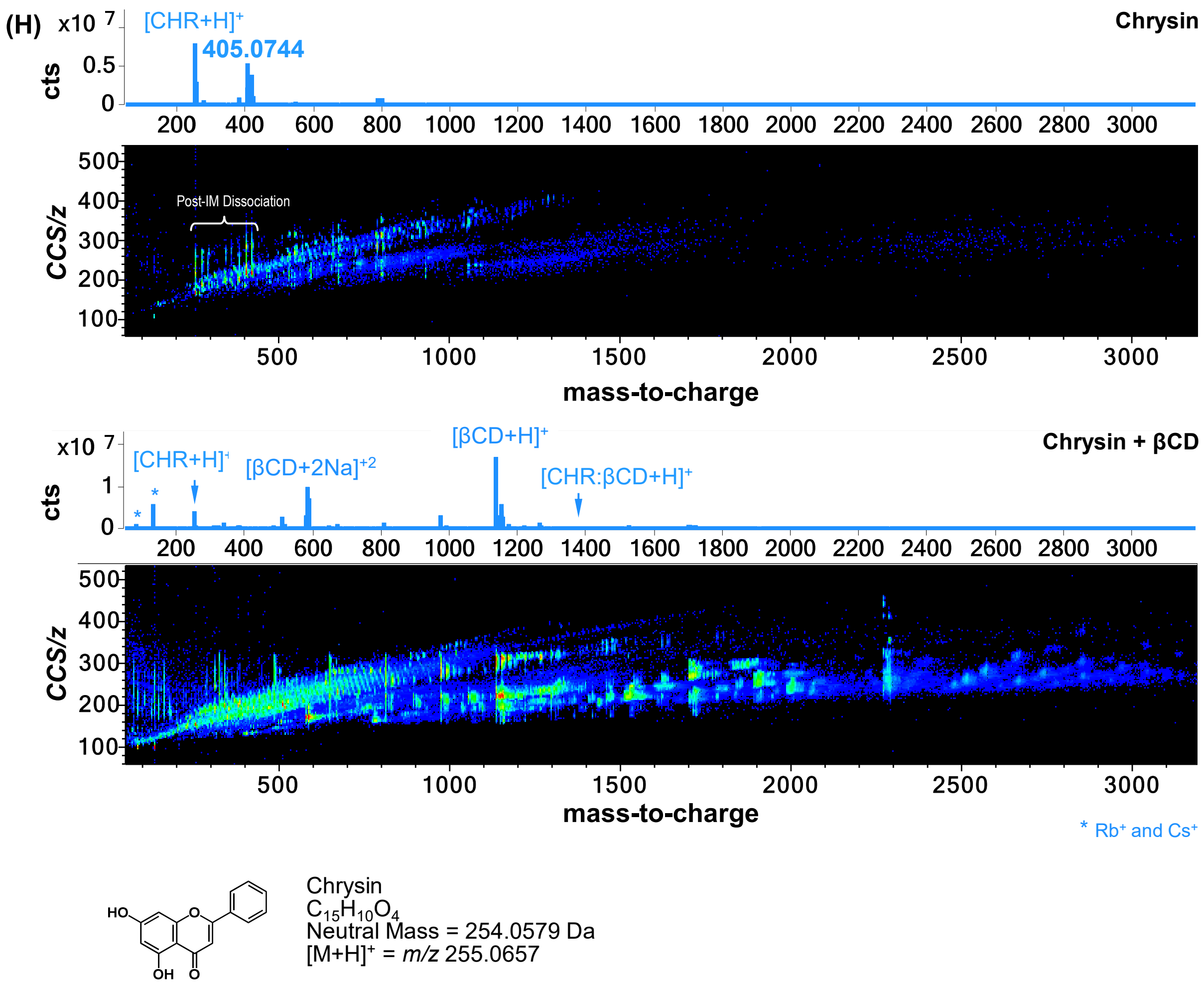


**Figure S3. (H)** IM-MS spectrum of **(top)** chrysin (10 μM in 3:2 methanol:water) and **(bottom)** chrysin with β-cyclodextrin (1:1 molar ratio).

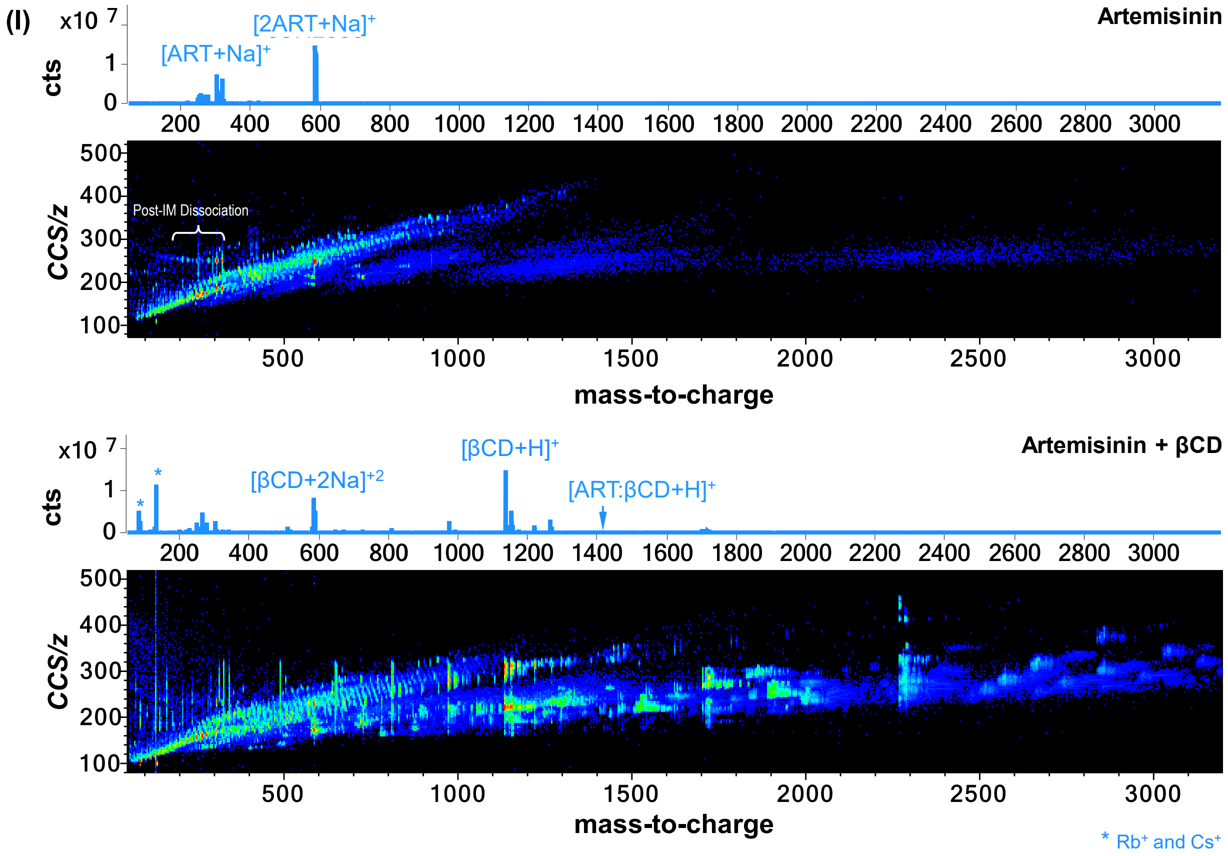


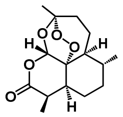

Artemisinin
$C_{15}H_{22}O_5$
Neutral Mass = 282.1467 Da
$[M+H]^+$ = *m/z* 283.1545

**Figure S3. (I)** IM-MS spectrum of **(top)** artemisinin (10 µM in 3:2 methanol:water) and **(bottom)** artemisinin with β-cyclodextrin (1:1 molar ratio).

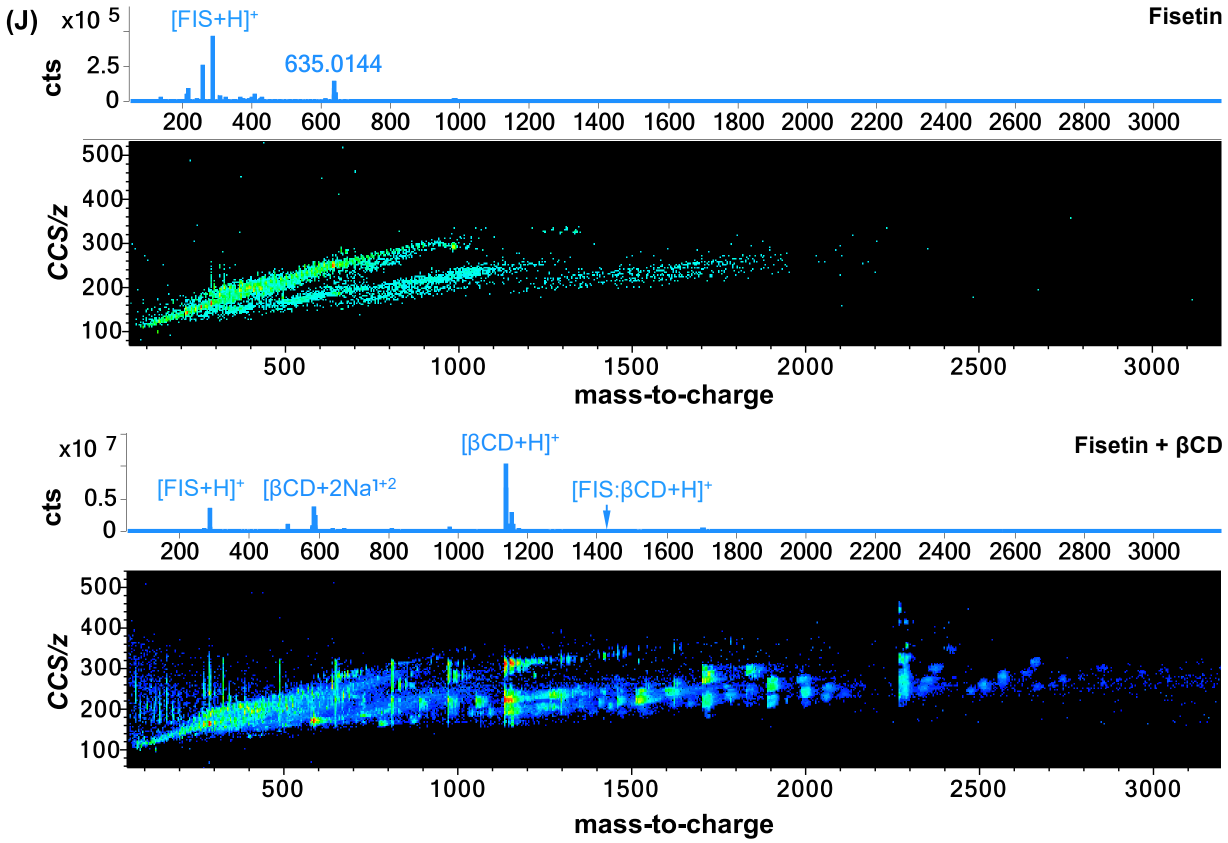


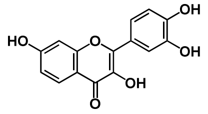


Fisetin
$C_{15}H_{10}O_6$
Neutral Mass = 286.0477 Da
$[M+H]^+$ = *m/z* 287.0556

**Figure S3. (J)** IM-MS spectrum of **(top)** fisetin (10 µM in 3:2 methanol:water) and **(bottom)** fisetin with β-cyclodextrin (1:1 molar ratio).

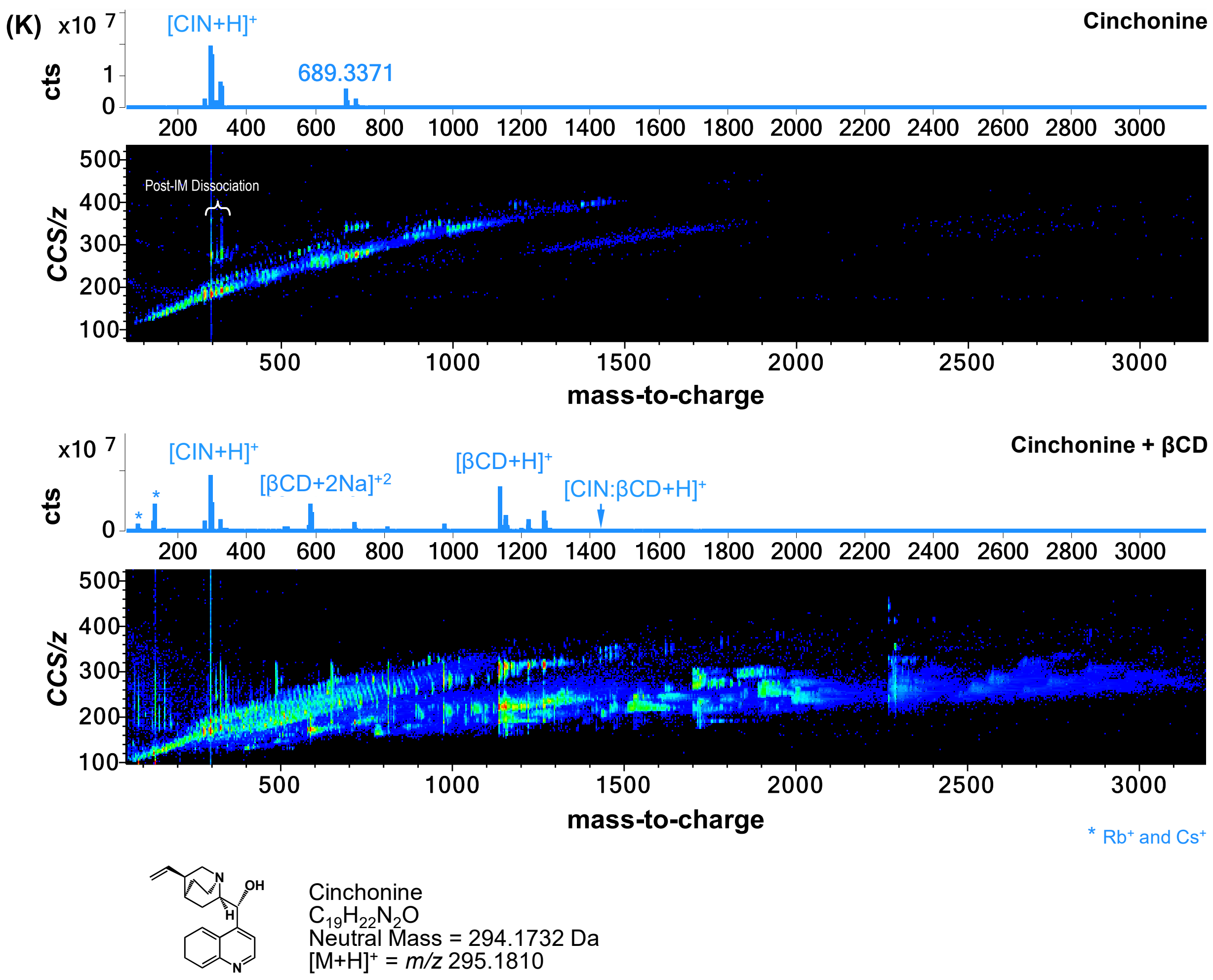


**Figure S3. (K)** IM-MS spectrum of **(top)** cinchonine (10 µM in 3:2 methanol:water) and **(bottom)** cinchonine with β-cyclodextrin (1:1 molar ratio).

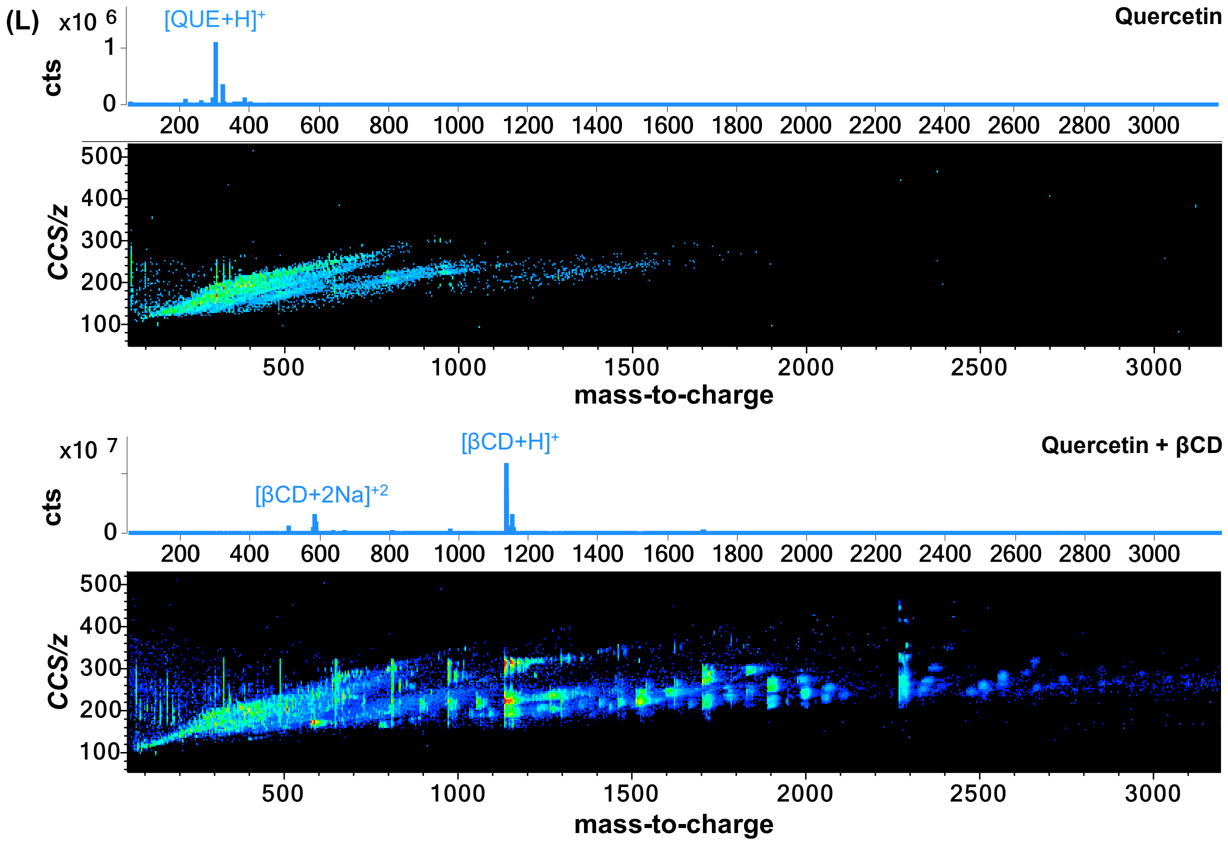


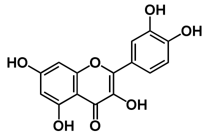


Quercetin
$C_{15}H_{10}O_7$
Neutral Mass = 302.0427 Da
$[M+H]^+$ = *m/z* 303.0505

**Figure S3. (L)** IM-MS spectrum of **(top)** quercetin (10 µM in 3:2 methanol:water) and **(bottom)** quercetin with β-cyclodextrin (1:1 molar ratio).

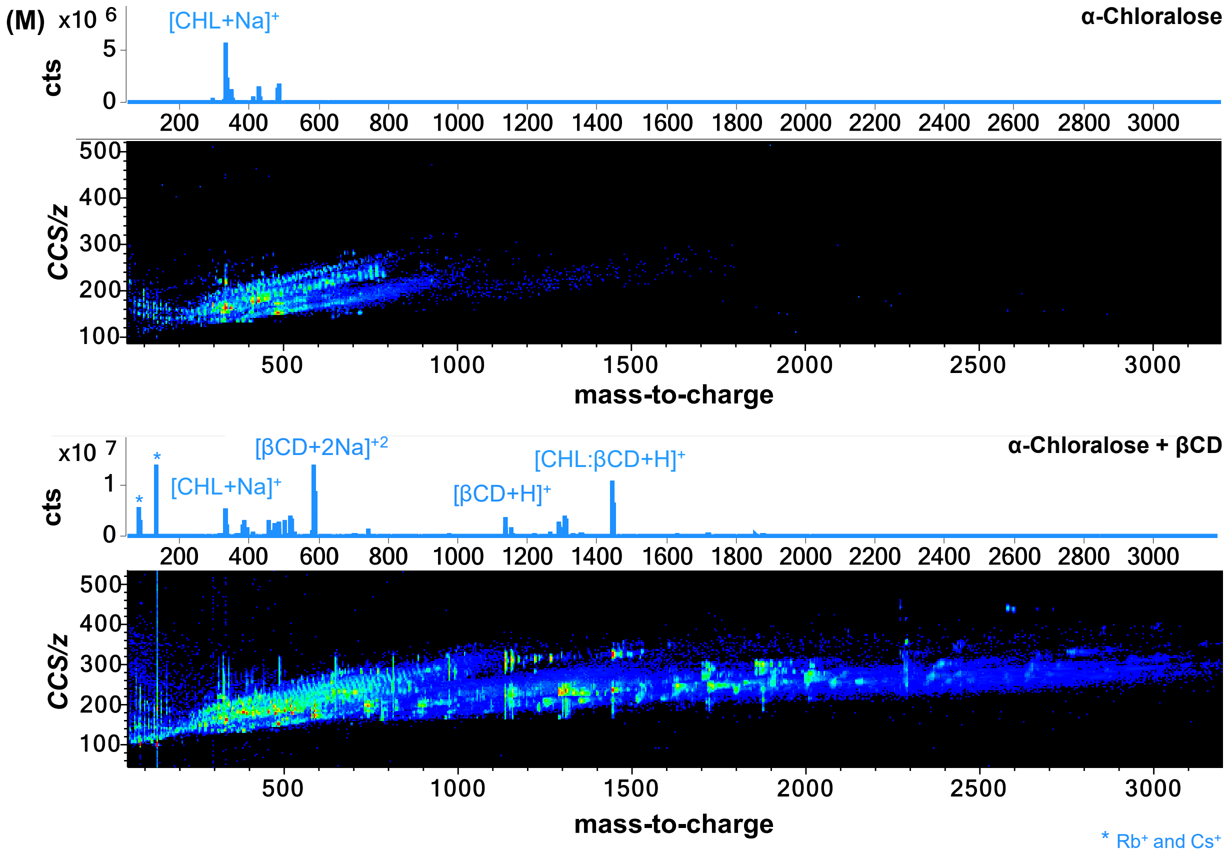


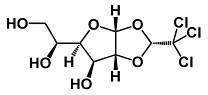

α-Chloralose
$C_8H_{11}Cl_3O_6$
Neutral Mass = 307.9621 Da
$[M+H]^+$ = *m/z* 308.9699
$[M+Na]^+$ = *m/z* 330.9519

**Figure S3. (M)** IM-MS spectrum of **(top)** α-chloralose (10 μM in 3:2 methanol:water) and **(bottom)** α-chloralose with β-cyclodextrin (1:1 molar ratio).

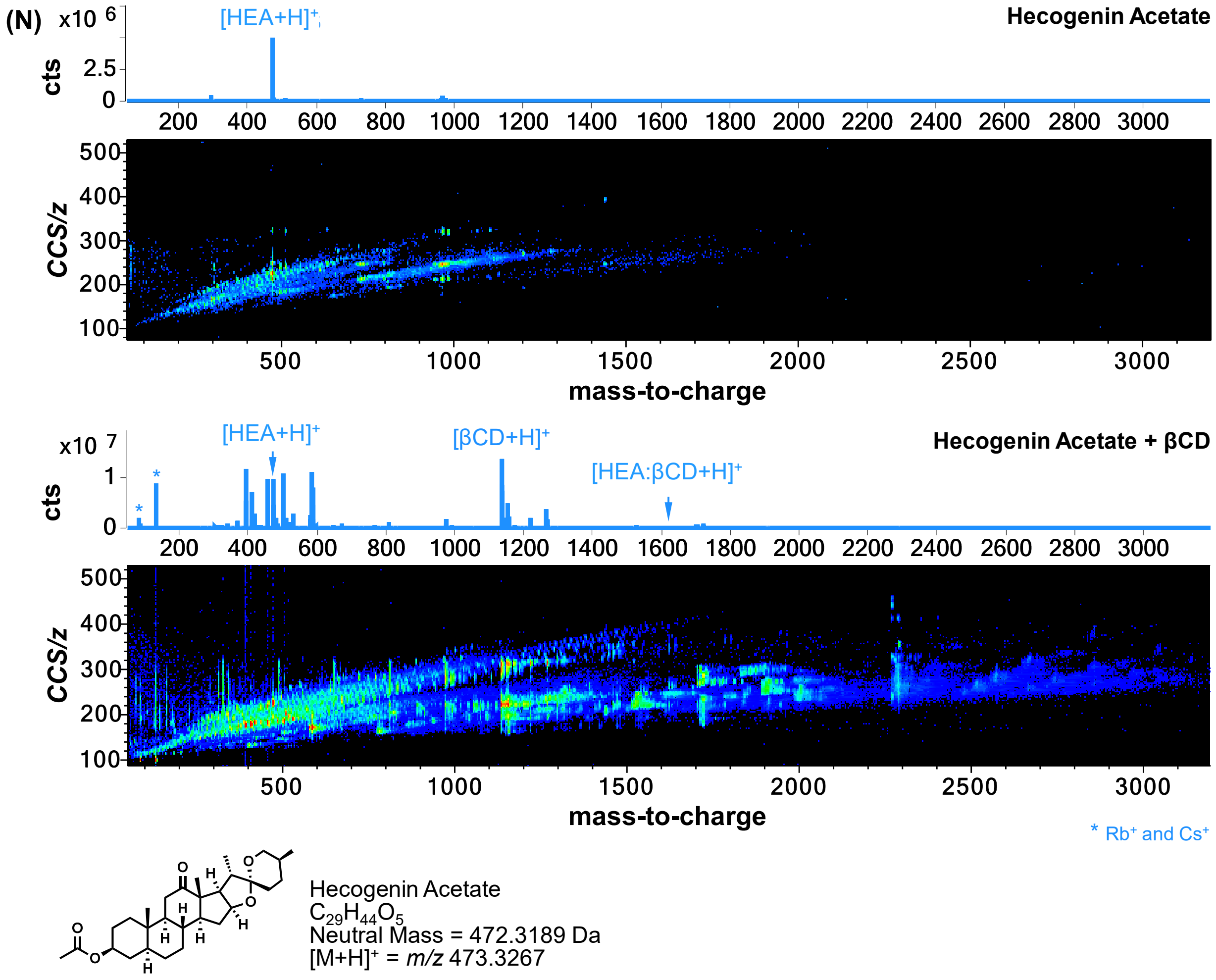


**Figure S3. (N)** IM-MS spectrum of **(top)** hecogenin acetate (10 μM in 3:2 methanol:water) and **(bottom)** hecogenin acetate with β-cyclodextrin (1:1 molar ratio).

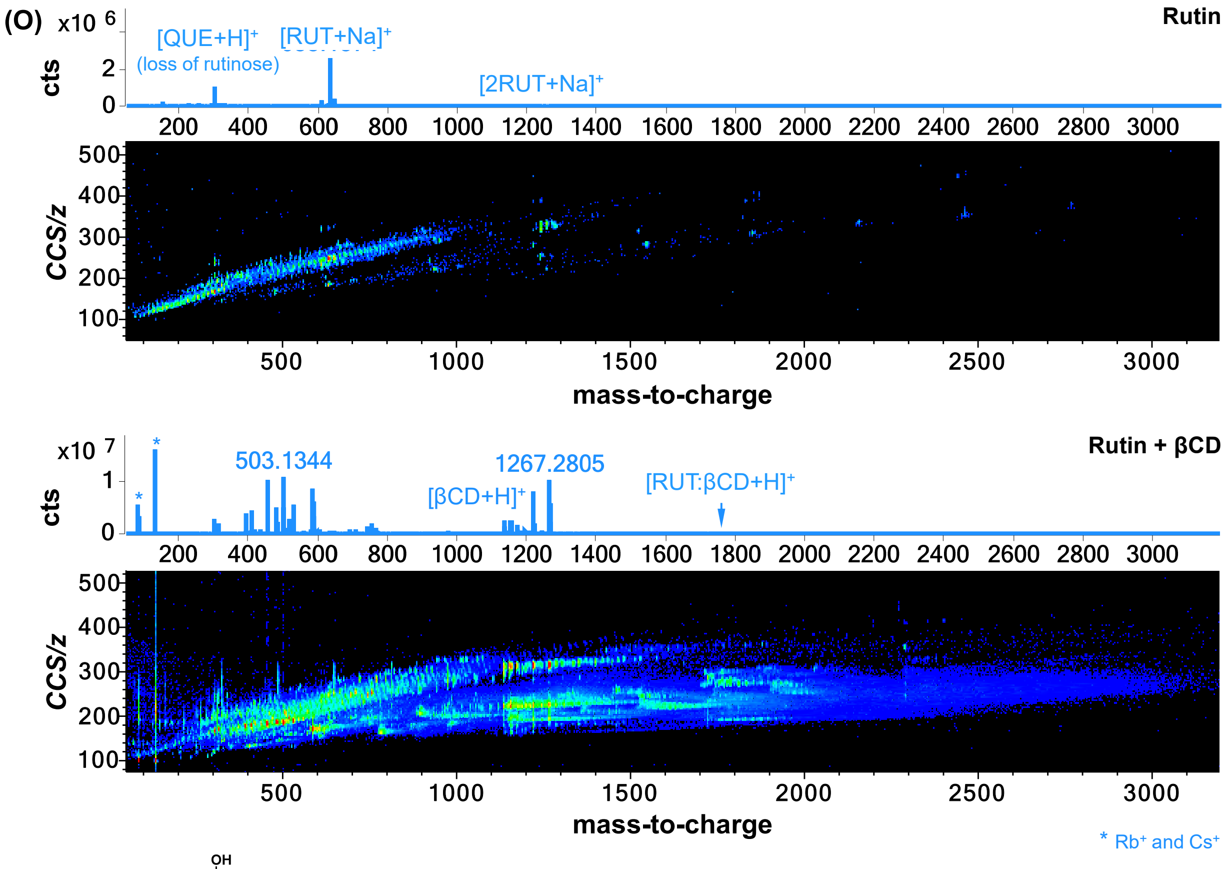


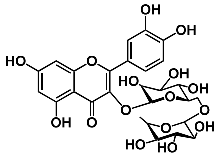

Rutin
$C_{27}H_{30}O_{16}$
Neutral Mass = 610.1534 Da
$[M+H]^+$ = *m/z* 611.1612
$[M+Na]^+$ = *m/z* 633.1431

**Figure S3. (O)** IM-MS spectrum of **(top)** rutin (10 μM in 3:2 methanol:water) and **(bottom)** rutin with β-cyclodextrin (1:1 molar ratio).

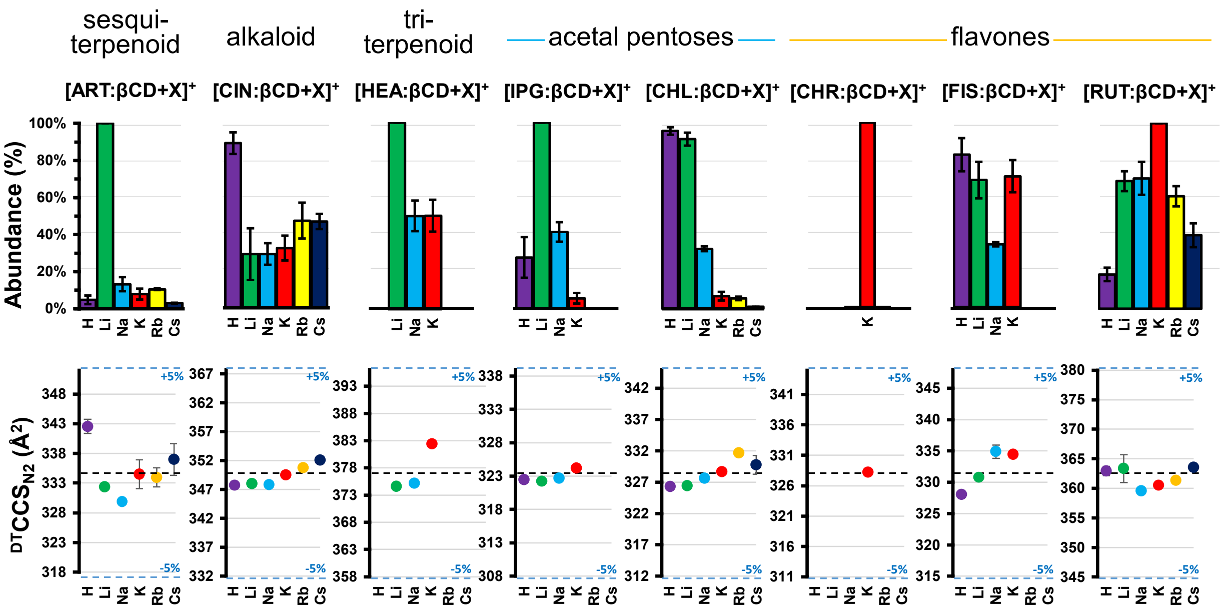


**Figure S4.** Positive mode ion complex results from cation competition experiments where an equimolar (10 μM) mixture of five group 1a alkali metal salts (LiOAc, NaOAc, KOAc, RbOAc, and CsOAc) was added 1:1 to each small molecule:βCD mixture. Observed relative abundances are summarized in the top panel, and corresponding CCS measurements are shown in the lower panel. The CCS axes are centered to the average CCS across all cations and scaled to ±5% to facilitate direct comparisons. Error bars correspond to standard deviations from triplicate, inter-day measurements.

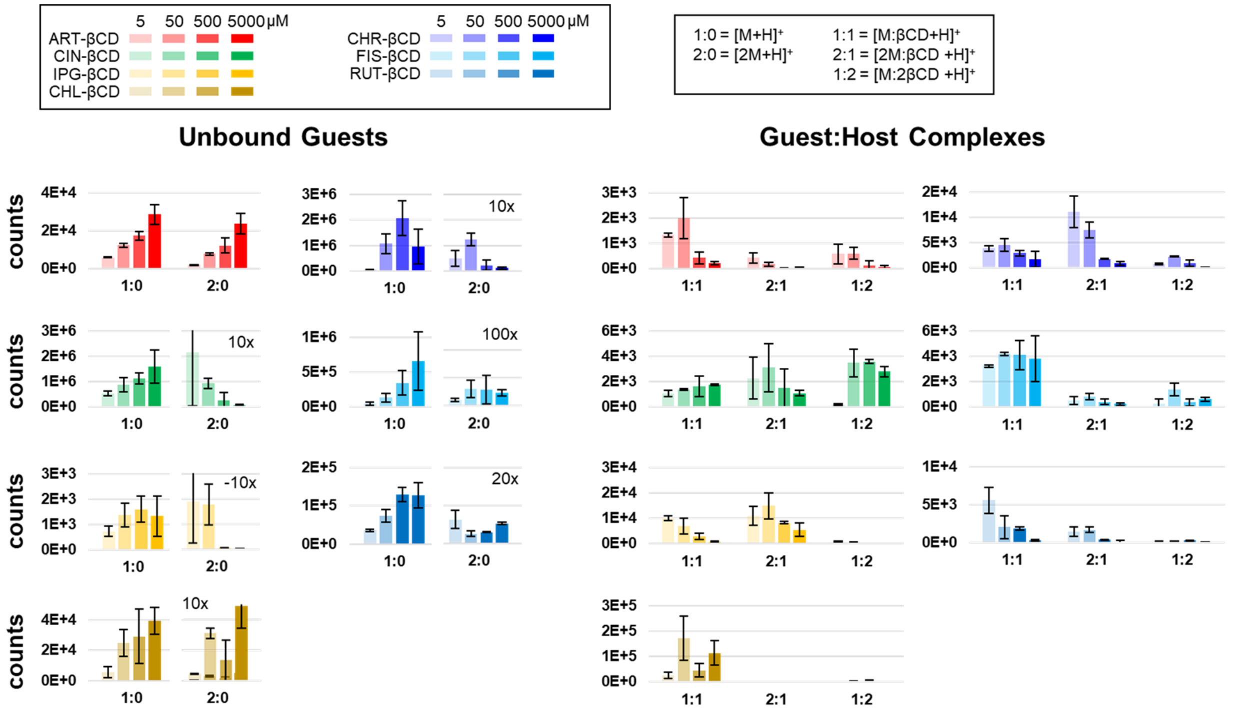


**Figure S5.** Ion abundance results (counts) obtained from sample dilution tests conducted on select sample mixtures. Four sample concentrations (5, 50, 500, and 5000 μM) were evaluated for seven samples (ART, CIN, IPG, CHL, CHR, FIS and RUT with BCD). Results for unbound guest molecule monomers and dimers (first column) in most cases show an increase in signal as the sample concentration is increased. In contrast, for guest:host complexes (right column), the signal either remains consistent or decreases as the sample concentration increases, which suggest these ion signals represent specific guest:host binding and do not originate from nonspecific aggregation.

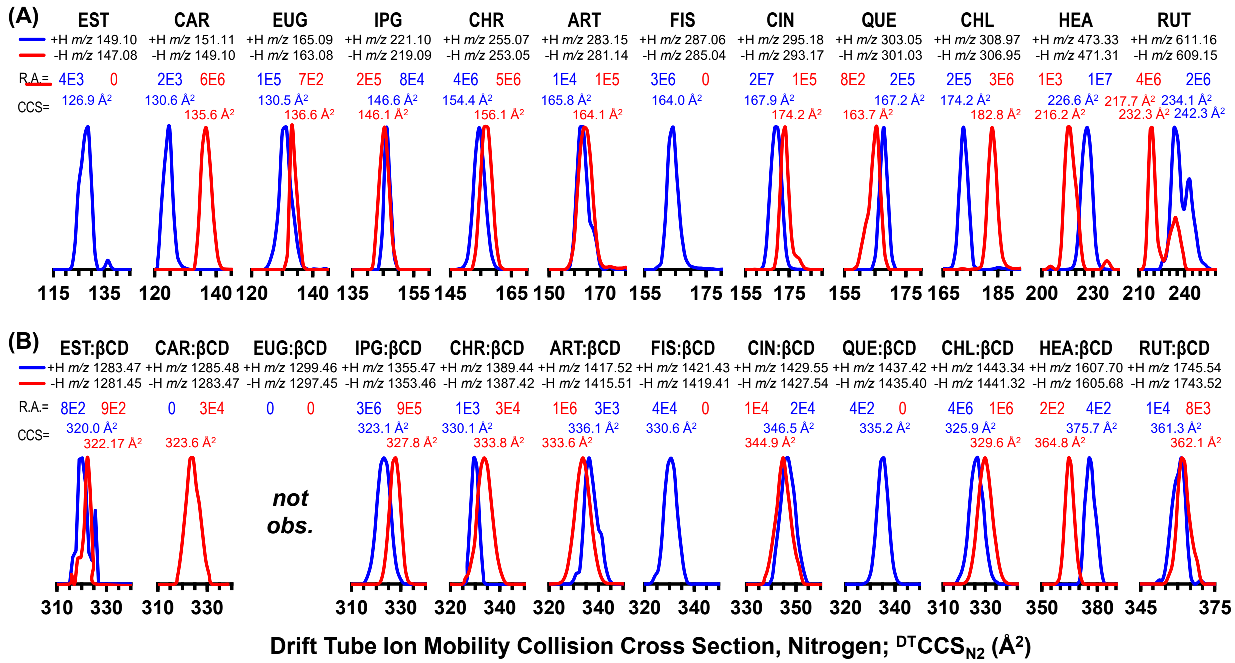


**Figure S6.** Ion mobility profiles for **(A)** the unbound small molecules, and **(B)** the small molecule:βCD 1:1 complexes. Both protonated (blue) and deprotonated (red) ion forms are shown. In each panel, the profiles are ordered, from left to right, by increasing mass. The magnitudes of the peak area relative abundances (R.A.) and collision cross section (CCS) values are provided above each profile, and CCS axes are all scaled to *ca.* ±8% to facilitate comparisons across the mobility range surveyed.

**Table S3.** Collision cross section measurements in nitrogen obtained for protonated (top) and deprotonated (bottom) ions.

| Nitrogen CCS Measurements | | | Protonated Ions | | | | | | | | | | | | | | | | |
|---|---|---|---|---|---|---|---|---|---|---|---|---|---|---|---|---|---|---|
| | | | unbound | | | | 1:1 complexes | | | | 1:2 complexes | | | | 2:1 complexes | | | |
| Analyte | Neutral Formula | Neutral Mass | [M+H]+ | | | | [M+βCD+H]+ | | | | [M+2βCD+H]+ | | | | [2M+βCD+H]+ | | | |
| | | | CCS | SD | %RSD | n | CCS | SD | %RSD | n | CCS | SD | %RSD | n | CCS | SD | %RSD | n |
| EST | C10H12O | 148.09 | 126.92 | 0.6 | 0.5% | 4 | 320.00 | 18.5 | 5.8% | 3 | 441.69 | 1.2 | 0.3% | 4 | -- | -- | -- | - |
| CAR | C10H14O | 150.10 | 130.56 | 1.2 | 0.9% | 4 | -- | -- | -- | - | -- | -- | -- | - | -- | -- | -- | - |
| LIN | C10H18O | 154.14 | -- | -- | -- | - | -- | -- | -- | - | -- | -- | -- | - | -- | -- | -- | - |
| EUG | C10H12O2 | 164.08 | 130.52 | 0.4 | 0.3% | 4 | -- | -- | -- | - | -- | -- | -- | - | -- | -- | -- | - |
| CPL | C15H24 | 204.19 | -- | -- | -- | - | -- | -- | -- | - | -- | -- | -- | - | -- | -- | -- | - |
| ART | C15H22O5 | 282.15 | 165.81 | 2.0 | 1.2% | 4 | 336.09 | 2.6 | 0.8% | 4 | 444.08 | 3.1 | 0.7% | 4 | -- | -- | -- | - |
| CIN | C19H22N2O | 294.17 | 167.91 | 0.3 | 0.2% | 4 | 346.46 | 1.5 | 0.4% | 4 | 443.01 | 3.2 | 0.7% | 4 | -- | -- | -- | - |
| HEA | C29H44O5 | 472.32 | 226.60 | 0.2 | 0.1% | 4 | 375.72 | 1.5 | 0.4% | 4 | 460.06 | 1.1 | 0.2% | 4 | -- | -- | -- | - |
| IPG | C9H16O6 | 220.09 | 146.57 | 2.2 | 1.5% | 4 | 323.09 | 0.2 | 0.1% | 4 | 439.69 | 0.7 | 0.2% | 4 | 349.22 | 4.3 | 1.2% | 4 |
| CHL | C8H11Cl3O6 | 307.96 | 174.24 | 2.8 | 1.6% | 4 | 325.85 | 0.3 | 0.1% | 4 | 440.99 | 0.5 | 0.1% | 3 | 367.88 | 1.3 | 0.3% | 3 |
| CHR | C15H10O4 | 254.06 | 154.37 | 0.2 | 0.1% | 4 | 330.07 | 0.3 | 0.1% | 4 | 447.49 | 2.8 | 0.6% | 4 | 352.07 | 1.6 | 0.5% | 4 |
| FIS | C15H10O6 | 286.05 | 163.98 | 0.3 | 0.2% | 4 | 330.57 | 0.2 | 0.1% | 4 | 441.56 | 2.6 | 0.6% | 4 | 360.18 | 1.5 | 0.4% | 4 |
| QUE | C15H10O7 | 302.04 | 167.21 | 0.1 | 0.1% | 4 | 335.16 | 1.8 | 0.5% | 4 | 438.31 | 1.1 | 0.2% | 3 | -- | -- | -- | - |
| RUT | C27H30O16 | 610.15 | 234.07 | 0.5 | 0.2% | 4 | 361.27 | 0.5 | 0.1% | 4 | 443.11 | 4.6 | 1.0% | 2 | -- | -- | -- | - |
| | | peak 2= | 242.31 | 0.6 | 0.2% | 3 | | | | | | | | | | | | |
| | | | | | | | | | | | | | | | | | | |
| | | | [βCD + H]+ | | | | [2βCD + H]+ | | | | | | | | | | | |
| βCD | C42H70O35 | 1134.37 | 298.57 | 0.6 | 0.2% | 3 | 443.10 | 0.4 | 0.1% | 3 | | | | | | | | |
| | | peak 2= | 311.48 | 0.9 | 0.3% | 3 | 456.97 | 0.9 | 0.2% | 3 | | | | | | | | |

| Nitrogen CCS Measurements | | | Deprotonated Ions | | | | | | | | | | | | | | | | |
|---|---|---|---|---|---|---|---|---|---|---|---|---|---|---|---|---|---|---|
| | | | unbound | | | | 1:1 complexes | | | | 1:2 complexes | | | | 2:1 complexes | | | |
| Analyte | Neutral Formula | Neutral Mass | [M-H]- | | | | [M+βCD-H]- | | | | [M+2βCD-H]- | | | | [2M+βCD-H]- | | | |
| | | | CCS | SD | %RSD | n | CCS | SD | %RSD | n | CCS | SD | %RSD | n | CCS | SD | %RSD | n |
| EST | C10H12O | 148.09 | -- | -- | -- | - | 322.17 | 1.1 | 0.3% | 3 | -- | -- | -- | - | -- | -- | -- | - |
| CAR | C10H14O | 150.10 | 135.59 | 1.2 | 0.9% | 4 | 323.63 | 3.4 | 1.0% | 4 | -- | -- | -- | - | -- | -- | -- | - |
| LIN | C10H18O | 154.14 | -- | -- | -- | - | -- | -- | -- | - | -- | -- | -- | - | -- | -- | -- | - |
| EUG | C10H12O2 | 164.08 | 136.64 | 2.5 | 1.8% | 4 | -- | -- | -- | - | -- | -- | -- | - | -- | -- | -- | - |
| CPL | C15H24 | 204.19 | -- | -- | -- | - | -- | -- | -- | - | -- | -- | -- | - | -- | -- | -- | - |
| ART | C15H22O5 | 282.15 | 164.09 | 0.2 | 0.1% | 4 | 333.58 | 0.5 | 0.1% | 4 | 441.86 | 1.7 | 0.4% | 4 | -- | -- | -- | - |
| CIN | C19H22N2O | 294.17 | 174.20 | 1.9 | 1.1% | 4 | 344.94 | 1.9 | 0.6% | 4 | 441.28 | 1.8 | 0.4% | 4 | 380.59 | 7.9 | 2.1% | 3 |
| HEA | C29H44O5 | 472.32 | 216.15 | 0.9 | 0.4% | 4 | 364.78 | 4.7 | 1.3% | 4 | 452.79 | 2.5 | 0.5% | 2 | -- | -- | -- | - |
| IPG | C9H16O6 | 220.09 | 146.10 | 0.7 | 0.5% | 4 | 327.80 | 2.7 | 0.8% | 4 | 434.73 | 3.5 | 0.8% | 4 | 347.66 | 2.5 | 0.7% | 4 |
| CHL | C8H11Cl3O6 | 307.96 | 182.82 | 0.5 | 0.3% | 4 | 329.57 | 1.0 | 0.3% | 4 | 439.28 | 1.3 | 0.3% | 4 | 356.23 | 2.1 | 0.6% | 3 |
| CHR | C15H10O4 | 254.06 | 156.10 | 1.3 | 0.8% | 4 | 333.81 | 2.7 | 0.8% | 4 | 439.42 | 3.0 | 0.7% | 4 | 356.62 | 0.3 | 0.1% | 3 |
| FIS | C15H10O6 | 286.05 | -- | -- | -- | - | -- | -- | -- | - | -- | -- | -- | - | -- | -- | -- | - |
| QUE | C15H10O7 | 302.04 | 163.67 | 0.8 | 0.5% | 4 | -- | -- | -- | - | -- | -- | -- | - | -- | -- | -- | - |
| RUT | C27H30O16 | 610.15 | 217.74 | 1.5 | 0.7% | 3 | 362.11 | 2.8 | 0.8% | 4 | -- | -- | -- | - | -- | -- | -- | - |
| | | peak 2= | 232.25 | 2.5 | 1.1% | 4 | | | | | | | | | | | | |
| | | | | | | | | | | | | | | | | | | |
| | | | [βCD - H]- | | | | [2βCD - H]+ | | | | | | | | | | | |
| βCD | C42H70O35 | 1134.37 | 309.28 | 0.5 | 0.2% | 3 | 426.94 | 0.7 | 0.2% | 3 | | | | | | | | |
| | | | -- | -- | -- | - | -- | -- | -- | - | | | | | | | | |

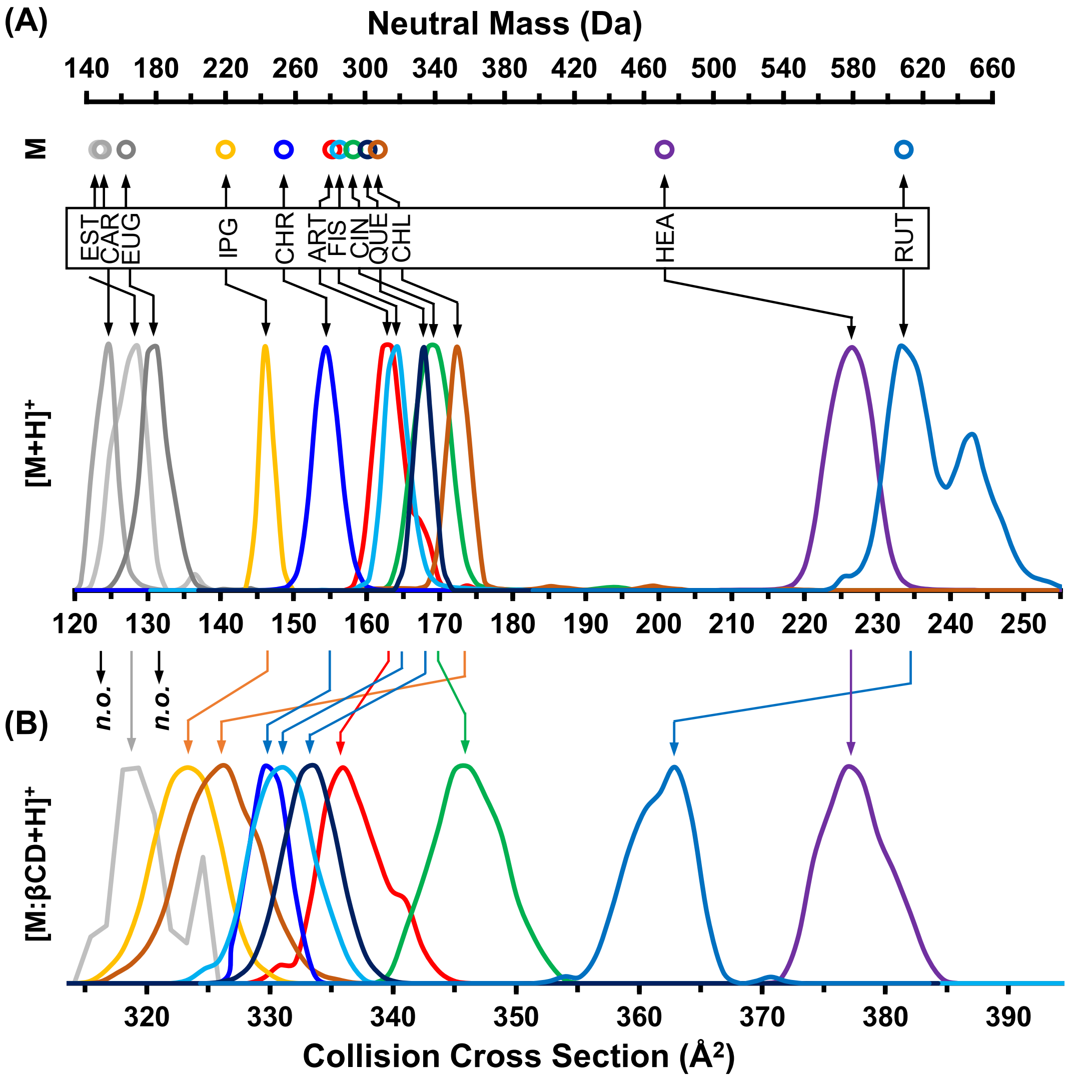


**Figure S7. (A)** Neutral mass ordering of the guest molecules and the ion mobility profiles and relative CCS ordering of their protonated ion forms. **(B)** IM profiles and the CCS ordering of the protonated M:βCD guest:host complexes.

## Appendix 5 – Higher Order Complexes with 1:2 and 2:1 Stoichiometries

Higher order complexes were observed in the IM-MS survey of samples. Of note is the 1:2 guest:host complex, whereby one guest molecule is bound to two βCD hosts. This 1:2 complex was observed prominently for the two acetal pentoses (IPG and CHL, >1E3 counts) in both ion modes, and trace abundances (<1E3) of this complex were also seen for several of the other guests, including ART, CIN, and FIS. The 1:2 complexes did not respond to changes in sample concentration (**Figure S5**), which suggests these might be specific guest:host binding, such as a "capped" sandwich or "threaded" rotaxane structure. The lower abundances precluded the acquisition of MS/MS data, however reproducible CCS measurements (RSD<1%) were obtained for several protonated and deprotonated 1:2 complexes (**Table S3**). An interesting observation here is that the change in CCS between a βCD dimer and the addition of the guest molecule (i.e., 0:2 to 1:2 complexation) results in almost no change in CCS (**Figure S8A**), and in fact for many of these protonated 1:2 complexes, the gas-phase structure collapses to a smaller CCS, which implies tight inclusion of the guest molecule as might be expected from complete guest inclusion within the βCD dimer.(Berland *et al.*, 2015) Previous computational results on βCD dimer complexed with various guests have suggested that this complex is stabilized through hydrogen bonding between the secondary hydroxyl groups of both complexes, forming a capped "sandwich" in which the guest is threaded within the hydrophobic cores of both CDs.(Christoforides *et al.*, 2018; Makedonopoulou *et al.*, 2000) A similar pseudorotaxane structure whereby the guest occupies both βCD cavities is likely being adopted in these examples.

Results for the deprotonated $[M:2\beta CD-H]^-$ complexes show a less dramatic change in structure, where the changes in CCS range from 3-8% (**Figure S8A**), which are more in line with the CCS differences observed for the 1:1 complex as compared to uncomplexed βCD host. This points to a strong influence of the charge carrier on the observed anhydrous structures. Negative ion mode results for 1:2 ion complexes have not been previously reported. It should also be noted here that the ion mobility profiles for βCD and βCD dimer exhibit multiple mobility features for the protonated ion forms, and single profiles for the deprotonated forms (**Figure S8B**), implying βCD and its dimer adopt multiple anhydrous structures that are charge-directed, which makes the interpretation of the observed changes in CCS challenging.

The 2:1 guest:host complex, whereby two guests are bound to one βCD, was observed in low abundance in five systems: two acetyl pentoses (IPG and CHL), two flavones (CHR and FIS), and one alkaloid (CIN) in negative ion mode. Notably, these four systems also exhibited intense ion signals for their corresponding 1:1 complexes, which implies strong binding of the first guest is a prerequisite for accommodating a second guest.

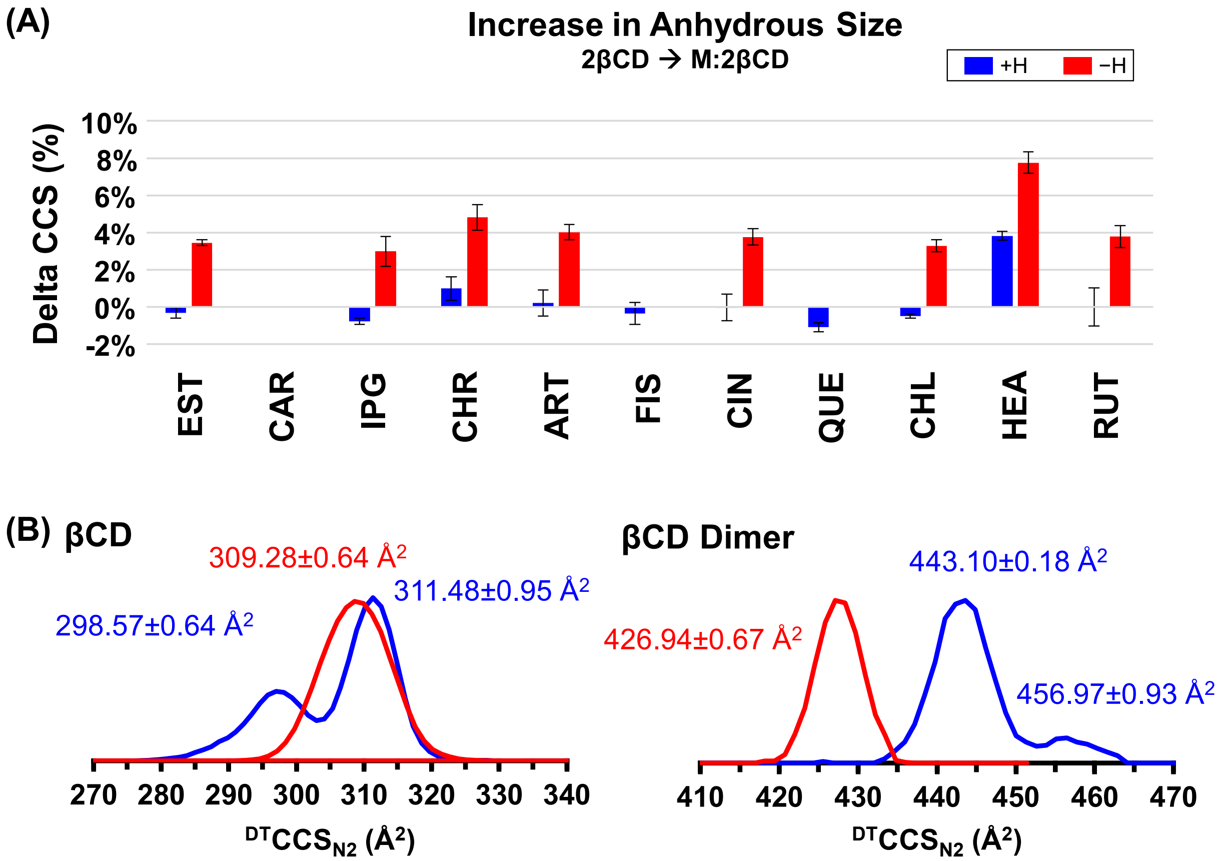


**Figure S8. (A)** The measured change in CCS between unbound βCD dimer (2βCD) and the two-host complex (M:2βCD) for both protonated (blue) and deprotonated (red) ions. **(B)** IM profiles for both the βCD monomer and βCD dimer with overlay for the protonated and deprotonated ion forms.

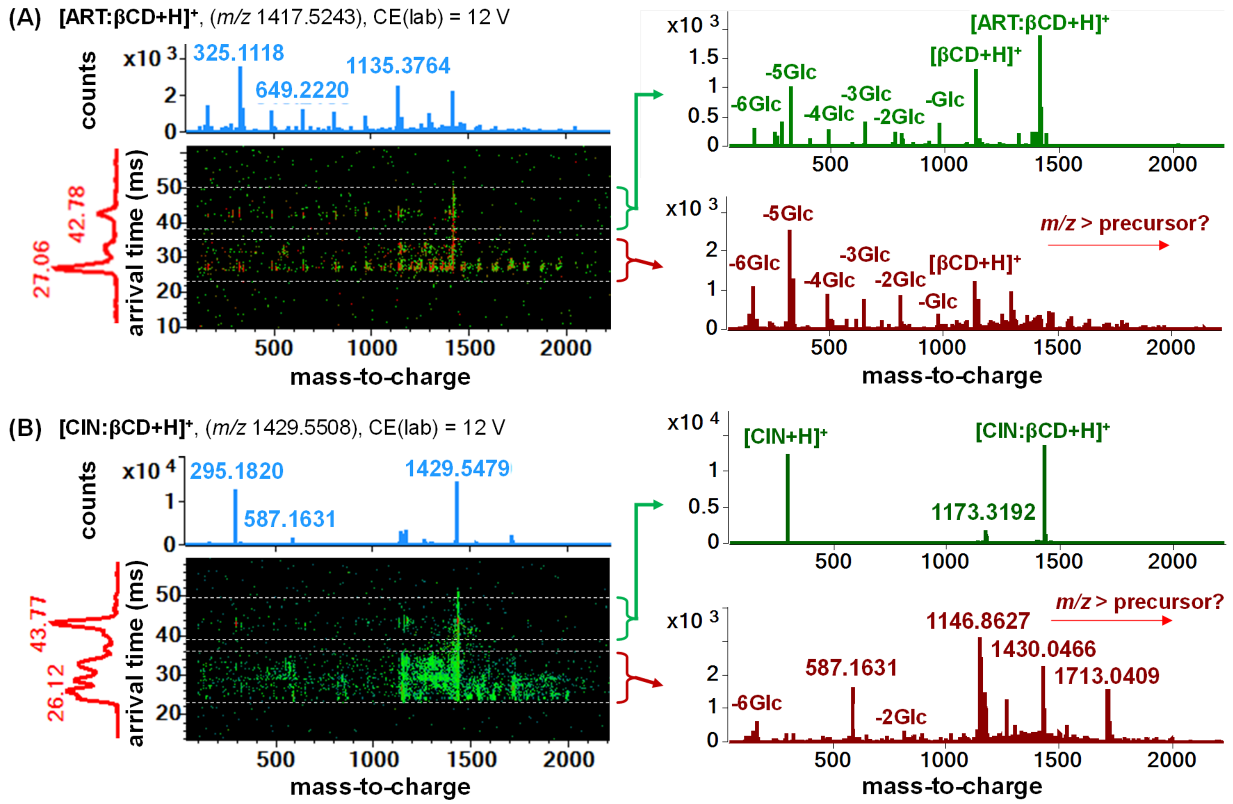


**Figure S9.** Example data showing the analytical benefit of performing IM separations prior to mass-selected ion fragmentation (i.e., IM-MS/MS). **(A)** IM/MS fragmentation spectrum (12V lab frame) of quadrupole-isolated artemisinin complex, [ART:βCD+H]+ (*m/z* 1417.5, 3 Da window), which exhibits characteristic losses of glucose (Glc) sub-units but also significant ion signal contributing to the baseline. Some signals appear at higher *m/z* than the precursor, indicating these are interferent ions originating from co-isolated multiply-charged multimers. Because IM separation precedes MS/MS, these isobaric interferent precursors and associated fragments can be filtered out in the IM dimension (dark red mass spectra, at right) to yield “clean” ion fragmentation data that is associated only with the target charge state of interest (dark green spectra). **(B)** Example of IM-filtered MS/MS data for the cinchonine complex, [CIN:βCD+H]+ (*m/z* 1429.6), showing similar isobaric interferences that are removed through the IM dimension. IM filtering significantly improves the quality and accuracy of the MS/MS results, particularly for analytes within a complex sample matrix such as βCD.

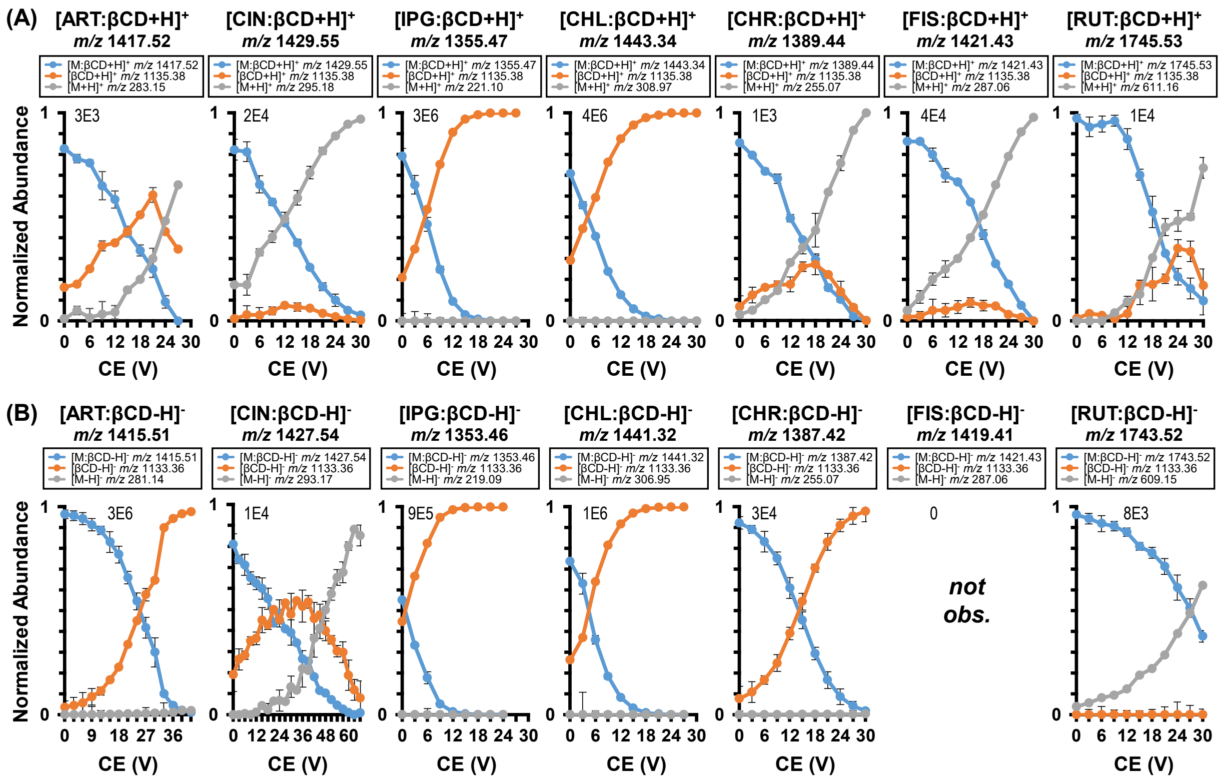


**Figure S10.** Energy-resolved ion abundance plots (normalized to 1) of **(A)** protonated, and **(B)** deprotonated 1:1 M:βCD complexes. The precursor complex (blue traces), βCD (orange), and small molecule (grey) ion abundances are shown across the collision energy (CE, laboratory frame) range surveyed. The measured abundance (in ion counts) of the precursor at 0V is shown in the upper left in each plot. Of note is that the βCD ion is always formed in high abundance as a charge-carrying product from dissociation of the noncovalent guest:host complex. The observed depletion of βCD in some of the abundance plots coincides with characteristic loss of glucose sub-units from βCD (*c.f.*, **Figure S11**).

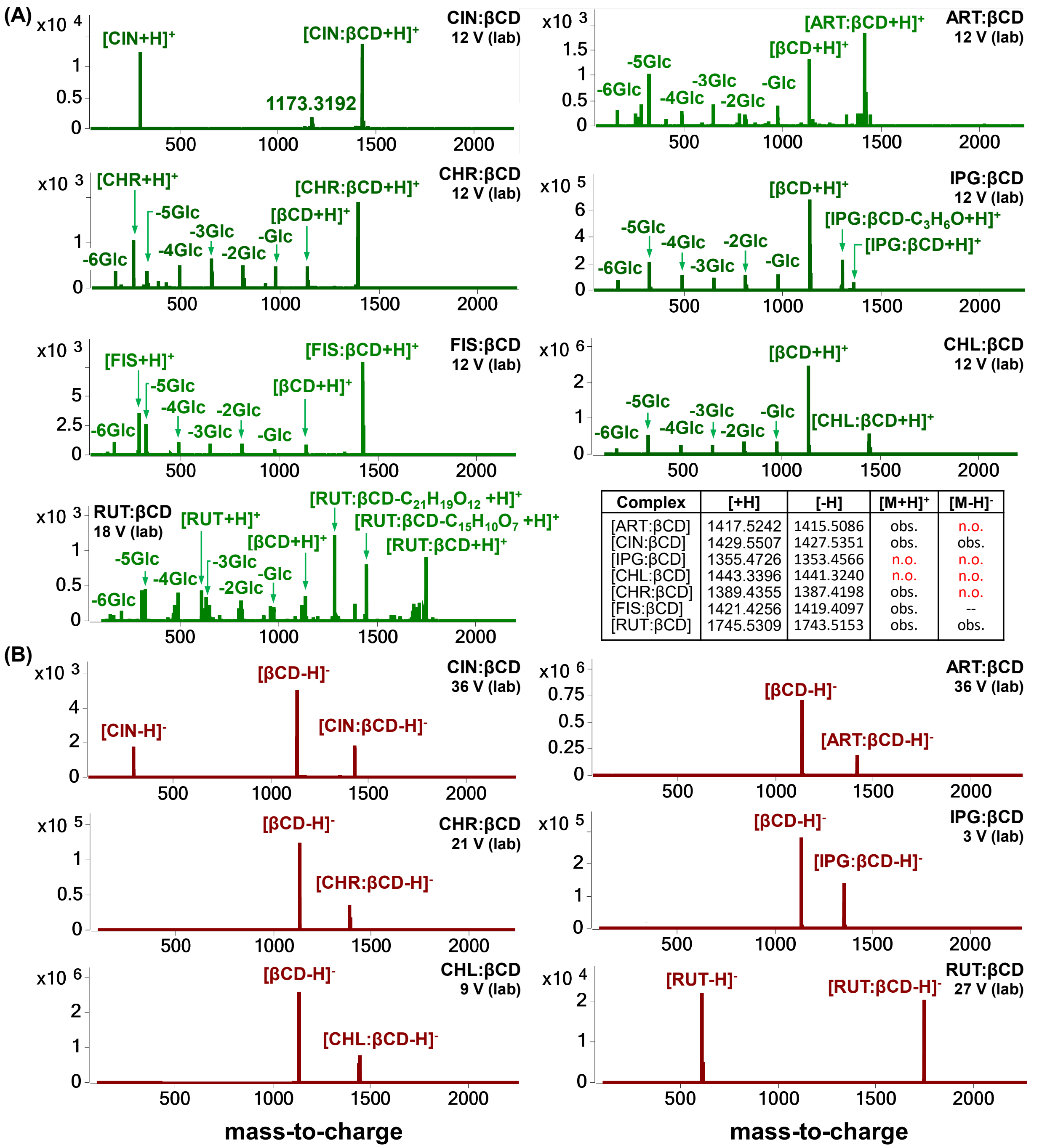


| Complex | [+H] | [-H] | $[M+H]^+$ | $[M-H]^-$ |
|---|---|---|---|---|
| [ART:βCD] | 1417.5242 | 1415.5086 | obs. | n.o. |
| [CIN:βCD] | 1429.5507 | 1427.5351 | obs. | obs. |
| [IPG:βCD] | 1355.4726 | 1353.4566 | n.o. | n.o. |
| [CHL:βCD] | 1443.3396 | 1441.3240 | n.o. | n.o. |
| [CHR:βCD] | 1389.4355 | 1387.4198 | obs. | n.o. |
| [FIS:βCD] | 1421.4256 | 1419.4097 | obs. | -- |
| [RUT:βCD] | 1745.5309 | 1743.5153 | obs. | obs. |

**Figure S11.** MS/MS fragmentation spectra (IM-filtered) for **(A)** protonated (dark green) and **(B)** deprotonated (dark red) small molecule:βCD 1:1 complexes. Observations for the appearance/ejection of the small molecule as a primary fragmentation channel are summarized in the embedded table for MS/MS of both protonated and deprotonated ion complexes. "obs." = observed; "n.o." = not observed. The y-axes project ion counts summed across the 1 minute MS/MS acquisitions.

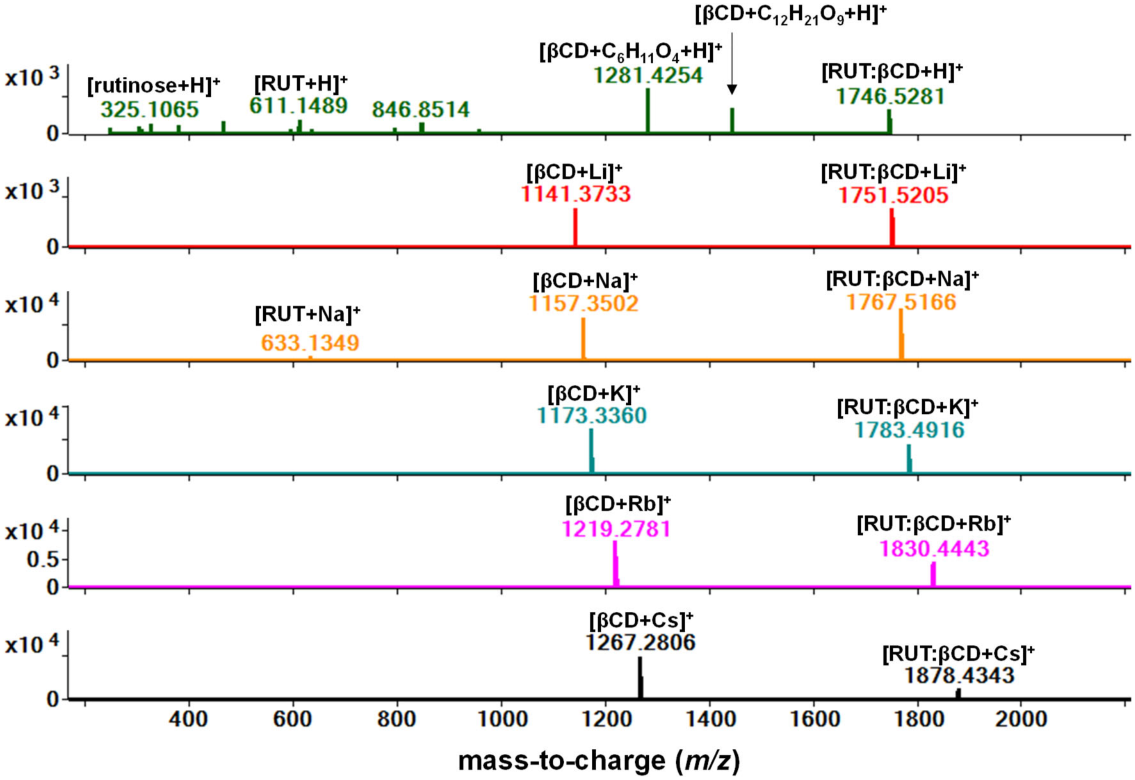


**Figure S12.** MS/MS fragmentation spectra (IM-filtered) for [RUT:βCD+X]+ where X=H, Li, Na, K, Rb, or Cs. Dissociation of the protonated complex (top spectrum) is the only case where partial fragmentation of the RUT:βCD complex is observed.

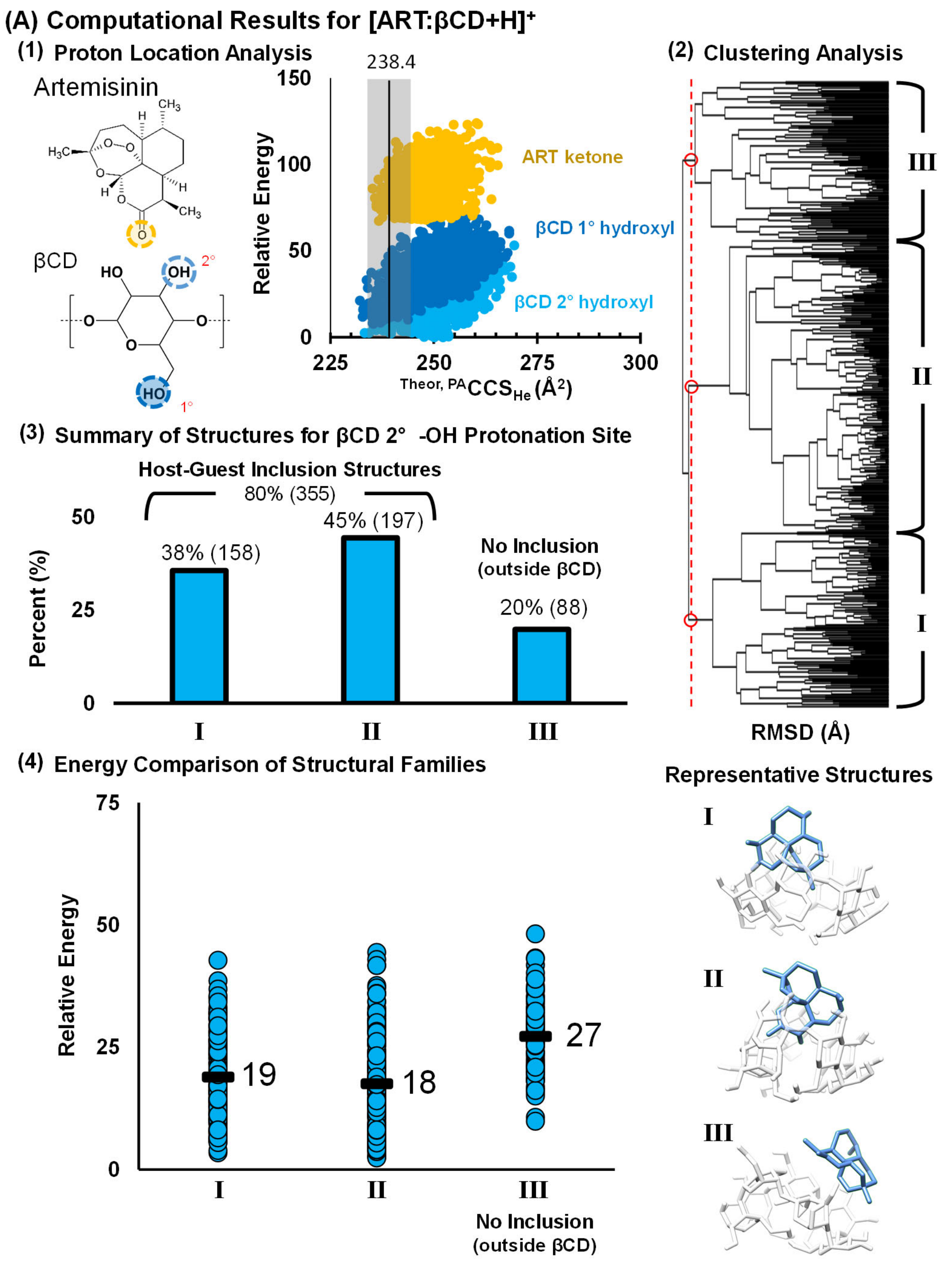


**Figure S13. (A)** Computational results summary for the protonated artemisinin-βCD complex, [ART:βCD+H]$^+$. **(1)** Theoretical CCS cluster plots for ART protonation (top, orange) and βCD protonation at the secondary (middle, light blue) and primary (bottom, dark blue) hydroxyl groups (larger and smaller βCD rims, respectively). **(2)** RMSD dendrogram of structures corresponding to ±3% of experimental CCS for protonation at the βCD 2° hydroxyl. Structural family groupings (Roman numerals) are defined using the RMSD threshold indicated by the dotted red line. **(3)** Evaluation of extent of inclusion for each structural family within the 2° hydroxyl protonation cluster (light blue). **(4)** Energy comparison of the structural families and representative structures closest to the average energy (horizontal bar) within each family.

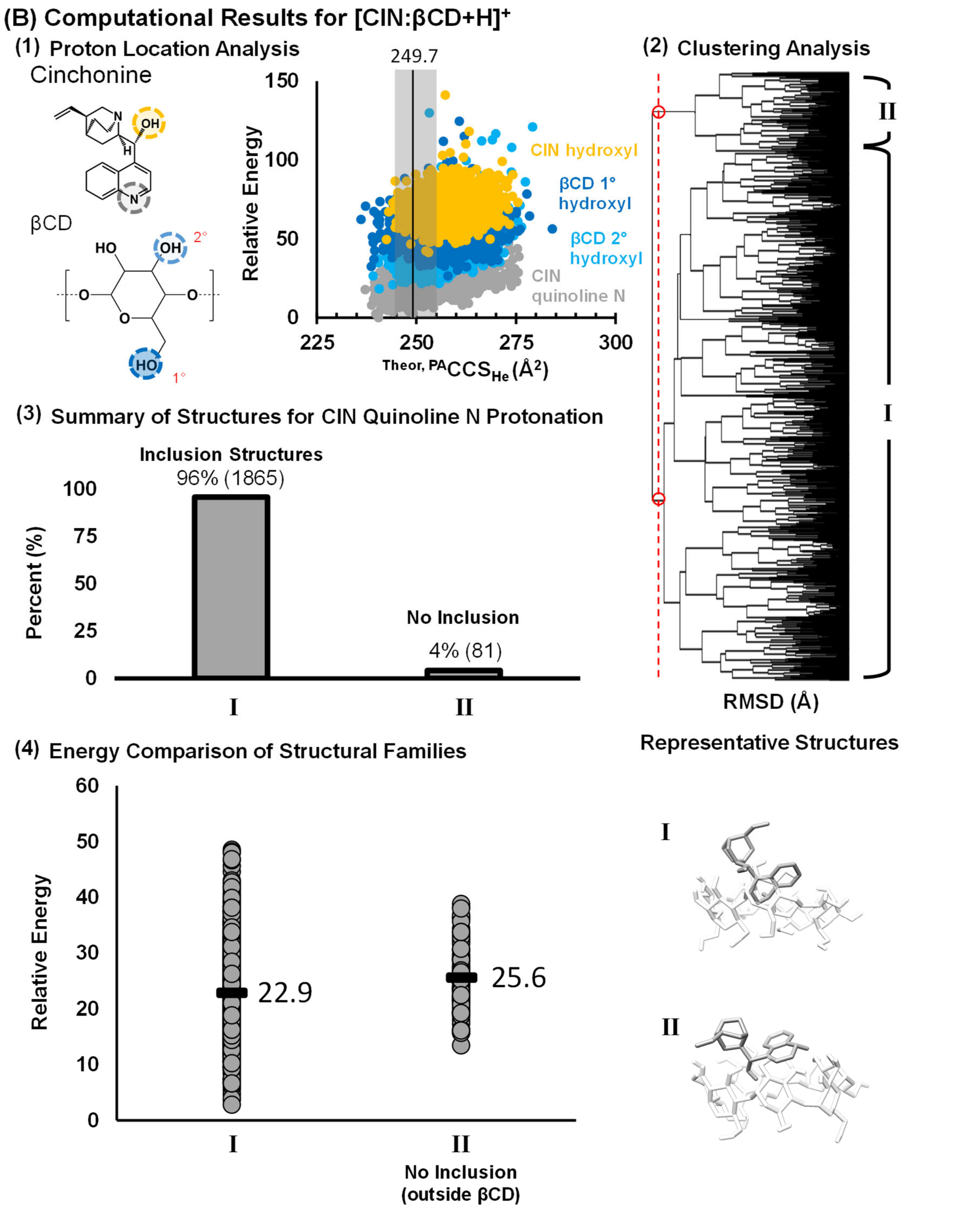


**Figure S13. (B)** Computational results for the protonated cinchonine-βCD complex, $[CIN{:}\beta CD{+}H]^{+}$. **(1)** Evaluation of probable protonation sites, **(2)** RMSD dendrogram for protonation at CIN (quinoline nitrogen, grey), **(3)** assessment of extent of inclusion for each structural family defined by the RMSD dendrogram threshold (dotted red line), and **(4)** energy comparison of the primary structural families with representative structures.

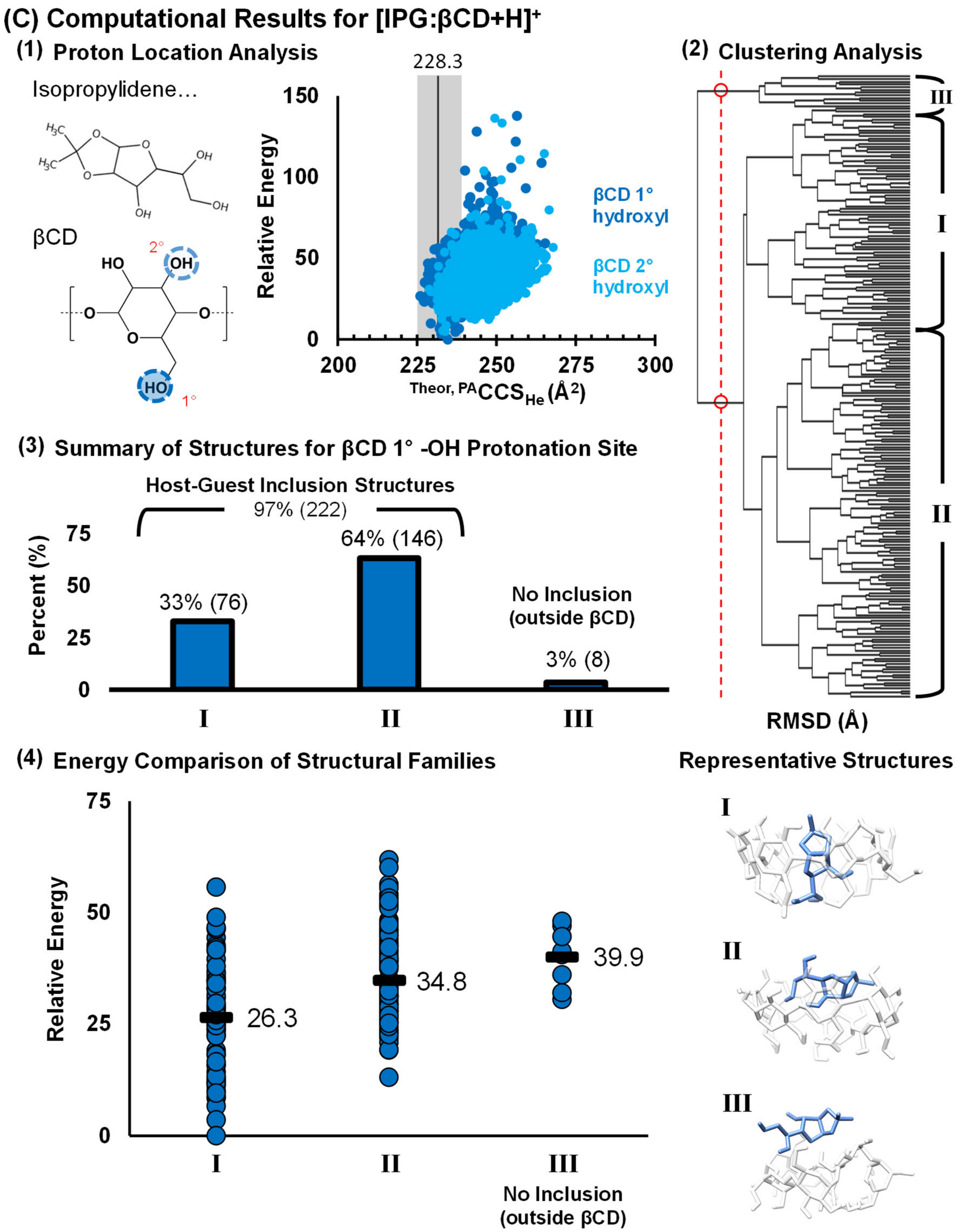


**Figure S13. (C)** Computational results for the protonated 1,2-O-Isopropyllidene-α-D-glucofuranose-βCD complex, [IPG:βCD+H]$^+$. **(1)** Evaluation of probable protonation sites, **(2)** RMSD dendrogram for structures with protonation at βCD primary hydroxyl group (dark blue), **(3)** assessment of extent of inclusion for each structural family defined from the dendrogram, and **(4)** energy comparison of the structural families with representative structures shown at right.

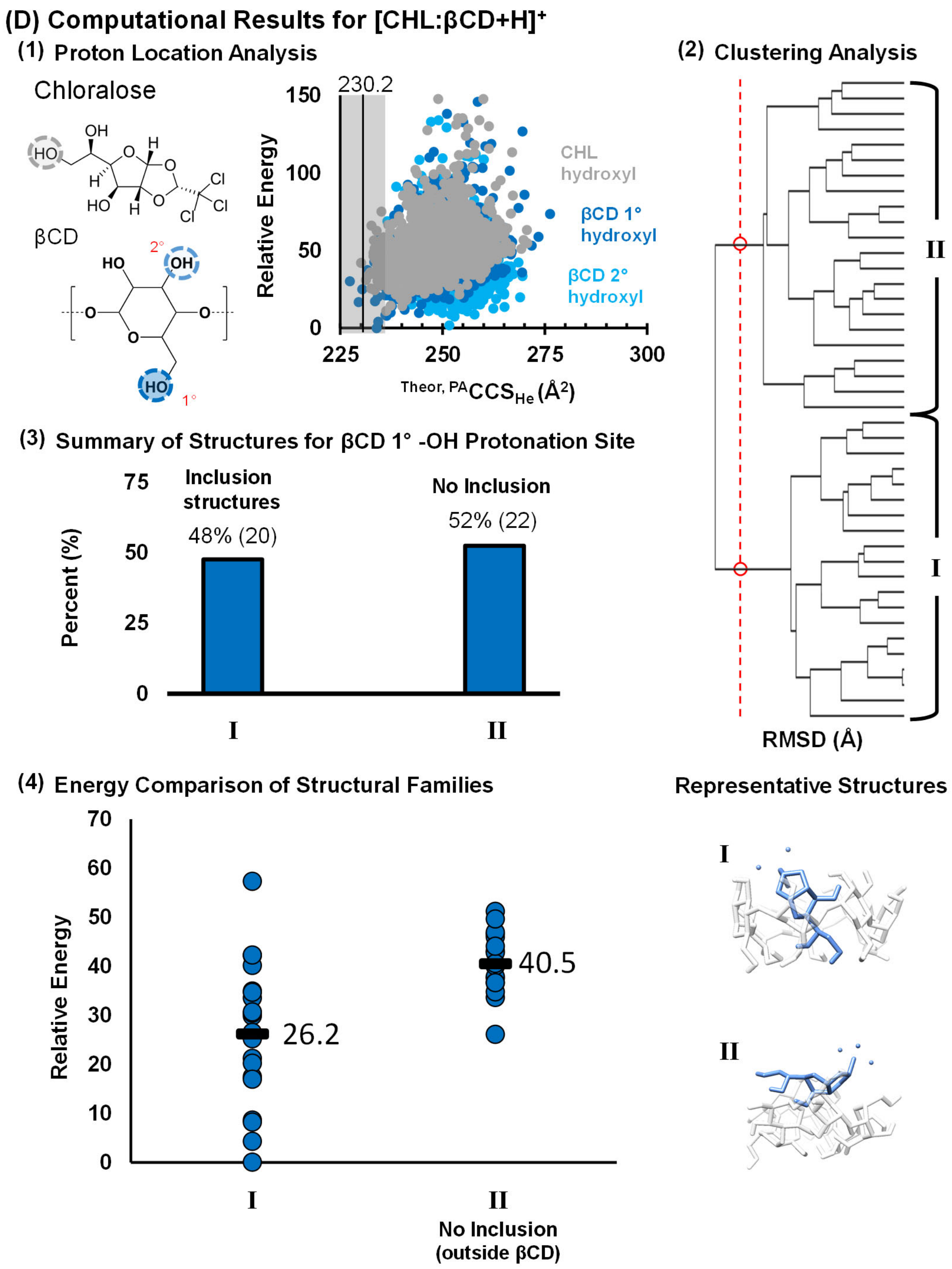


**Figure S13. (D)** Computational results for the protonated chloralose-βCD complex, [CHL:βCD+H]+. **(1)** Evaluation of probable protonation sites, **(2)** RMSD dendrogram for the cluster of theoretical structures with protonation at βCD primary hydroxyl group (dark blue), **(3)** assessment of extent of inclusion for the two structural families defined by the RMSD cutoff indicated in the dendrogram, and **(4)** energy comparison of the primary structural families with representative structures. For this example, fragmentation of CHL: CD did not indicate protonation at CHL, however CHL charge carrying structures were evaluated and resulted in a cluster of theoretical structures at higher energy than the two βCD protonation sites.

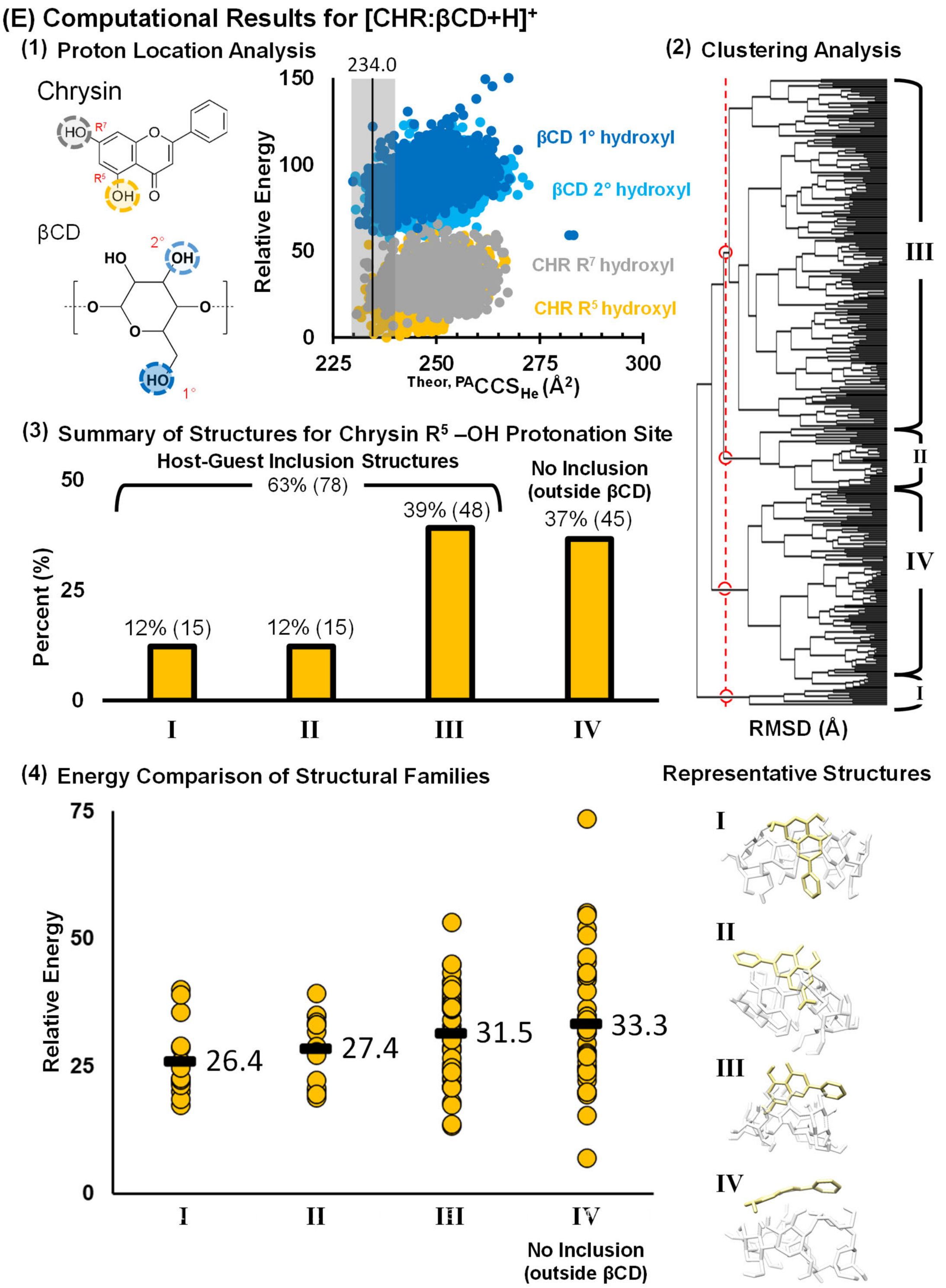


**Figure S13. (E)** Computational results for the protonated chrysin-βCD complex, $[CHR:\beta CD+H]^+$. **(1)** Evaluation of probable protonation sites, **(2)** RMSD dendrogram for theoretical structures with protonation at the CHR $R^5$ hydroxyl group (orange), **(3)** assessment of extent of inclusion for the four structural families indicated by the dendrogram, and **(4)** energy comparison of the primary structural families with representative structures.

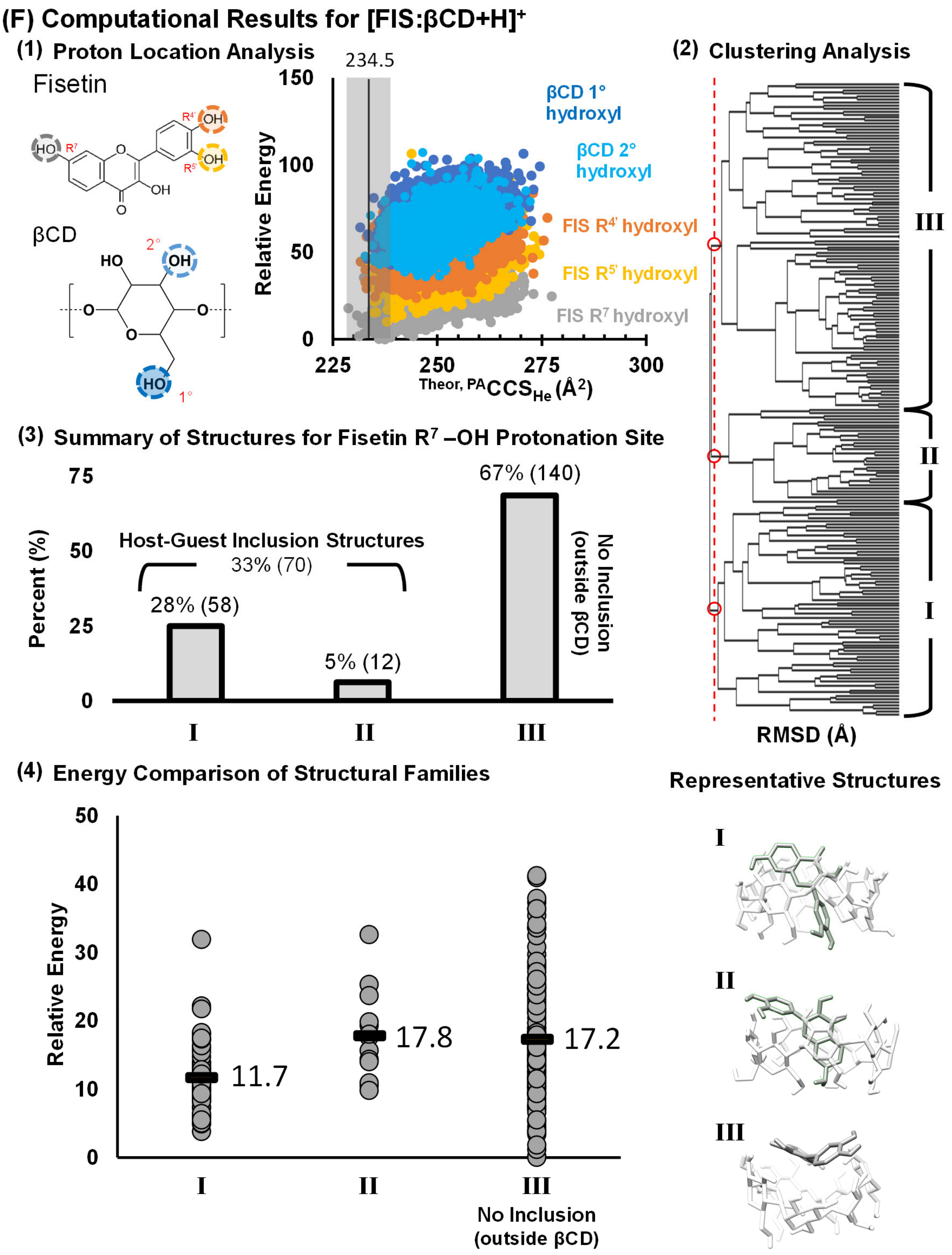


**Figure S13. (F)** Computational results for the protonated fisetin-βCD complex, [FIS:βCD+H]+. **(1)** Evaluation of probable protonation sites, **(2)** RMSD dendrogram for theoretical structures with protonation at the FIS R7 hydroxyl group (grey), **(3)** assessment of extent of inclusion for the families defined by RMSD analysis, and **(4)** energy comparison of the primary structural families with representative structures.

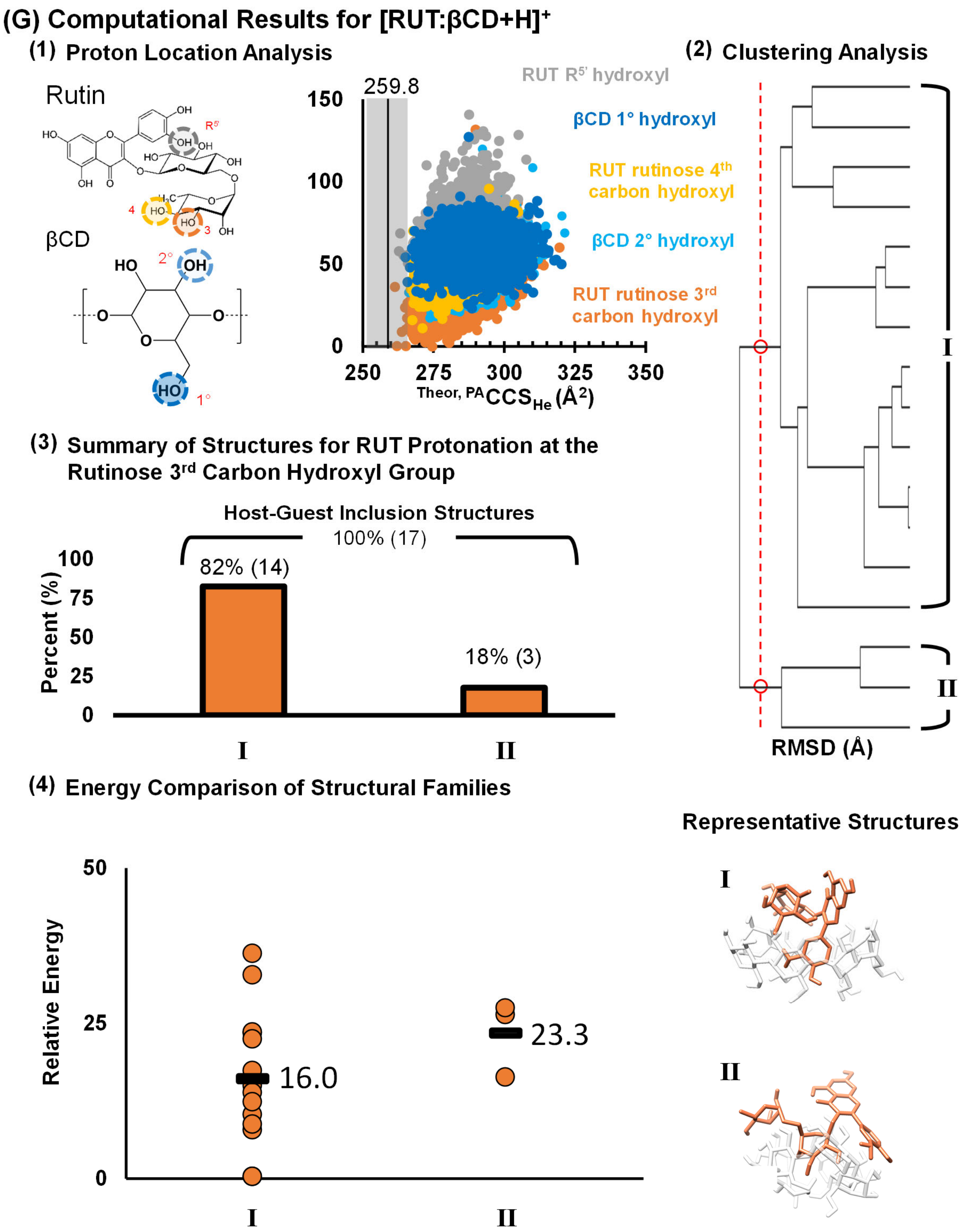


**Figure S13. (G)** Computational results for the protonated rutin-βCD complex, [RUT:βCD+H]+. **(1)** Evaluation of probable protonation sites, **(2)** RMSD dendrogram for theoretical structures with protonation at the RUT rutinose hydroxyl group at the 3rd carbon (orange), **(3)** assessment of extent of inclusion, and **(4)** energy comparison of the primary structural families with representative structures.

**Table S4.** Collision cross section measurements in nitrogen and helium for protonated βCD host and select M:βCD guest:host ion complexes. The percent difference in CCS (ΔCCS) observed across the two drift gases is summarized in the far-right column. ΔCCS is calculated as follows:

$$\Delta CCS = 2 \cdot \frac{(CCS_{N2} - CCS_{He})}{(CCS_{N2} + CCS_{He})}$$

| Ion Form | Exact *m/z* | $N_2$ CCS (Å$^2$) | He CCS (Å$^2$) | ΔCCS He->N2 (%) |
|---|---|---|---|---|
| $[\beta CD + H]^+$ | 1135.3776 | 311.5 | 220.8 | 34.1% |
| $[IPG{:}\beta CD + H]^+$ | 1355.4723 | 323.1 | 228.3 | 34.4% |
| $[CHR{:}\beta CD + H]^+$ | 1389.4355 | 330.1 | 234.0 | 34.1% |
| $[ART{:}\beta CD + H]^+$ | 1417.5243 | 336.1 | 238.4 | 34.0% |
| $[FIS{:}\beta CD + H]^+$ | 1421.4253 | 330.6 | 234.5 | 34.0% |
| $[CIN{:}\beta CD + H]^+$ | 1429.5508 | 346.5 | 249.7 | 32.5% |
| $[CHL{:}\beta CD + H]^+$ | 1443.3397 | 325.9 | 230.2 | 34.4% |
| $[RUT{:}\beta CD + H]^+$ | 1745.5309 | 361.3 | 259.8 | 32.7% |
| | | | Average= | 33.8% |
| | | | SD= | 0.8% |

**Table S5.** GAFF parameter files for cyclodextrin, artemisinin, fisetin, chloralose, cinchonine, rutin, isopropylidene, and chrysin.

```
              beta-CYCLODEXTRIN
0    0    2
bcd   INT  0
CORRECT     OMIT DU   BEG
  0.0000
   1  DUMM  DU    M    0  -1  -2     0.000      .0        .0      .00000
   2  DUMM  DU    M    1   0  -1     1.449      .0        .0      .00000
   3  DUMM  DU    M    2   1   0     1.523   111.21       .0      .00000
   4  O5    oh    M    3   2   1     1.540   111.208  -180.000 -0.659531
   5  H10   ho    E    4   3   2     0.947   100.898   -76.502  0.417040
   6  C6    c3    M    4   3   2     1.402    35.876   174.623  0.234719
   7  H8    h1    E    6   4   3     1.084   110.784    11.185  0.027474
   8  H9    h1    E    6   4   3     1.086   111.048   132.077  0.027474
   9  C5    c3    M    6   4   3     1.517   109.262  -108.945  0.099282
  10  H7    h1    E    9   6   4     1.085   108.053   170.506  0.059179
  11  O4    os    E    9   6   4     1.412   107.371   -71.254 -0.408436
  12  C4    c3    M    9   6   4     1.532   113.103    50.437  0.038883
  13  H6    h1    E   12   9   6     1.081   109.509   -52.765  0.128842
  14  O31   os    E   12   9   6     1.406   110.515    69.190 -0.359624
  15  C3    c3    M   12   9   6     1.519   110.209  -172.386  0.192567
  16  H4    h1    E   15  12   9     1.085   109.055   -67.217  0.065256
  17  O3    oh    S   15  12   9     1.404   107.972   173.340 -0.663957
  18  H5    ho    E   17  15  12     0.950   108.773  -177.106  0.439463
  19  C2    c3    M   15  12   9     1.518   110.076    52.287  0.247257
  20  H2    h1    E   19  15  12     1.086   108.712    63.107  0.056626
  21  O2    oh    S   19  15  12     1.407   106.894  -178.083 -0.659216
  22  H3    ho    E   21  19  15     0.948   110.796  -160.256  0.409334
  23  C1    c3    M   19  15  12     1.529   110.212   -55.056  0.196273
  24  H1    h2    E   23  19  15     1.081   110.994   172.914  0.111096
  25  O1    os    M   23  19  15     1.386   109.297   -64.848 -0.359624
  26  C10   c3    M   25  23  19     1.416   120.957  -128.576  0.038883
  27  H16   h1    E   26  25  23     1.082   109.952    11.882  0.128842
  28  C11   c3    3   26  25  23     1.527   109.530  -108.276  0.099282
  29  H17   h1    E   28  26  25     1.084   108.405   -49.308  0.059179
  30  O9    os    E   28  26  25     1.421   110.171  -169.490 -0.408436
  31  C12   c3    3   28  26  25     1.520   113.562    72.771  0.234719
  32  H18   h1    E   31  28  26     1.078   110.130   -59.469  0.027474
  33  H19   h1    E   31  28  26     1.087   108.664  -178.081  0.027474
  34  O10   oh    S   31  28  26     1.398   111.885    59.195 -0.659531
  35  H20   ho    E   34  31  28     0.949   108.478    60.597  0.417040
  36  C9    c3    M   26  25  23     1.526   107.126   129.498  0.192567
  37  H14   h1    E   36  26  25     1.087   108.937    53.012  0.065256
  38  O8    oh    S   36  26  25     1.394   109.829   -68.397 -0.663957
  39  H15   ho    E   38  36  26     0.949   108.809    64.548  0.439463
  40  C8    c3    M   36  26  25     1.524   109.750   172.209  0.247257
  41  H12   h1    E   40  36  26     1.085   107.654    63.352  0.056626
  42  O7    oh    S   40  36  26     1.387   113.108  -178.440 -0.659216
  43  H13   ho    E   42  40  36     0.951   111.069    97.905  0.409334
  44  C7    c3    M   40  36  26     1.526   109.950   -53.272  0.196273
  45  H11   h2    E   44  40  36     1.080   109.691   172.099  0.111096
  46  O6    os    M   44  40  36     1.388   109.251   -66.776 -0.359624
  47  C16   c3    M   46  44  40     1.412   119.191  -139.261  0.038883
  48  H26   h1    E   47  46  44     1.083   109.984    -3.349  0.128842
  49  C17   c3    3   47  46  44     1.528   110.820  -123.336  0.099282
  50  H27   h1    E   49  47  46     1.085   109.129   -58.059  0.059179
  51  O14   os    E   49  47  46     1.416   108.635  -176.937 -0.408436
  52  C18   c3    3   49  47  46     1.525   114.162    62.829  0.234719
  53  H28   h1    E   52  49  47     1.085   108.687   -83.260  0.027474
  54  H29   h1    E   52  49  47     1.082   107.695   160.417  0.027474
  55  O15   oh    S   52  49  47     1.397   114.496    41.151 -0.659531
  56  H30   ho    E   55  52  49     0.949   109.027   -85.548  0.417040
  57  C15   c3    M   47  46  44     1.517   109.105   116.194  0.192567
  58  H24   h1    E   57  47  46     1.086   108.922    57.877  0.065256
  59  O13   oh    S   57  47  46     1.402   108.192   -61.372 -0.663957
  60  H25   ho    E   59  57  47     0.951   108.249  -166.769  0.439463
  61  C14   c3    M   57  47  46     1.524   109.639   177.552  0.247257
  62  H22   h1    E   61  57  47     1.084   108.163    62.563  0.056626
  63  O12   oh    S   61  57  47     1.393   110.992   179.015 -0.659216
  64  H23   ho    E   63  61  57     0.955   111.248    82.613  0.409334
  65  C13   c3    M   61  57  47     1.525   109.882   -54.864  0.196273
  66  H21   h2    E   65  61  57     1.079   110.916   171.371  0.111096
  67  O11   os    M   65  61  57     1.394   108.602   -67.037 -0.359624
  68  C22   c3    M   67  65  61     1.409   120.670  -123.295  0.038883
  69  H36   h1    E   68  67  65     1.082   110.340    15.883  0.128842
  70  C23   c3    3   68  67  65     1.532   110.393  -105.193  0.099282
  71  H37   h1    E   70  68  67     1.086   108.474   -51.322  0.059179
  72  O19   os    E   70  68  67     1.413   110.324  -171.187 -0.408436
  73  C24   c3    3   70  68  67     1.516   113.150    68.490  0.234719
  74  H38   h1    E   73  70  68     1.084   108.780   -70.410  0.027474
  75  H39   h1    E   73  70  68     1.087   108.122   171.702  0.027474
  76  O20   oh    S   73  70  68     1.401   109.386    50.657 -0.659531
  77  H40   ho    E   76  73  70     0.947   109.615  -177.480  0.417040
  78  C21   c3    M   68  67  65     1.517   107.742   134.667  0.192567
  79  H34   h1    E   78  68  67     1.086   108.903    54.078  0.065256
  80  O18   oh    S   78  68  67     1.404   108.328   -65.068 -0.663957
  81  H35   ho    E   80  78  68     0.951   108.261  -166.051  0.439463
  82  C20   c3    M   78  68  67     1.522   110.143   173.917  0.247257
  83  H32   h1    E   82  78  68     1.084   108.430    61.965  0.056626
  84  O17   oh    S   82  78  68     1.393   110.956   178.636 -0.659216
  85  H33   ho    E   84  82  78     0.954   111.200    81.708  0.409334
  86  C19   c3    M   82  78  68     1.524   109.667   -55.389  0.196273
  87  H31   h2    E   86  82  78     1.080   110.949   173.194  0.111096
  88  O16   os    M   86  82  78     1.395   108.569   -65.138 -0.359624
  89  C28   c3    M   88  86  82     1.408   121.230  -123.380  0.038883
  90  H46   h1    E   89  88  86     1.083   110.340    14.543  0.128842
  91  C29   c3    3   89  88  86     1.532   110.063  -106.553  0.099282
  92  H47   h1    E   91  89  88     1.084   108.633   -48.977  0.059179
  93  O24   os    E   91  89  88     1.418   110.319  -169.366 -0.408436
  94  C30   c3    3   91  89  88     1.519   113.191    72.875  0.234719
  95  H48   h1    E   94  91  89     1.079   110.203   -57.971  0.027474
  96  H49   h1    E   94  91  89     1.089   108.456  -176.432  0.027474
  97  O25   oh    S   94  91  89     1.398   111.818    61.033 -0.659531
  98  H50   ho    E   97  94  91     0.949   108.194    58.398  0.417040
  99  C27   c3    M   89  88  86     1.518   107.395   133.200  0.192567
 100  H44   h1    E   99  89  88     1.086   108.571    52.595  0.065256
 101  O23   oh    S   99  89  88     1.404   108.301   -66.437 -0.663957
 102  H45   ho    E  101  99  89     0.950   108.354  -166.616  0.439463
 103  C26   c3    M   99  89  88     1.522   110.684   172.269  0.247257
 104  H42   h1    E  103  99  89     1.084   108.594    62.717  0.056626
 105  O22   oh    S  103  99  89     1.394   110.986   179.238 -0.659216
 106  H43   ho    E  105 103  99     0.955   110.979    75.073  0.409334
 107  C25   c3    M  103  99  89     1.525   109.729   -54.934  0.196273
 108  H41   h2    E  107 103  99     1.079   111.106   173.560  0.111096
 109  O21   os    M  107 103  99     1.393   109.245   -64.145 -0.359624
 110  C34   c3    M  109 107 103     1.408   120.603  -115.841  0.038883
 111  H56   h1    E  110 109 107     1.081   110.607    16.587  0.128842
 112  C35   c3    3  110 109 107     1.532   110.143  -104.875  0.099282
 113  H57   h1    E  112 110 109     1.085   108.174   -51.244  0.059179
 114  O29   os    E  112 110 109     1.411   110.310  -171.232 -0.408436
 115  C36   c3    3  112 110 109     1.515   113.341    68.309  0.234719
 116  H58   h1    E  115 112 110     1.086   108.964   -70.564  0.027474
 117  H59   h1    E  115 112 110     1.086   107.903   171.564  0.027474
 118  O30   oh    S  115 112 110     1.402   109.343    50.570 -0.659531
 119  H60   ho    E  118 115 112     0.946   109.749   170.646  0.417040
 120  C33   c3    M  110 109 107     1.517   107.561   135.855  0.192567
 121  H54   h1    E  120 110 109     1.085   109.080    53.904  0.065256
 122  O28   oh    S  120 110 109     1.405   108.482   -65.536 -0.663957
 123  H55   ho    E  122 120 110     0.950   108.472  -172.084  0.439463
 124  C32   c3    M  120 110 109     1.518   110.444   173.276  0.247257
 125  H52   h1    E  124 120 110     1.088   108.650    61.832  0.056626
 126  O27   oh    S  124 120 110     1.397   106.981  -179.267 -0.659216
 127  H53   ho    E  126 124 120     0.952   111.125   150.499  0.409334
 128  C31   c3    M  124 120 110     1.528   110.075   -54.903  0.196273
 129  H51   h2    E  128 124 120     1.079   110.384   171.136  0.111096
 130  O26   os    M  128 124 120     1.392   109.670   -67.223 -0.359624
 131  C40   c3    M  130 128 124     1.407   119.069  -128.979  0.038883
 132  H66   h1    E  131 130 128     1.083   110.486   -27.979  0.128842
 133  C39   c3    M  131 130 128     1.521   110.226    93.345  0.192567
 134  H64   h1    E  133 131 130     1.086   108.386    49.530  0.065256
 135  O33   oh    S  133 131 130     1.410   109.035   -69.473 -0.663957
 136  H65   ho    E  135 133 131     0.952   107.778  -163.729  0.439463
 137  C38   c3    M  133 131 130     1.522   110.826   169.504  0.247257
 138  H62   h1    E  137 133 131     1.084   108.602    63.256  0.056626
 139  O32   oh    S  137 133 131     1.395   110.821   179.670 -0.659216
 140  H63   ho    E  139 137 133     0.955   110.724    76.569  0.409334
 141  C37   c3    M  137 133 131     1.523   110.027   -54.603  0.196273
 142  H61   h2    E  141 137 133     1.079   111.041   173.217  0.111096
 143  O34   os    M  141 137 133     1.389   109.270    56.227 -0.408436
 144  C41   c3    M  143 141 137     1.418   117.184   -59.571  0.099282
 145  H67   h1    E  144 143 141     1.085   109.761   -62.795  0.059179
 146  C42   c3    M  144 143 141     1.520   105.295   179.525  0.234719
 147  H68   h1    E  146 144 143     1.079   109.823  -176.346  0.027474
 148  H69   h1    E  146 144 143     1.087   108.605    65.327  0.027474
 149  O35   oh    M  146 144 143     1.397   111.971   -57.520 -0.659531
 150  H70   ho    E  149 146 144     0.949   108.178    57.966  0.417040

LOOP
   C1   O4
  C37  O31
   C7   O9
  C13  O14
  C19  O19
  C25  O24
  C31  O29
  C41  C40

IMPROPER

DONE
STOP
```

```
              ARTEMISININ
0    0    2
art   INT  0
CORRECT     OMIT DU   BEG
  0.0000
   1  DUMM  DU    M    0  -1  -2     0.000      .0        .0      .00000
   2  DUMM  DU    M    1   0  -1     1.449      .0        .0      .00000
   3  DUMM  DU    M    2   1   0     1.523   111.21       .0      .00000
   4  C1    c3    M    3   2   1     1.540   111.208  -180.000 -0.316894
   5  H6    hc    E    4   3   2     1.083   109.810   -48.581  0.075688
   6  H7    hc    E    4   3   2     1.086   127.023   178.943  0.075688
   7  H8    hc    E    4   3   2     1.084    25.650  -137.159  0.075688
   8  C2    c3    M    4   3   2     1.538    87.429    65.920  0.249262
   9  H1    hc    E    8   4   3     1.087   105.325    60.720 -0.016911
  10  C3    c3    M    8   4   3     1.548   113.511   176.475 -0.365567
  11  H9    hc    E   10   8   4     1.085   108.351  -146.613  0.091727
  12  H10   hc    E   10   8   4     1.085   108.718   -32.062  0.091727
  13  C4    c3    M   10   8   4     1.550   115.677    91.479  0.183897
  14  H11   hc    E   13  10   8     1.082   109.307  -137.364 -0.025161
  15  H12   hc    E   13  10   8     1.088   109.906   107.256 -0.025161
  16  C5    c3    M   13  10   8     1.532   112.546   -15.611 -0.113026
  17  H2    hc    E   16  13  10     1.082   107.604   -58.273  0.106632
  18  C6    c3    M   16  13  10     1.543   116.247  -176.218 -0.006297
  19  H3    hc    E   18  16  13     1.087   105.283   167.603  0.098443
  20  C15   c3    3   18  16  13     1.528   114.273    49.347 -0.455685
  21  H20   hc    E   20  18  16     1.083   111.987   -71.292  0.126669
  22  H21   hc    E   20  18  16     1.086   110.168    49.551  0.126669
  23  H22   hc    E   20  18  16     1.081   110.268   169.135  0.126669
  24  C7    c     M   18  16  13     1.517   114.417   -80.391  0.771766
  25  O1    o     E   24  18  16     1.183   123.636   149.998 -0.562236
  26  O2    os    M   24  18  16     1.340   116.662   -36.150 -0.515263
  27  C8    c3    M   26  24  18     1.405   122.049    34.959  0.604528
  28  H4    h2    E   27  26  24     1.079   107.185    78.845  0.034410
  29  O3    os    E   27  26  24     1.371   108.416  -166.545 -0.567801
  30  C9    c3    M   27  26  24     1.526   114.180   -42.577  0.050005
  31  C10   c3    M   30  27  26     1.560   107.491   175.495  0.022390
  32  H5    hc    E   31  30  27     1.088   106.933   -19.261 -0.001977
  33  C11   c3    M   31  30  27     1.534   109.873    96.934 -0.132010
  34  H13   hc    E   33  31  30     1.081   109.273    66.125  0.069323
  35  H14   hc    E   33  31  30     1.087   108.814  -177.505  0.069323
  36  C12   c3    M   33  31  30     1.538   114.658   -56.223 -0.202891
  37  H15   hc    E   36  33  31     1.085   109.926   -81.178  0.057914
  38  H16   hc    E   36  33  31     1.085   108.725   161.878  0.057914
  39  C13   c3    M   36  33  31     1.531   116.587    38.993  0.762372
  40  C14   c3    3   39  36  33     1.517   112.680   169.323 -0.518730
  41  H17   hc    E   40  39  36     1.082   110.525   -60.739  0.146953
  42  H18   hc    E   40  39  36     1.083   109.375    59.660  0.146953
  43  H19   hc    E   40  39  36     1.081   109.192   178.698  0.146953
  44  O4    os    M   39  36  33     1.396   113.848    49.966 -0.289110
  45  O5    os    M   44  39  36     1.399   112.705   -55.885 -0.254838

LOOP
  C10   C2
   C9   C5
  C13   O3
   O5   C9

IMPROPER
   C6   O1   C7   O2

DONE
STOP
```

```
              FISETIN
0    0    2
fis   INT  0
CORRECT     OMIT DU   BEG
  0.0000
   1  DUMM  DU    M    0  -1  -2     0.000      .0        .0      .00000
   2  DUMM  DU    M    1   0  -1     1.449      .0        .0      .00000
   3  DUMM  DU    M    2   1   0     1.523   111.21       .0      .00000
   4  O6    oh    M    3   2   1     1.540   111.208  -180.000 -0.630525
   5  H10   ho    E    4   3   2     0.949    78.760   -30.394  0.480915
```

```
    6  C15   ca    M    4   3   2     1.344  167.439 -164.728  0.367165
    7  C14   ca    B    6   4   3     1.377  120.019  -48.915 -0.324408
    8  C10   ca    S    7   6   4     1.389  120.341 -179.929 -0.227951
    9  H4    ha    E    8   7   6     1.074  119.264  178.107  0.186651
   10  H6    ha    E    7   6   4     1.074  118.704   -0.801  0.223961
   11  C13   ca    M    6   4   3     1.396  120.687  131.369  0.309111
   12  O5    oh    S   11   6   4     1.360  115.303   -0.169 -0.687263
   13  H9    ho    E   12  11   6     0.947  111.432  177.061  0.492062
   14  C9    ca    M   11   6   4     1.372  120.564 -179.941 -0.438966
   15  H3    ha    E   14  11   6     1.076  119.805 -179.669  0.208556
   16  C4    ca    M   14  11   6     1.398  120.397    0.098  0.120347
   17  C1    cc    M   16  14  11     1.481  119.533 -179.869  0.157655
   18  C5    cd    B   17  16  14     1.335  126.010 -137.391 -0.044204
   19  O2    oh    S   18  17  16     1.351  124.381    0.429 -0.556911
   20  H7    ho    E   19  18  17     0.948  110.701   22.972  0.421743
   21  C6    c     S   18  17  16     1.478  121.092 -178.969  0.799667
   22  O3    o     E   21  18  17     1.197  122.684  178.501 -0.568729
   23  O1    os    M   17  16  14     1.360  111.662   40.115 -0.326069
   24  C3    ca    M   23  17  16     1.343  121.130  179.317  0.566139
   25  C8    ca    S   24  23  17     1.388  116.779 -179.136 -0.595627
   26  H2    ha    E   25  24  23     1.073  120.726    0.143  0.247247
   27  C2    ca    M   24  23  17     1.386  121.330    0.631 -0.584539
   28  C7    ca    M   27  24  23     1.398  118.245 -179.809  0.190703
   29  H1    ha    E   28  27  24     1.074  118.025 -179.833  0.130033
   30  C12   ca    M   28  27  24     1.370  121.104    0.026 -0.593772
   31  H5    ha    E   30  28  27     1.075  120.734  179.980  0.212169
   32  C11   ca    M   30  28  27     1.402  119.326   -0.010  0.656812
   33  O4    oh    M   32  30  28     1.343  121.679  179.999 -0.644508
   34  H8    ho    E   33  32  30     0.947  111.002   -0.585  0.452538

LOOP
   C4  C10
   C2   C6
  C11   C8

IMPROPER
  C13  C14  C15   O6
  C15  C10  C14   H6
  C4   C14  C10   H4
  C9   C15  C13   O5
  C13   C4   C9   H3
   C9  C10   C4   C1
   C4   C5   C1   O1
   C6   C1   C5   O2
   C2   C5   C6   O3
   C8   C2   C3   O1
   C3  C11   C8   H2
   C6   C3   C2   C7
   C2  C12   C7   H1
   C7  C11  C12   H5
   C8  C12  C11   O4

DONE
STOP
```

**ISOPROPYLIDENE**

```
 0    0    2
iso   INT  0
CORRECT     OMIT DU   BEG
  0.0000
    1  DUMM  DU    M    0  -1  -2     0.000      .0        .0      .00000
    2  DUMM  DU    M    1   0  -1     1.449      .0        .0      .00000
    3  DUMM  DU    M    2   1   0     1.523  111.21        .0      .00000
    4  O4    oh    M    3   2   1     1.540  111.208 -180.000 -0.687053
    5  H12   ho    E    4   3   2     0.947   77.129  161.173  0.445360
    6  C2    c3    M    4   3   2     1.400   59.202   38.147  0.257662
    7  H2    h1    E    6   4   3     1.083  111.663  109.574  0.066303
    8  C1    c3    M    6   4   3     1.523  110.626  -15.119  0.077452
    9  H1    h1    E    8   6   4     1.084  113.024   38.150  0.072737
   10  O2    os    M    8   6   4     1.398  110.056  163.636 -0.524640
   11  C5    c3    M   10   8   6     1.407  110.007  134.558  0.856717
   12  C7    c3    3   11  10   8     1.516  108.940 -137.462 -0.582843
   13  H6    hc    E   12  11  10     1.083  110.075  178.730  0.153328
   14  H7    hc    E   12  11  10     1.084  110.282  -61.167  0.153328
   15  H8    hc    E   12  11  10     1.082  109.839   59.080  0.153328
   16  C8    c3    3   11  10   8     1.521  110.315   98.301 -0.582843
   17  H9    hc    E   16  11  10     1.085  110.203  -56.776  0.153328
   18  H10   hc    E   16  11  10     1.083  110.409   63.222  0.153328
   19  H11   hc    E   16  11  10     1.084  110.261 -176.250  0.153328
   20  O3    os    M   11  10   8     1.408  105.495  -19.403 -0.515798
   21  C3    c3    M   20  11  10     1.385  111.508    5.836  0.369698
   22  H3    h2    E   21  20  11     1.078  108.688  133.204  0.064099
   23  O1    os    M   21  20  11     1.389  113.222 -106.866 -0.318055
   24  C4    c3    M   23  21  20     1.418  111.688  105.423 -0.240033
   25  H4    h1    E   24  23  21     1.085  109.132  -88.275  0.144314
   26  C6    c3    M   24  23  21     1.525  106.984  153.788  0.362813
   27  O5    oh    S   26  24  23     1.402  108.704   58.188 -0.652572
   28  H15   ho    E   27  26  24     0.949  108.270  -58.800  0.406013
   29  H5    h1    E   26  24  23     1.083  108.820  -62.503  0.082799
   30  C9    c3    M   26  24  23     1.516  112.436  177.139  0.219850
   31  H13   h1    E   30  26  24     1.087  108.667  -69.811  0.008596
   32  H14   h1    E   30  26  24     1.087  109.872   48.669  0.008596
   33  O6    oh    M   30  26  24     1.401  108.363  169.230 -0.706952
   34  H16   ho    E   33  30  26     0.946  109.672  163.240  0.447810

LOOP
   C4   C2
   C3   C1

IMPROPER

DONE
STOP
```

**CHRYSIN**

```
0    0    2
chr   INT  0
CORRECT     OMIT DU   BEG
  0.0000
    1  DUMM  DU    M    0  -1  -2     0.000      .0        .0      .00000
    2  DUMM  DU    M    1   0  -1     1.449      .0        .0      .00000
    3  DUMM  DU    M    2   1   0     1.523  111.21        .0      .00000
    4  O1    os    M    3   2   1     1.540  111.208 -180.000 -0.394320
    5  C2    ca    M    4   3   2     1.351    4.784  173.885  0.713312
    6  C8    ca    B    5   4   3     1.377  116.490   -2.352 -0.740947
    7  C9    ca    B    6   5   4     1.388  117.543  179.782  0.715629
    8  O4    oh    S    7   6   5     1.339  116.833 -179.975 -0.623343
    9  H10   ho    E    8   7   6     0.947  111.002  179.919  0.445884
   10  C10   ca    B    7   6   5     1.390  121.847    0.082 -0.784692
   11  C6    ca    S   10   7   6     1.382  119.566   -0.055  0.754196
   12  O2    oh    S   11  10   7     1.324  118.324  179.967 -0.641071
   13  H9    ho    E   12  11  10     0.961  109.457 -179.886  0.474975
   14  H3    ha    E   10   7   6     1.075  121.492  179.951  0.267492
   15  H2    ha    E    6   5   4     1.071  121.382    0.110  0.276922
   16  C1    ca    M    5   4   3     1.395  120.524  177.518 -0.813012
   17  C4    c     M   16   5   4     1.457  119.821    0.438  0.985603
   18  O3    o     E   17  16   5     1.216  122.870  179.967 -0.671988
   19  C7    cd    M   17  16   5     1.457  114.768    0.419 -0.746490
   20  H1    ha    E   19  17  16     1.070  117.703  177.533  0.243339
   21  C3    cc    M   19  17  16     1.336  121.131   -1.287  0.512772
   22  C5    ca    M   21  19  17     1.480  125.728 -179.722 -0.021261
   23  C11   ca    M   22  21  19     1.392  120.634   27.441 -0.139472
   24  H4    ha    E   23  22  21     1.074  120.092    2.345  0.146088
   25  C13   ca    M   23  22  21     1.383  120.406 -178.972 -0.137675
   26  H6    ha    E   25  23  22     1.075  119.674 -179.626  0.142554
   27  C15   ca    M   25  23  22     1.385  120.120   -0.111 -0.117238
   28  H8    ha    E   27  25  23     1.075  120.096 -179.952  0.141247
   29  C14   ca    M   27  25  23     1.386  119.834   -0.330 -0.137675
   30  H7    ha    E   29  27  25     1.075  120.122 -179.800  0.142554
   31  C12   ca    M   29  27  25     1.384  120.164    0.117 -0.139472
   32  H5    ha    E   31  29  27     1.073  119.850 -179.868  0.146088

LOOP
   C3   O1
   C1   C6
  C12   C5

IMPROPER
   C1   C8   C2   O1
   C2   C9   C8   H2
   C8  C10   C9   O4
   C6   C9  C10   H3
   C1  C10   C6   O2
   C4   C6   C1   C2
   C1   C7   C4   O3
   C4   C3   C7   H1
   C5   C7   C3   O1
   C11 C12   C5   C3
   C5  C13  C11   H4
   C11 C15  C13   H6
   C13 C14  C15   H8
   C15 C12  C14   H7
   C5  C14  C12   H5

DONE
STOP
```

**CINCHONINE**

```
0    0    2
cin   INT  0
CORRECT     OMIT DU   BEG
  0.0000
    1  DUMM  DU    M    0  -1  -2     0.000      .0        .0      .00000
    2  DUMM  DU    M    1   0  -1     1.449      .0        .0      .00000
    3  DUMM  DU    M    2   1   0     1.523  111.21        .0      .00000
    4  C19   ca    M    3   2   1     1.540  111.208 -180.000 -0.056314
    5  C18   ca    B    4   3   2     1.415  157.268  105.053 -0.195717
    6  C15   ca    B    5   4   3     1.358  120.513 -106.061 -0.120792
    7  C11   ca    E    6   5   4     1.423  121.127    0.317 -0.361510
    8  H18   ha    E    6   5   4     1.070  118.992 -179.269  0.133897
    9  H21   ha    E    5   4   3     1.075  119.619   74.228  0.147112
   10  H22   ha    E    4   3   2     1.075   56.781 -166.891  0.139570
   11  C17   ca    M    4   3   2     1.356   68.320  -11.256 -0.429143
   12  H20   ha    E   11   4   3     1.074  121.940  -23.559  0.196013
   13  C14   ca    M   11   4   3     1.420  120.662  156.326  0.800905
   14  N2    nb    M   13  11   4     1.355  117.060 -179.630 -0.772835
   15  C16   ca    M   14  13  11     1.291  117.774  179.374  0.462923
   16  H19   h4    E   15  14  13     1.077  116.827 -179.795  0.053914
   17  C13   ca    M   15  14  13     1.413  123.979   -0.077 -0.577140
   18  H17   ha    E   17  15  14     1.072  119.356 -178.921  0.205873
   19  C10   ca    M   17  15  14     1.362  119.960    0.680  0.315798
   20  C8    c3    M   19  17  15     1.527  118.581 -179.842  0.053249
   21  O1    oh    S   20  19  17     1.407  110.613   35.917 -0.714206
   22  H14   ho    E   21  20  19     0.947  109.583   57.109  0.459897
   23  H12   h1    E   20  19  17     1.083  109.882  156.110  0.104786
   24  C2    c3    M   20  19  17     1.536  112.078  -81.267  0.400379
   25  C3    c3    B   24  20  19     1.548  114.079  168.703 -0.295178
   26  H3    hc    E   25  24  20     1.085  111.318   26.336  0.090512
   27  H4    hc    E   25  24  20     1.084  110.238  -91.148  0.090512
   28  H2    h1    E   24  20  19     1.083  105.230   50.166  0.071618
   29  N1    n3    M   24  20  19     1.464  112.424  -65.199 -0.552381
   30  C6    c3    M   29  24  20     1.463  111.457  -82.802 -0.007803
   31  H8    h1    E   30  29  24     1.083  108.627  168.281  0.068546
   32  H9    h1    E   30  29  24     1.083  108.938   52.650  0.068546
   33  C4    c3    M   30  29  24     1.556  111.848  -68.994  0.151980
   34  C9    c2    B   33  30  29     1.505  112.315  143.081 -0.266727
   35  C12   c2    B   34  33  30     1.319  124.936  118.013 -0.442920
   36  H15   ha    E   35  34  33     1.075  121.748 -179.737  0.185890
   37  H16   ha    E   35  34  33     1.078  121.893    0.393  0.185890
   38  H13   ha    E   34  33  30     1.080  116.603  -61.309  0.179373
   39  H5    hc    E   33  30  29     1.087  109.090  -98.144  0.057508
   40  C1    c3    M   33  30  29     1.544  105.750   17.652  0.031193
   41  H1    hc    E   40  33  30     1.085  110.352  169.913  0.043887
   42  C5    c3    M   40  33  30     1.539  106.926  -69.633 -0.127921
   43  H6    hc    E   42  40  33     1.086  110.548  -69.701  0.041704
   44  H7    hc    E   42  40  33     1.087  109.979  172.655  0.041704
   45  C7    c3    M   42  40  33     1.553  107.706   51.627 -0.033214
   46  H10   h1    E   45  42  40     1.084  111.422  136.455  0.085311
   47  H11   h1    E   45  42  40     1.084  111.103 -104.380  0.085311

LOOP
  C14  C11
  C10  C11
   C1   C3
   C7   N1

IMPROPER
  C17  C18  C19  H22
  C19  C15  C18  H21
  C18  C11  C15  H18
  C14  C10  C11  C15
  C14  C19  C17  H20
  C17  C11  C14   N2
  C13  H19  C16   N2
  C16  C10  C13  H17
   C8  C13  C10  C11
  C12   C4   C9  H13
   C9  H15  C12  H16

DONE
STOP
```

**CHLORALOSE**

```
0    0    2
chl   INT  0
CORRECT     OMIT DU   BEG
  0.0000
    1  DUMM  DU    M    0  -1  -2     0.000      .0        .0      .00000
    2  DUMM  DU    M    1   0  -1     1.449      .0        .0      .00000
    3  DUMM  DU    M    2   1   0     1.523  111.21        .0      .00000
    4  O4    oh    M    3   2   1     1.540  111.208 -180.000 -0.710466
    5  H7    ho    E    4   3   2     0.950  147.813   70.491  0.460986
    6  C2    c3    M    4   3   2     1.396   45.891   27.178  0.284952
    7  H2    h1    E    6   4   3     1.082  111.671   99.250  0.066102
    8  C1    c3    M    6   4   3     1.517  106.877  -22.883  0.090289
    9  H1    h1    E    8   6   4     1.081  112.689   33.187  0.098550
   10  O2    os    M    8   6   4     1.409  110.330  157.861 -0.307799
```

```
  11  C6    c3    M   10   8   6     1.377   107.822   139.754  0.043174
  12  C8    c3    3   11  10   8     1.544   110.554  -149.949 -0.473436
  13  Cl1   cl    E   12  11  10     1.764   111.805    59.956  0.096065
  14  Cl2   cl    E   12  11  10     1.773   108.081  -179.270  0.096065
  15  Cl3   cl    E   12  11  10     1.773   108.266   -61.146  0.096065
  16  H6    h2    E   11  10   8     1.085   111.248    92.038  0.221159
  17  O3    os    M   11  10   8     1.381   107.145   -28.668 -0.338111
  18  C4    c3    M   17  11  10     1.399   110.038    15.981  0.556566
  19  H4    h2    E   18  17  11     1.078   108.122   126.148  0.013571
  20  O1    os    M   18  17  11     1.380   112.799  -113.641 -0.442770
  21  C3    c3    M   20  18  17     1.417   112.000   107.802 -0.002092
  22  H3    h1    E   21  20  18     1.083   109.701   -94.243  0.117112
  23  C5    c3    M   21  20  18     1.520   107.647   145.703  0.147761
  24  O5    oh    S   23  21  20     1.407   107.018  -164.555 -0.709500
  25  H10   ho    E   24  23  21     0.950   108.096  -162.526  0.471429
  26  H5    h1    E   23  21  20     1.087   107.786   -46.305  0.059532
  27  C7    c3    M   23  21  20     1.520   112.852    74.301  0.236756
  28  H8    h1    E   27  23  21     1.084   110.700   -61.189  0.049909
  29  H9    h1    E   27  23  21     1.082   109.746    58.636  0.049909
  30  O6    oh    M   27  23  21     1.409   109.775   175.032 -0.718155
  31  H11   ho    E   30  27  23     0.947   109.824    81.808  0.446377

LOOP
   C3   C2
   C4   C1

IMPROPER

DONE
STOP
```

**RUTIN**

```
0    0    2
rut   INT  0
CORRECT     OMIT DU   BEG
  0.0000
   1  DUMM  DU    M    0  -1  -2     0.000      .0        .0      .00000
   2  DUMM  DU    M    1   0  -1     1.449      .0        .0      .00000
   3  DUMM  DU    M    2   1   0     1.523   111.21       .0      .00000
   4  O16   oh    M    3   2   1     1.540   111.208  -180.000 -0.582105
   5  H30   ho    E    4   3   2     0.951    55.194   108.899  0.428598
   6  C27   ca    M    4   3   2     1.342   135.444  -169.657  0.299035
   7  C26   ca    B    6   4   3     1.384   119.724   127.673 -0.312422
   8  C22   ca    S    7   6   4     1.380   119.739   178.631 -0.163953
   9  H24   ha    E    8   7   6     1.071   118.985  -178.594  0.161313
  10  H26   ha    E    7   6   4     1.074   119.049    -0.226  0.207108
  11  C25   ca    M    6   4   3     1.393   120.542   -51.984  0.337480
  12  O15   oh    S   11   6   4     1.369   117.986    -2.053 -0.655664
  13  H29   ho    E   12  11   6     0.956   110.254    82.102  0.429841
  14  C21   ca    M   11   6   4     1.377   120.494  -178.628 -0.275218
  15  H23   ha    E   14  11   6     1.070   118.608   178.577  0.212249
  16  C17   ca    M   14  11   6     1.397   120.235    -0.559 -0.068439
  17  C14   cd    M   16  14  11     1.477   122.110   175.507  0.390804
  18  O11   os    M   17  16  14     1.354   110.634  -154.006 -0.308444
  19  C18   ca    M   18  17  16     1.340   123.588   177.607  0.477825
  20  C20   ca    B   19  18  17     1.384   115.369   178.379 -0.637820
  21  C24   ca    B   20  19  18     1.378   117.787   179.232  0.760210
  22  O14   oh    S   21  20  19     1.339   117.508  -179.901 -0.655616
  23  H28   ho    E   22  21  20     0.947   111.437   179.171  0.462647
  24  C23   ca    B   21  20  19     1.395   121.109     0.144 -0.866403
  25  C19   ca    S   24  21  20     1.381   120.088    -0.182  0.761298
  26  O13   oh    S   25  24  21     1.330   120.888   179.783 -0.646860
  27  H27   ho    E   26  25  24     0.948   110.761     0.920  0.466871
  28  H25   ha    E   24  21  20     1.077   120.075   179.823  0.240965
  29  H22   ha    E   20  19  18     1.071   120.919    -0.080  0.257968
  30  C16   ca    M   19  18  17     1.392   121.052    -2.159 -0.601638
  31  C15   c     M   30  19  18     1.465   118.619     0.399  0.743891
  32  O12   o     E   31  30  19     1.204   124.767  -176.927 -0.536824
  33  C13   cc    M   31  30  19     1.473   114.933     3.662 -0.153286
  34  O6    os    M   33  31  30     1.363   115.537   179.806 -0.301840
  35  C9    c3    M   34  33  31     1.392   117.807   -73.827  0.064696
  36  C4    c3    3   35  34  33     1.526   108.142   142.544  0.279095
  37  O7    oh    S   36  35  34     1.387   111.734   -67.445 -0.644865
  38  H18   ho    E   37  36  35     0.953   110.664    23.790  0.411328
  39  C3    c3    3   36  35  34     1.525   109.327   172.755  0.212614
  40  O5    oh    S   39  36  35     1.395   108.766  -170.076 -0.647244
  41  H17   ho    E   40  39  36     0.948   108.589   173.181  0.406477
  42  C2    c3    B   39  36  35     1.524   111.999   -48.110  0.357072
  43  O4    oh    S   42  39  36     1.408   110.586   173.525 -0.683409
  44  H16   ho    E   43  42  39     0.949   109.685   -58.513  0.395844
  45  H2    h1    E   42  39  36     1.079   108.584   -70.771  0.023874
  46  H3    h1    E   39  36  35     1.093   106.991    71.016  0.004297
  47  H4    h1    E   36  35  34     1.087   107.461    54.838  0.062785
  48  H9    h2    E   35  34  33     1.086   109.962    22.195  0.102675
  49  O1    os    M   35  34  33     1.395   107.786   -97.122 -0.330505
  50  C1    c3    M   49  35  34     1.404   113.780   177.499  0.050028
  51  H1    h1    E   50  49  35     1.094   109.216   -57.208  0.034692
  52  C10   c3    M   50  49  35     1.516   108.959  -173.599  0.134897
  53  H10   h1    E   52  50  49     1.084   109.313    48.628  0.060974
  54  H11   h1    E   52  50  49     1.085   109.008   167.574  0.060974
  55  O2    os    M   52  50  49     1.404   109.451   -72.677 -0.503958
  56  C6    c3    M   55  52  50     1.376   116.459  -170.490  0.397609
  57  H6    h2    E   56  55  52     1.084   111.055    45.164  0.090817
  58  O3    os    M   56  55  52     1.395   112.772   -72.830 -0.603945
  59  C11   c3    M   58  56  55     1.422   115.882   -56.802  0.609855
  60  C12   c3    3   59  58  56     1.517   106.861   177.613 -0.367638
  61  H13   hc    E   60  59  58     1.083   109.973   -61.156  0.092080
  62  H14   hc    E   60  59  58     1.082   110.743   178.738  0.092080
  63  H15   hc    E   60  59  58     1.084   110.258    58.198  0.092080
  64  H12   h1    E   59  58  56     1.084   109.132    58.513 -0.048741
  65  C8    c3    M   59  58  56     1.527   108.614   -60.034  0.083657
  66  O10   oh    S   65  59  58     1.409   109.073   173.174 -0.719446
  67  H21   ho    E   66  65  59     0.951   107.556  -174.891  0.471870
  68  H8    h1    E   65  59  58     1.088   108.485   -68.366  0.040282
  69  C7    c3    M   65  59  58     1.525   112.040    51.633  0.342411
  70  O9    oh    S   69  65  59     1.402   109.453  -174.216 -0.730123
  71  H20   ho    E   70  69  65     0.951   108.043    85.247  0.452088
  72  H7    h1    E   69  65  59     1.082   108.544    69.549  0.055020
  73  C5    c3    M   69  65  59     1.523   110.796   -50.431  0.038153
  74  H5    h1    E   73  69  65     1.080   110.160   173.114  0.138937
  75  O8    oh    M   73  69  65     1.409   110.028   -69.495 -0.670021
  76  H19   ho    E   75  73  69     0.950   109.376    85.208  0.419061

LOOP
  C17  C22
  C13  C14
  C16  C19
   C1   C2
   C5   C6

IMPROPER
  C25  C26  C27  O16
  C27  C22  C26  H26
  C17  C26  C22  H24
  C21  C27  C25  O15
  C25  C17  C21  H23
  C21  C22  C17  C14
  C17  C13  C14  O11
  C20  C16  C18  O11
  C18  C24  C20  H22
  C20  C23  C24  O14
  C24  C19  C23  H25
  C23  C16  C19  O13
  C15  C18  C16  C19
  C16  C13  C15  O12
  C15  C14  C13   O6

DONE
STOP
```

## References Cited for Supporting Information

Bekers, O., Uijtendaal, E., Beijnen, J., Bult, A. and Underberg, W. (1991) Cyclodextrins in the pharmaceutical field. *Drug Development and Industrial Pharmacy* **17,** 1503-1549.

Berland, K., Renaud, J.B. and Mayer, P.M. (2015) Utilizing ion mobility and tandem mass spectrometry to evaluate the structure and behaviour of multimeric cyclodextrin complexes. *Canadian Journal of Chemistry* **93,** 1313-1319. https://doi.org/10.1139/cjc-2014-0419

Christoforides, E., Papaioannou, A. and Bethanis, K. (2018) Crystal structure of the inclusion complex of cholesterol in β-cyclodextrin and molecular dynamics studies. *Beilstein Journal of Organic Chemistry* **14,** 838-848. https://doi.org/10.3762/bjoc.14.69

Guo, M., Song, F., Liu, Z. and Liu, S. (2004) Characterization of non-covalent complexes of rutin with cyclodextrins by electrospray ionization tandem mass spectrometry. *Journal of Mass Spectrometry* **39,** 594-599.

Lee, S.J.C., Lee, J.W., Lee, H.H., Seo, J., Noh, D.H., Ko, Y.H., Kim, K. and Kim, H.I. (2013) Host–guest chemistry from solution to the gas phase: an essential role of direct interaction with water for high-affinity binding of cucurbit [n] urils. *The Journal of Physical Chemistry B* **117,** 8855-8864.

Lipinski, C.A., Lombardo, F., Dominy, B.W. and Feeney, P.J. (2012) Experimental and computational approaches to estimate solubility and permeability in drug discovery and development settings. *Advanced Drug Delivery Reviews* **64,** 4-17. https://doi.org/10.1016/j.addr.2012.09.019

Makedonopoulou, S., Papaioannou, J., Argyroglou, I. and Mavridis, I.M. (2000) Crystal structures of the inclusion complexes of β-cyclodextrin with aliphatic monoacids tridecanoic acid and (Z)-tetradec-7-enoic acid. Formation of [3] pseudorotaxanes. *Journal of Inclusion Phenomena and Macrocyclic Chemistry* **36,** 191-215. https://doi.org/10.1023/A:1008055904136

May, J.C., Morris, C.B. and McLean, J.A. (2017) Ion Mobility Collision Cross Section Compendium. *Analytical Chemistry* **89,** 1032-1044. https://doi.org/10.1021/acs.analchem.6b04905

Stow, S.M., Causon, T.J., Zheng, X., Kurulugama, R.T., Mairinger, T., May, J.C., Rennie, E.E., Baker, E.S., Smith, R.D., McLean, J.A., Hann, S. and Fjeldsted, J.C. (2017) An Interlaboratory Evaluation of Drift Tube Ion Mobility-Mass Spectrometry Collision Cross Section Measurements. *Analytical Chemistry* **89,** 9048-9055. https://doi.org/10.1021/acs.analchem.7b01729

Yu, Z., Cui, M., Yan, C., Song, F., Liu, Z. and Liu, S. (2007) Investigation of heptakis (2, 6-di-O-methyl)-β-cyclodextrin inclusion complexes with flavonoid glycosides by electrospray ionization mass spectrometry. *Rapid Communications in Mass Spectrometry* **21,** 683-690.

Zlibut, E., May, J.C., Wei, Y., Gessmann, D., Wood, C.S., Bernat, B.A., Pugh, T.E., Palmer-Jones, L., Cosquer, R.P. and Dybeck, E. (2023) Noncovalent Host–Guest Complexes of Artemisinin with α-, β-, and γ-Cyclodextrin Examined by Structural Mass Spectrometry Strategies. *Analytical Chemistry*. https://doi.org/10.1021/acs.analchem.2c05076
